# Furan Release via Force-Promoted Retro-[4+2][3+2] Cycloaddition

**DOI:** 10.1021/jacs.3c08771

**Published:** 2023-09-15

**Authors:** Kamil Suwada, Alice Weng Ieong, Hei Lok Herman Lo, Guillaume De Bo

**Affiliations:** Department of Chemistry, University of Manchester, Oxford Road, Manchester, M13 9PL, United Kingdom

## Abstract

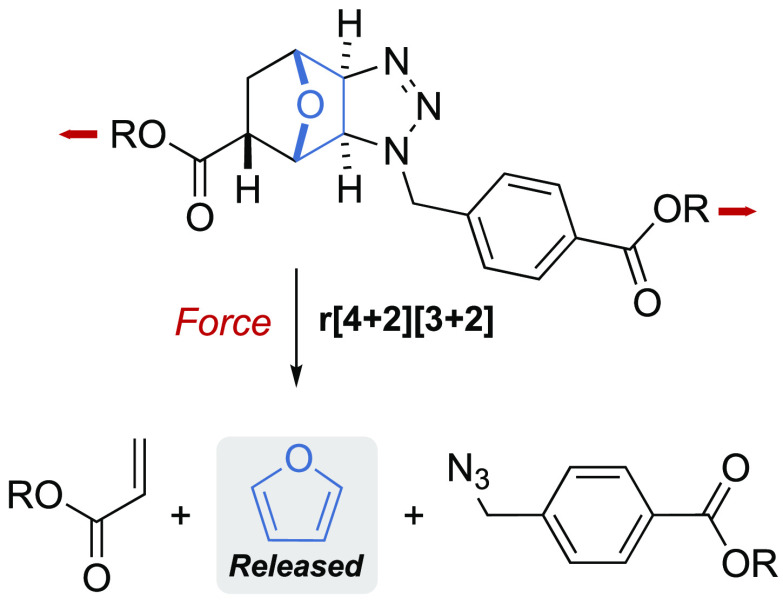

Mechanophores (mechanosensitive molecules) have been instrumental in the development of various force-controlled release systems. However, the release of functional organic molecules is often the consequence of a secondary (nonmechanical) process triggered by an initial bond scission. Here we present a new mechanophore, built around an oxanorbornane-triazoline core, that is able to release a furan molecule following a force-promoted double retro-[4+2][3+2] cycloaddition. We explored this unprecedented transformation experimentally (sonication) and computationally (DFT, CoGEF) and found that the observed reactivity is controlled by the geometry of the adduct, as this reaction pathway is only accessible to the *endo-exo-cis* isomer. These results further demonstrate the unique reactivity of molecules under tension and offer a new mechanism for the force-controlled release of small molecules.

The mechanochemical release of cargo molecules offers great promise for the development various controlled-release applications.^1^ This can be achieved by using polymers to actuate a mechanophore, the activation of which results in the direct or indirect release of a cargo molecule.^2^ The release of functional organic molecules is often the result of a nonmechanical secondary process (e.g., transesterification, fragmentation) following an initial mechanochemical bond scission (i.e., indirect).^3−6^ In contrast, direct release is so far limited to simple molecules (e.g., N_2_) and ions,^7−12^ though more elaborate cargo can be released by flex-activation but at the expense of efficiency.^13−17^ In this context, we were intrigued by the possibility of promoting the direct release of a small molecule via a double retrocycloaddition that would detach both actuating polymers from the cargo in a single elongation event.

Here we show that a new mechanophore, built around an oxanorbornane-triazoline core, can release a furan molecule, “trapped” between an acrylate and an azide, via a double retro-[4+2][3+2] cycloaddition (Figure 1). We found that this unprecedented mechanochemical reactivity is controlled by the geometry of the adduct,^18−27^ as the retro-[4+2][3+2] pathway is only observed in the *endo-exo-cis* isomer, while the *exo-exo-cis* isomer only dissociates via a retro-[3+2] cycloaddition. These results provide new insight into the unique reactivity of molecules under tension and offer a novel mechanism for the direct release of small organic molecules.

**Figure 1:**
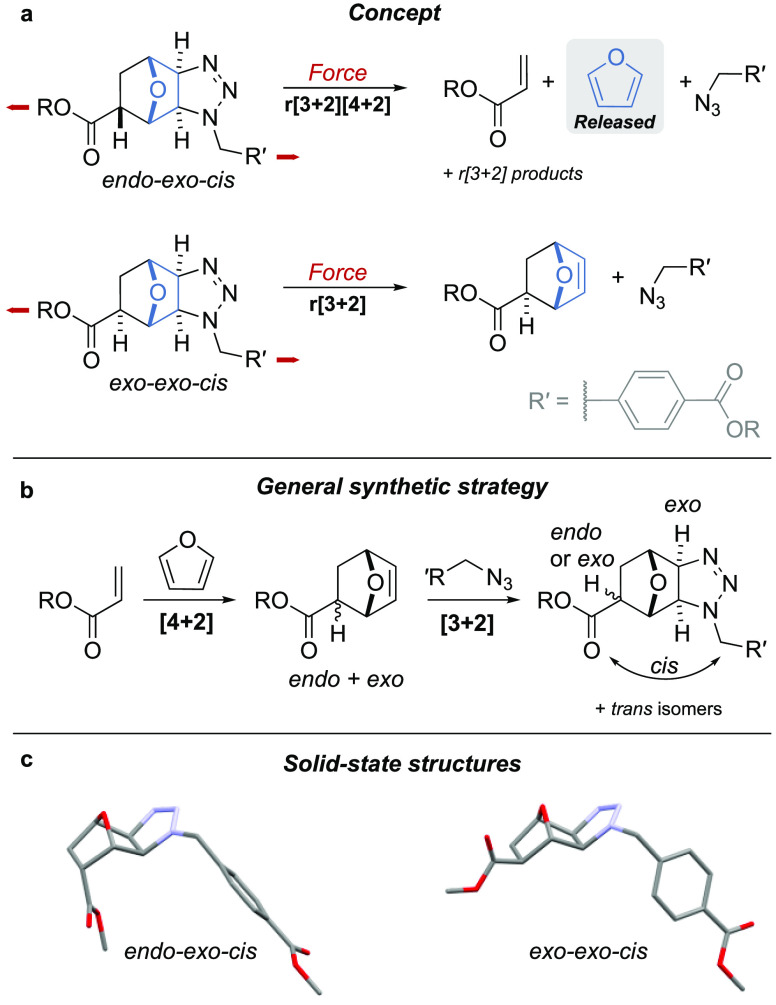
Furan release via force-promoted retro-[4+2][3+2]cycloaddition. (a) Mechanical activation of *endo-exo-cis* and *exo-exo-cis* adducts. Red arrows indicate the direction of the force. (b) Assembly of the adducts by sequential [4+2] and [3+2] cycloadditions. (c) Solid-state structure (XRD) of *endo-exo-cis* and *exo-exo-cis* adducts (R = Me).

Our new mechanophore was formed by sequential [4+2] cycloaddition (Diels–Alder) between a furan ring and an acrylate derivative, followed by a [3+2] cycloaddition (Huisgen) between the resulting oxanorbornene and an organic azide to form a triazoline ring (Figure 1b). As each cycloaddition can produce both *endo* and *exo* adducts and the triazoline ring can adopt a *cis* or *trans* orientation in relation to the ester moiety, a total of 8 isomers can theoretically be produced. In practice, only 4 isomers are isolated, as the [3+2] cycloaddition always affords the *exo* isomer (the identity of the 4 isomers was confirmed by XRD and NMR, see Figure 1c and SI Section 8). As both *trans* isomers are mechanically inert (see Figures S19 and S21), we decided to focus our investigation on the *endo-exo-cis* and *exo-exo-cis* adducts (Figure 1a).

Chain-centered adducts were obtained by single electron transfer living radical polymerization (SET-LRP)^28^ of methyl acrylate (see SI Section 4), and their mechanical activation was performed in acetonitrile at 5–10 °C, using high-intensity ultrasound, until complete scission of the starting polymer was confirmed by GPC (see Figure 2a,b and Figures S5–S8). ^1^H NMR analysis of the sonicated sample confirms that the *exo-exo-cis* isomer undergoes a retro-[3+2] cycloaddition, as evidenced by the shifting of the peaks of the benzylic azide (*a*, *b*, *c*, Figure 2c-v) and the emergence of the diagnostic signals pertaining to the olefinic (*l*, *m*, Figure 2c-vi) and bridging (*k*, *n*, Figure 2c-vi) protons of the *exo*-oxanorbornene unit in the postsonication samples (Figure 2c-vii).

**Figure 2:**
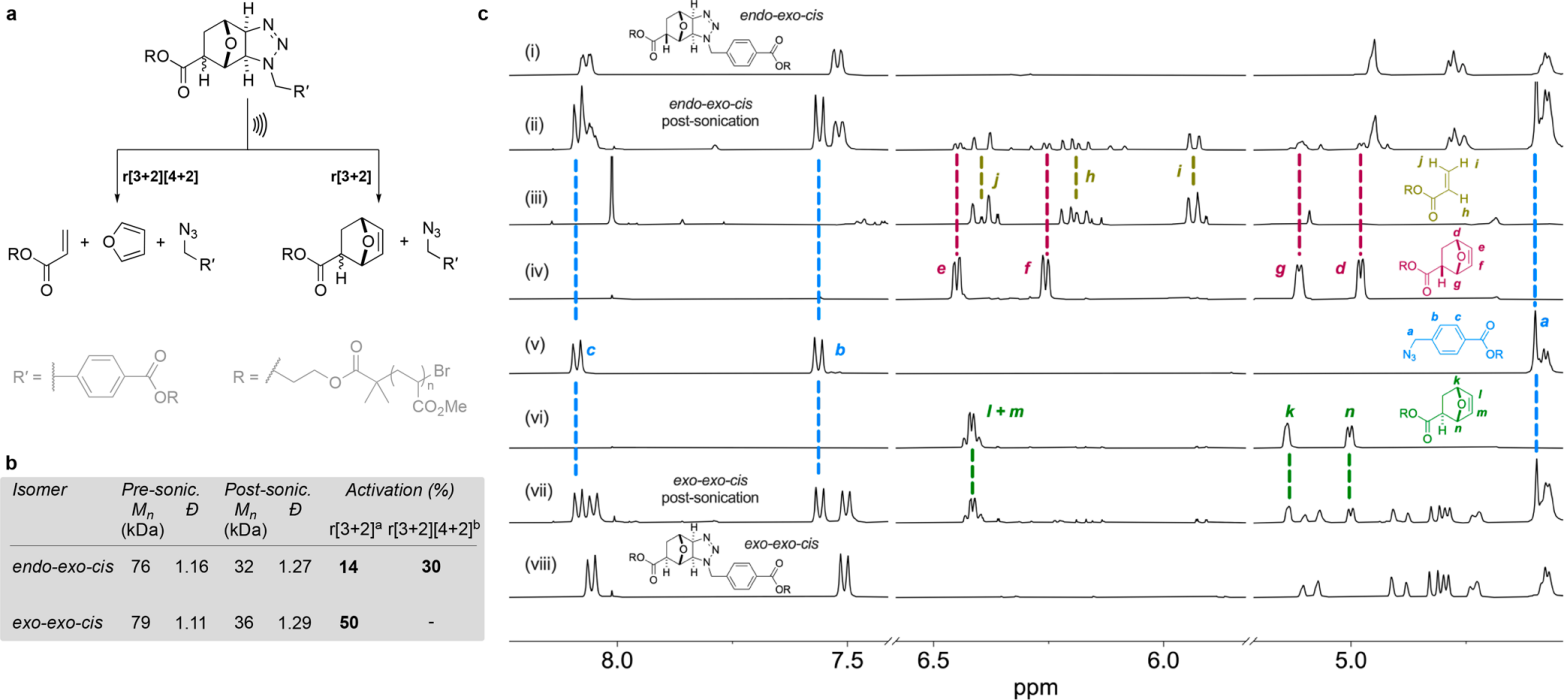
(a) Mechanical activation of chain-centered *endo-exo-cis* and *exo-exo-cis* mechanophores. Conditions: US (20 kHz, 13.0 W/cm^2^, 1 s ON/1 s OFF), CH_3_CN, 5–10 °C, 240 min. (b) Structural and activation parameters of the sonicated polymers. ^a^Determined by integrating protons *e*, *f* or *l*, *m* against the aromatic peaks of the intact mechanophore. ^b^Determined by integrating proton *h*, *i*, and *j* against the aromatic peaks of the intact mechanophore. (c) Partial ^1^H NMR (500 MHz, acetone-*d*_6_, 298 K, 1024 scans) spectra of the *endo-exo-cis* adduct before (i) and after (ii) sonication and of the *exo-exo-cis* adduct before (viii) and after (vii) sonication, along with reference compounds **P-S14b** (iii), **P-S14** (iv), **P-S5** (v), and **P-S15** (vi).

The picture is more complicated for the *endo-exo-cis* isomer, as several signals crowd the olefinic region of the postsonication ^1^H NMR spectrum (6.5–5.9 ppm, Figure 2c-ii). The dominant signals can be attributed to an acrylate moiety, identified by the diagnostic pattern of the terminal olefin (*j*, *h*, *i*, Figure 2c-iii), while the minor peaks can be matched with the olefinic (*e*, *f*, Figure 2c-iv) and bridging (*d*, *g*, Figure 2c-iv) protons of the *endo*-oxanorbornene unit. The presence of these two products (along with the formation of the benzylic azide) indicates that two retrocycloaddition processes are taking place (in a ∼2:1 ratio; Figure 2b) during the mechanical activation of the *endo-exo-cis* isomer. The formation of the *endo*-oxanorbornene unit is indicative of a retro-[3+2] cycloaddition, while the presence of the acrylate suggests that the adduct undergoes a double retro-[4+2][3+2] cycloaddition with concomitant release of furan (confirmed by ^1^H NMR, see Figure S25).

Since the *exo-exo-cis* adduct only dissociates via a retro-[3+2] pathway and both *endo-* and *exo*-oxanorbornene adducts are stable under sonication conditions (see Figures S23, S24), it is likely that the divergent dissociation pathways of the *endo-exo-cis* adduct are a consequence of the initial bond cleavage occurring at either C–N bond *c* or C–C bond *a* (Figure 3a), the former leading to the formation of the *endo*-oxanorbornene adduct, and the latter triggering the cascade leading to the release of furan (Figure 3e). The simulated elongation of a model of the *endo-exo-cis* adduct offers some insight into the dissociation process (Figure 3a–d). The elongation profile of this model (Figure 3a), obtained from CoGEF calculations^29^ (DFT ωB97X-D/6-31G*), reveals that the initial scission of C–C bond *a* (iii, Figure 3a,b), connecting the ester to the rest of the adduct (*F*_max_ = 4.9 nN), is quickly followed by the collapse of the resulting intermediate (iv, Figure 3a,b), which releases a molecule of furan and regenerates the terminal acrylate and benzylic azide groups (Figure 3e). The CoGEF calculation suggests a sequential polar mechanism for this process (see SI Section 9.2), and such a mechanism has been previously hypothesized for similar retrocycloadditions.^30,31^ However, as the CoGEF method does not account for dynamic or thermal effects, which can play a significant role in the dissociation and selectivity mechanisms of mechanophores,^20,32−35^ a different mechanism cannot be excluded. Indeed, even though bond *a* is predicted to cleave preferentially, the elongation of bond *c* is substantial at *E*_max_ (Figure 3a–c), which explains the formation of *endo*-oxanorbornene as a minor product. The presence of the retro-[4+2][3+2] pathway originates from the geometry of the *endo-exo-cis* adduct, where the ester moiety is *anti* to the bridging oxygen of the oxanorbornane core (Figure 1c). Upon elongation, this ester progressively aligns with the rest of the structure (ii; Figure 3b). This induces a substantial amount of torsional stress, which can be visualized by the opening of angle α (Figure 3d), that enhances the coupling at bond *a*. This lever-arm effect^36^ is not observed in the *exo-exo-cis* isomer, as the *syn* orientation of the ester (Figure 1c) allows this adduct to adopt an extended conformation without developing such torsional deformation (see Figure 3d and Figure S37).

**Figure 3:**
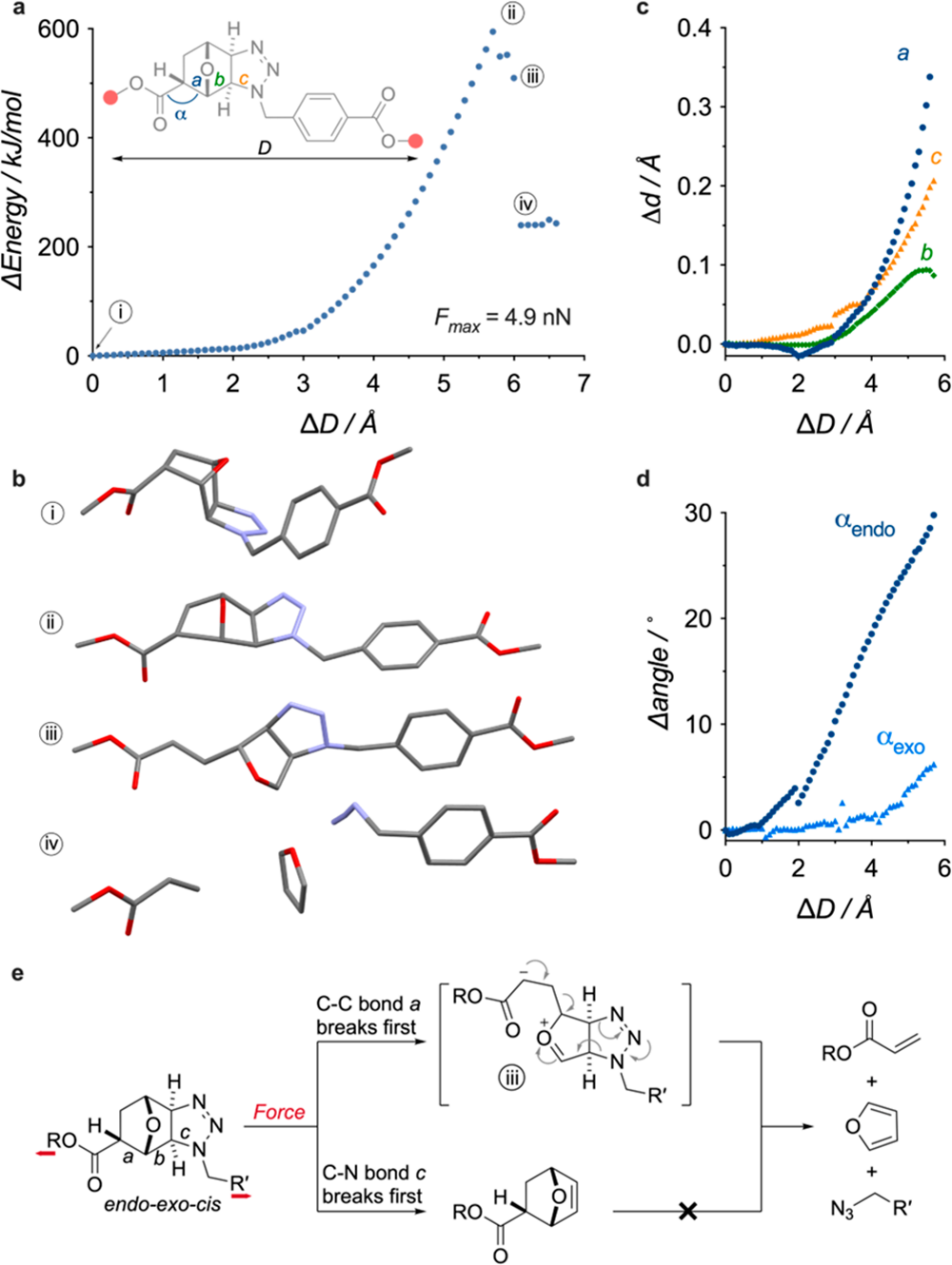
Computational investigation of dissociation of the *endo-exo-cis* adduct. (a) Evolution of energy upon simulated elongation (CoGEF, DFT ωB97X-D/6-31G*) of a model of the *endo-exo-cis* mechanophore. (b) Equilibrium geometries at *E*_0_ (i), *E*_max_ (ii), after the first bond scission (iii), and after furan release (iv). (c) Elongation of bonds *a*, *b*, and *c*, upon simulated elongation of the same model. (d) Comparison of the opening of angle α during the simulated elongation of models of the *endo-exo-cis* and *exo-exo-cis* adducts. (e) Possible dissociation mechanism.

In conclusion, we have described a new mechanophore that can undergo a double retro-[4+2][3+2] cycloaddition in a single elongation event. This unprecedented dissociation process leads to the release of a small molecule (furan). We anticipate that this new mechanophore should provide a useful platform for the release of more complex molecules and that this mechanism should be amenable to alternative mechanophore architectures. These results further demonstrate the unique reactivity of molecules under tension and offer a new mechanism for the force-controlled release of small molecules.

## Supporting Information Available

Detailed descriptions of CoGEF calculations, synthetic procedures, characterization of new compounds, and spectroscopic data (PDF)
