# Supplementary material for: Furan Release via Force-Promoted Retro-[4+2][3+2] Cycloaddition
Source: J Am Chem Soc. 2023 Sep 15;145(38):20782–5. doi: 10.1021/jacs.3c08771 (PMC10540202; doi:10.1021/jacs.3c08771)
Supplement: Supplementary file 1 — ja3c08771_si_001.pdf [file ja3c08771_si_001.pdf]

Supporting Information

Furan Release via Force-Promoted Retro-[4+2][3+2] Cycloaddition

*Kamil Suwada, Alice Weng Ieong, Hei Lok Herman Lo, and Guillaume De Bo**

Department of Chemistry, University of Manchester, Oxford Road, Manchester, M13 9PL, UK

*E-mail: guillaume.debo@manchester.ac.uk

1 Table of Contents

1 Table of Contents 2

2 General Experimental Details 4

3 Synthesis of mechanophores and reference compounds 5

3.1 Synthetic Route to S1-S5. 5

3.1.1 Synthesis of S3 5

3.1.2 Synthesis of S4 5

3.1.3 Synthesis of S5 6

3.2 Synthetic Route to S6-S11. 7

3.2.1 Synthesis of S6 and S7 7

3.2.2 Synthesis of S8-S11 8

3.3 Synthetic Route to S12-S15. 9

3.3.1 Synthesis of S12 and S13 9

3.3.2 Synthesis of S14 and S15 9

3.4 Synthetic Route to 1-4. 10

3.4.1 Synthesis of 1-4 11

4 Synthesis of Polymers: 13

4.1 Representative procedure: synthesis of P-1 13

4.2 List of polymers 13

5 NMR Spectra 15

5.1 Spectra of S3 15

5.2 Spectra of S4 16

5.3 Spectra of S5 17

5.4 Spectra of S6 18

5.5 Spectra of S7 19

5.6 Spectra of S8 20

5.7 Spectra of S9 21

5.8 Spectra of S10 22

5.9 Spectra of S11 23

5.10 Spectra of S14 24

5.11 Spectra of S15 26

5.12 Spectra of 1 27

5.13 Spectra of 2 28

5.14 Spectra of 3 30

5.15 Spectra of 4 31

5.16 Spectra of P-1 32

5.17 Spectra of P-2 33

5.18 Spectra of P-3 34

5.19 Spectra of P-4 35

5.20 Spectra of P-S5 36

5.21 Spectra of P-S14 37

5.22 Spectra of P-S15 38

5.23 Spectra of PMA 38

5.24 Spectra of Furan 39

6 Sonication Experiments 39
6.1 Standard Sonication Procedure 39
6.2 Sonication Experiments Overview 39
6.3 Kinetic Investigation Overview 42
6.4 Sonication Experiment of Polymer P-1 48
6.5 Sonication Experiment of Polymer P-2 48
6.6 Sonication Experiment of Polymer P-3 50
6.7 Sonication Experiment of Polymer P-4 50
6.8 Sonication Experiment of Polymer P-S14 52
6.9 Sonication Experiment of Polymer P-S15 52
6.10 Sonication Experiment of Polymer P-2 for Furan release 53
6.11 Sonication Experiment of PMA 54
6.12 Post-Sonication NMR Spectra 56

7 Thermal activation of polymers 63
7.1 Thermal activation of P-S14 63
7.2 Thermal activation of P-1 63
7.3 Thermal activation of P-2 64
7.4 Thermal activation of P-3 64
7.5 Thermal activation of P-4 64

8 Crystal structures 66
8.1 Crystal structure of S8 66
8.2 Crystal structure of S9 67
8.3 Crystal structure of S10 68
8.4 Crystal structure of S11 69

9 Calculations 70
9.1 CoGEF of model 1' (*endo-exo-trans*) 70
9.2 CoGEF of model 2' and $2'_{long}$ (*endo-exo-cis*) 71
9.3 CoGEF of model 3' (*exo-exo-trans*) 74
9.4 CoGEF of model 4' (*exo-exo-cis*) 75

10 References 76

2 General Experimental Details

Unless otherwise stated, all reagents and solvents were purchased from commercial suppliers. All other chemicals were used without further purification. Compound **S1** was prepared according to literature procedure.[S1]

Gel permeation chromatography (GPC) analyses were performed in THF solution (~1.0 mg mL-1) at 35 °C using a GPC Agilent 1260 Infinity II with 2 × PL gel 10 µm mixed-B and a PL gel 500 Å column, and equipped with a differential refractive index (DRI) detector employing narrow polydispersity polystyrene standards (Agilent Technologies) as a calibration reference. Samples were filtered through a Whatman Puradisc 4 mm syringe filter with 0.45 µm PTFE membrane before injection to equipment, and experiments were carried out with injection volume of 50 µL, flow rate of 1 mL min^{-1}. Results were analysed using Agilent GPC/SEC Software Version 2.2.

Ultrasound experiments were performed using a Sonics VCX 500 ultrasonic processor set at 25% amplitude and equipped with a 13 mm diameter solid probe or replaceable-tip probe. The distance between the titanium tip and the bottom of the Suslick cell was 3 cm. The ultrasonic intensity was calibrated using the method outlined by Hickenboth *et al.*[S2] The Suslick cells were fabricated by the Department of Chemistry glass workshop at the University of Manchester.

Analytical TLC was performed on precoated silica gel plates (0.25 mm thick, 60 F254, Merck, Germany) and observed under UV light or stained with either potassium permanganate or phosphomolybdic acid solution. Preparative TLC was performed on precoated silica gel plates: 2 mm, UNIPLATE GF, Analtech Inc., DE, USA. Flash column chromatography was performed with silica gel 60 (230-400 mesh) from Sigma-Aldrich. ^{1}H and ^{13}C NMR spectra were obtained using either a Bruker Avance III 500 MHz Prodigy instrument or a Bruker Avance III 400 MHz Prodigy instrument at the University of Manchester. Chemical shifts are reported in parts per million (ppm) from high to low frequency and referenced to the residual solvent resonance. Coupling constants (*J*) are reported in Hertz (Hz) and splitting patterns are designated as follows: br = broad, s = singlet, d = doublet, t = triplet, q = quartet, p = pentet and m = multiplet. ^{1}H and ^{13}C assignments were made using 1D and 2D NMR methods (HSQC, HMBC, COSY). Mass spectra were obtained through the Mass Spectrometry services in the Department of Chemistry at the University of Manchester. XRD structures were obtained through the X-ray crystallography service in the Department of Chemistry at the University of Manchester

Abbreviations: CoGEF: constrained geometries simulate external force; DCM: dichloromethane; DMAP: 4-Dimethylaminopyridine; DMSO: dimethyl sulfoxide; EDCI: 1-Ethyl-3-(3-dimethylaminopropyl)carbodiimide; ESI: electrospray ionization; PE: petroleum ether; HRMS: high resolution mass spectrometry; Me_6TREN: Tris[2-(dimethylamino)ethyl]amine; MS: mass spectrometry; THF: tetrahydrofuran; TLC: thin layer chromatography.

3 Synthesis of mechanophores and reference compounds

3.1 Synthetic Route to S1-S5.

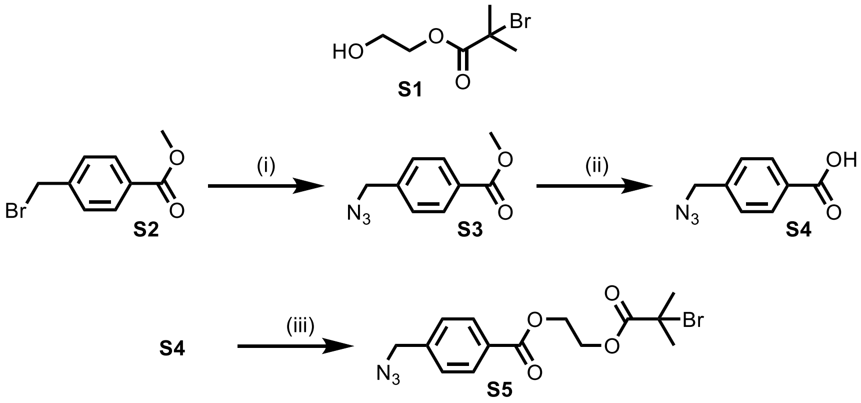

Scheme S1. Synthetic route to **S3-S5.** Conditions: (i) NaN_3, acetone/water, 12h, RT, 97%; (ii) LiOH, THF/MeOH/water, 12h, 50°C, 91%; (iii) EDCl, DMAP, **S1**, DCM, 24h, 87%.

3.1.1 Synthesis of S3

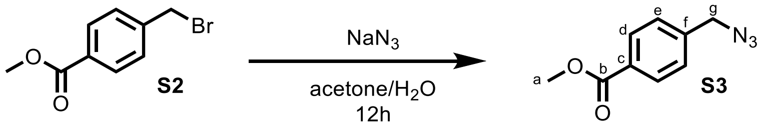

97%

Methyl 4-(bromomethyl)benzoate (5.00 g, 21.83 mmol, 1 equiv.) was added to a round bottom flask (25 mL) and dissolved in acetone (8 mL). Next, sodium azide (1.56 g, 24.01 mmol, 1.1 equiv.) was added to the mixture dissolved in water (2 mL). The resulting mixture was stirred for 12h at room temperature. Next, the reaction was diluted with 30 mL of deionised water and extracted with ethyl acetate (3 x 30 mL). Organic layer was then dried over anhydrous magnesium sulphate, filtered and solvent removed to yield **S3** (4.04 g, 21.18 mmol, 97%) as colourless liquid, which was used without further purification.

^{1}H NMR (500 MHz, $CDCl_3$) δ = 8.09 – 8.02 (m, 2H, H_d), 7.42 – 7.36 (m, 2H, H_e), 4.42 (s, 2H, H_g), 3.92 (s, 3H, H_a).
^{13}C NMR (126 MHz, $CDCl_3$) δ = 166.77 (C_b), 140.53 (C_f), 130.26 (C_e), 130.20 (C_c), 128.07 (C_d), 54.43 (C_g), 52.35 (C_a).
MS-ESI(+): m/z = 214.05 $[M+Na]^+$
HRMS-ESI(+): calculated for $[M+Na]^+$: 214.0587; found: 214.0588.

3.1.2 Synthesis of S4

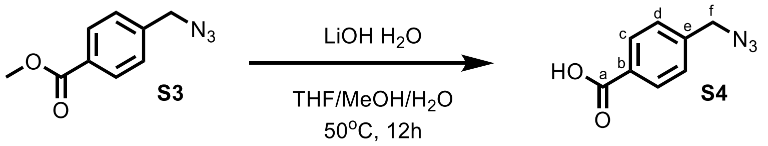

91%

S3 (2.00 g, 10.46 mmol, 1 equiv.) was dissolved in THF (20 mL) and added into a round bottom flask (100 mL) containing LiOH monohydrate (1.25 g, 52.30 mmol, 5 equiv.) in water and methanol mixture (4 and 20 mL, respectively) at room temperature. Setup was equipped with an air condenser and the mixture stirred vigorously at 50°C for 24 hours. Next, mixture was neutralised with acetic

acid and poured onto aqueous solution of HCl (1 M, 30 mL) in a separating funnel, aqueous layer extracted with ethyl acetate (3 x 30 mL) and phases separated. Organic layer was then dried over anhydrous magnesium sulphate, filtered and solvent removed. Crude was subjected to flash column chromatography (PE/EA : 80/20 + 0.5 % AcOH) to yield **S4** (1.68 g, 9.52 mmol, 91%) as white solid.

^{1}H NMR (500 MHz, $CDCl_3$) δ = 8.13 (d, J = 8.3 Hz, 2H, H_c), 7.44 (d, J = 8.0 Hz, 2H, H_d), 4.45 (s, 2H, H_f).
^{13}C NMR (126 MHz, $CDCl_3$) δ = 170.97 (C_a), 141.57 (C_e), 130.92 (C_c), 129.21 (C_b), 128.18 (C_d), 54.43 (C_f).
MS-ESI(-): m/z = 176.04 $[M]^-$
HRMS-ESI(-): calculated for [M]-: 176.0465; found: 176.0456.

3.1.3 Synthesis of S5

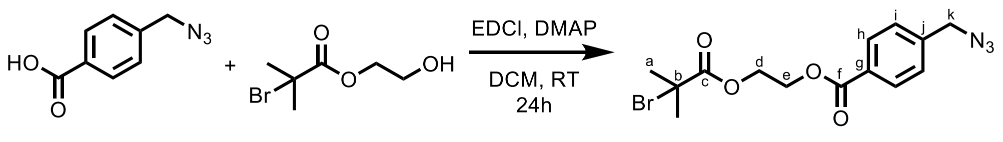

S3 (500 mg, 2.82 mmol, 1 equiv.) was added along **S1** (715 mg, 3.39 mmol, 1.2 equiv.) in DCM (20 mL) into a round bottom flask (50 mL) containing EDC hydrochloride (650 mg, 3.39 mmol, 1.2 equiv.) and 4-DMAP (414 mg, 3.39 mmol, 1.2 equiv.) under nitrogen atmosphere. Reaction was left to stir at room temperature for 24 hours. Next, the mixture was diluted with deionised water (30 mL) and extracted with ethyl acetate (3 x 30 mL). Organic layer was then dried over anhydrous magnesium sulphate, filtered and solvent removed. Crude was subjected to flash column chromatography (Hexane/EA : 80/20) to yield **S5** (907 mg, 2.45 mmol, 87%) as colourless liquid.

^{1}H NMR (500 MHz, $CDCl_3$) δ = 8.09 – 8.03 (m, 2H, H_h), 7.43 – 7.37 (m, 2H, H_i), 4.60 – 4.57 (m, 2H, H_e), 4.55 – 4.51 (m, 2H, H_d), 4.42 (s, 2H, H_k), 1.94 (s, 6H, H_a).
^{13}C NMR (126 MHz, $CDCl_3$) δ = 171.69 (C_c), 165.96 (C_f), 140.89 (C_j), 130.42 (C_h), 129.76 (C_g), 128.13 (C_i), 63.66 (C_d), 62.50 (C_e), 55.50 (C_b), 54.43 (C_k), 30.83 (C_a).
MS-ESI(+): m/z = 392.02 $[M+Na]^+$
HRMS-ESI(+): calculated for $[M+Na]^+$: 392.0216; found: 392.0213.

3.2 Synthetic Route to S6-S11.

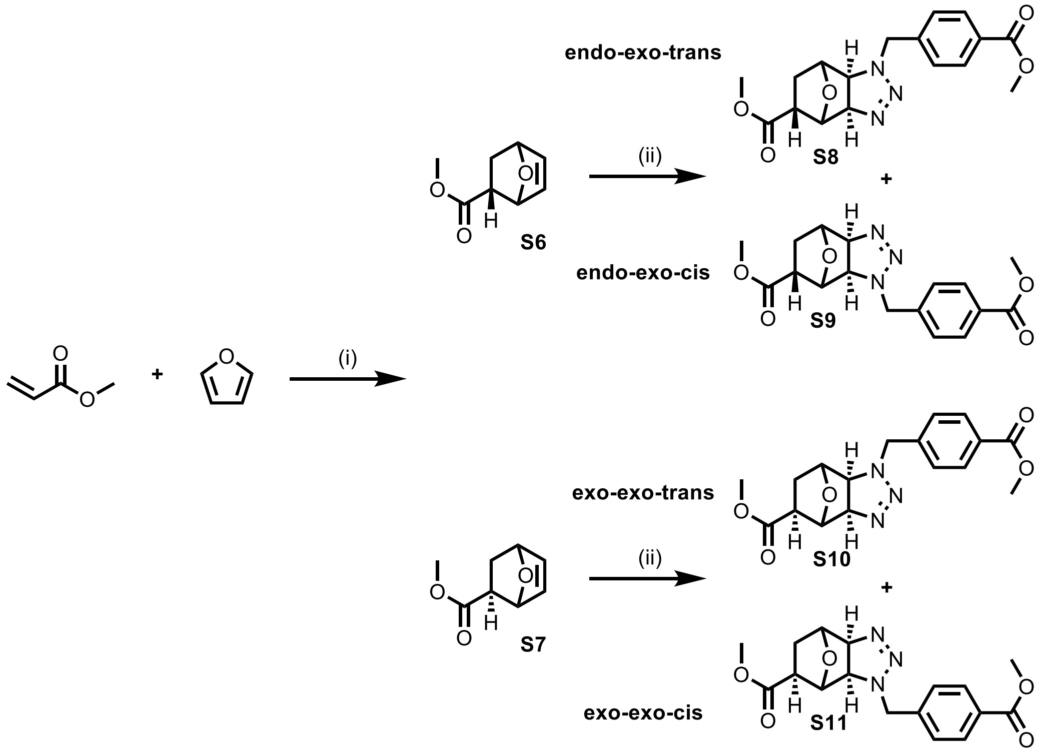

Scheme S2. Synthetic route to **S6-S11.** Conditions: (i) BF_3 Et_2O, 47%; (ii) **S3**, toluene, 50°C 3 days, 36-60%.

3.2.1 Synthesis of S6 and S7

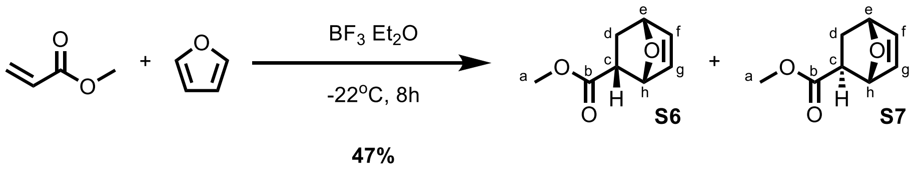

Freshly distilled furan (0.27 ml, 3.67 mmol, 1 equiv.) is added to a nitrogen flushed microwave vial (20 mL) containing methyl acrylate (1 mL, 11.02 mmol, 3 equiv.), which was filtered through basic alumina. The mixture is shaken well to mix the contents and subsequently cooled to -30°C before BF_3 Et_2O (0.10 mL, 0.8 mmol, 0.22 equiv.) is added in one portion. The reaction is then left in the freezer (-22°C) for 8 hours. Next, slightly yellow solution is warmed up to room temperature and diluted with deionised water (30 mL). The aqueous phase is then extracted with diethyl ether (30 mL) and phases separated. Organic phase was dried over anhydrous magnesium sulphate, filtered and concentrated. Crude was subjected to flash column chromatography (PE/EA : 80/20) to yield to yield **S6** (205 mg, 1.32 mmol, 36%) and **S7** (62 mg, 0.4 mmol, 11%).

S6 (*endo*-):
^{1}H NMR (500 MHz, $CDCl_3$) δ = 6.44 (dd, J = 5.8, 1.7 Hz, 1H, H_f), 6.23 (dd, J = 5.8, 1.7 Hz, 1H, H_g), 5.17 (m, 1H, H_h), 5.02 (m, 1H, H_e), 3.64 (s, 3H, H_a), 3.12 (m, 1H, H_c), 2.11 (ddd, J = 11.5, 9.3, 4.7 Hz, 1H, H_{d1}), 1.62 – 1.56 (m, 1H, H_{d2}).
^{13}C NMR (126 MHz, $CDCl_3$) δ = 172.83 (C_b), 137.24 (C_g), 132.74 (C_h), 79.19 (C_e), 78.91 (C_h), 51.93 (C_a), 42.85 (C_c), 28.68 (C_d).

S7 (*exo-*):
^{1}H NMR (500 MHz, CDCl$_3$) δ = 6.39 (dd, J = 5.8, 1.6 Hz, 1H, H_f), 6.35 (dd, J = 5.8, 1.6 Hz, 1H, H_g), 5.19 (m, 1H, H_h), 5.08 (m, 1H, H_e), 3.73 (s, 3H, H_a), 2.44 (dd, J = 8.5, 3.9 Hz, 1H, H_c), 2.21 – 2.13 (dt, J = 11.6, 4.3, Hz, 1H, H_{d1}), 1.62 – 1.49 (m, 1H, H_{d2}).
^{13}C NMR (126 MHz, CDCl$_3$) δ = 174.24 (C_b), 137.08 (C_g), 134.69 (C_f), 80.94 (C_e), 78.02 (C_h), 52.18 (C_a), 42.77 (C_c), 29.11 (C_d).

MS-ESI(+): m/z = 177.05 [M+Na]$^+$
HRMS-ESI(+): calculated for [M+Na]$^+$: 177.0522; found: 177.0518.

3.2.2 Synthesis of S8-S11

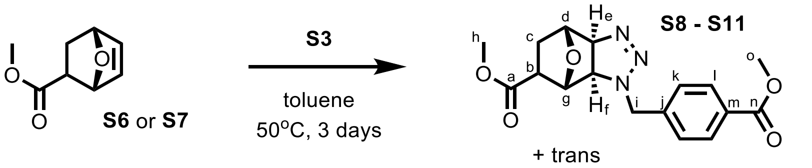

S6 or **S7** (1 equiv.) was added in toluene into a microwave vial (5 mL) containing **S3** (1.2 equiv.) in toluene (0.2 M). Next, the mixture was heated to 50°C and left for 3 days. Upon reaction completion, the solvent was removed and crude purified by preparative TLC (PE/EA : 50/50, 3 elutions) to yield two isomers: *-exo-trans* and *-exo-cis* in approximate ratio of 1 to 1 and isolated yields between 36 and 60%. All reactions were carried out on 0.1 mmol scale. Configurations of the products was confirmed using 2D NMR experiments as well as single crystal XRD (see section 8).

S8 (*endo-exo-trans*):
^{1}H NMR (500 MHz, CDCl$_3$) δ = 8.07 – 8.01 (m, 2H, H_l), 7.40 – 7.35 (m, 2H, H_k), 4.97 (d, J = 15.6 Hz, 1H, H_{i1}), 4.97 (d, J = 5.6 Hz, 1H, H_g), 4.85 (d, J = 15.6 Hz, 1H, H_{i2}), 4.78 (d, J = 9.3 Hz, 1H, H_f), 4.35 (d, J = 5.7 Hz, 1H, H_d), 3.93 (s, 3H, H_o), 3.73 (s, 3H, H_h), 3.45 (d, J = 9.3 Hz, 1H, H_e), 3.04 (dt, J = 11.0, 5.3 Hz, 1H, H_b), 1.89 (ddd, J = 12.9, 11.3, 5.7 Hz, 1H, H_{c1}), 1.77 (dd, J = 12.9, 5.1 Hz, 1H, H_{c2}).
^{13}C NMR (126 MHz, CDCl$_3$) δ = 172.10 (C_a), 166.82 (C_n), 141.53 (C_j), 130.33 (C_l), 130.11 (C_m), 128.12 (C_k), 83.55 (C_f), 81.09 (C_g), 80.82 (C_d), 62.52 (C_e), 52.55 (C_h), 52.38 (C_i + C_o), 45.06 (C_b), 29.53 (C_c).

S9 (*endo-exo-cis*):
^{1}H NMR (500 MHz, CDCl$_3$) δ = 8.04 (d, J = 8.0 Hz, 2H, H_l), 7.37 (d, J = 8.0 Hz, 2H, H_k), 4.91 (d, J = 15.1 Hz, 1H, H_{i1}), 4.88 (d, J = 5.6 Hz, 1H, H_d), 4.83 (d, J = 15.2 Hz, 1H, H_{i2}), 4.73 (d, J = 9.3 Hz, 1H, H_e), 4.38 (d, J = 5.6 Hz, 1H, H_g), 3.92 (s, 3H, H_o), 3.56 (s, 3H, H_h), 3.41 (d, J = 9.3 Hz, 1H, H_f), 2.95 (dt, J = 11.0, 5.3 Hz, 1H, H_b), 2.01 (td, J = 12.1, 5.6 Hz, 1H, H_{c1}), 1.93 (dd, J = 12.8, 4.9 Hz, 1H, H_{c2}).
^{13}C NMR (126 MHz, CDCl$_3$) δ = 171.87 (C_a), 166.79 (C_n), 141.16 (C_j), 130.26 (C_l), 130.13 (C_m), 128.50 (C_k), 86.81 (C_e), 81.94 (C_d), 79.73 (C_g), 59.69 (C_f), 52.68 (C_i), 52.39 (C_o), 52.27 (C_h), 44.65 (C_b), 30.27 (C_c).

S10 (*exo-exo-trans*):
^{1}H NMR (500 MHz, CDCl$_3$) δ = 8.01 (d, J = 8.1 Hz, 2H, H_l), 7.37 (d, J = 8.0 Hz, 2H, H_k), 5.10 (s, 1H, H_g), 4.98 (d, J = 15.7 Hz, 1H, H_{i1}), 4.83 (d, J = 15.8 Hz, 1H, H_{i2}), 4.77 (d, J = 9.3 Hz, 1H, H_f), 4.40 (d, J = 5.6 Hz, 1H, H_d), 3.92 (s, 3H, H_o), 3.71 (s, 3H, H_h), 3.41 (d, J = 9.3 Hz, 1H, H_e), 2.67 (dd, J = 9.1, 4.5 Hz, 1H, H_b), 2.11 (dt, J = 13.1, 5.1 Hz, 1H, H_{c1}), 1.61 (dd, J = 13.1, 9.1 Hz, 1H, H_{c2}).
^{13}C NMR (126 MHz, CDCl$_3$) δ = 172.63 (C_a), 166.78 (C_n), 141.52 (C_j), 130.29 (C_l), 130.08 (C_m), 128.05 (C_k), 86.43 (C_f), 82.73 (C_g), 79.08 (C_d), 62.56 (C_e), 52.63 (C_h), 52.36 (C_o), 52.29 (C_i), 44.65 (C_b), 29.99 (C_c).

S11 (*endo-exo-cis*):

^{1}H NMR (500 MHz, CDCl$_3$) δ = 8.06 – 8.00 (m, 2H, H_l), 7.36 (d, J = 8.1 Hz, 2H, H_k), 5.10 (d, J = 15.8 Hz, 1H, H_{i1}), 4.91 (d, J = 5.5 Hz, 1H, H_d), 4.73 (d, J = 25.8 Hz, 1H, H_{i2}), 4.71 (d, J = 9.6 Hz, 1H, H_e), 4.66 (s, 1H, H_g), 3.92 (s, 3H, H_o), 3.67 (s, 3H, H_h), 3.44 (d, J = 9.3 Hz, 1H, H_f), 2.49 (dd, J = 9.1, 5.1 Hz, 1H, H_b), 2.17 (dt, J = 12.9, 5.4 Hz, 1H, H_{c1}), 1.82 (dd, J = 12.9, 9.1 Hz, 1H, H_{c2}).

^{13}C NMR (126 MHz, CDCl$_3$) δ = 172.59 (C_a), 166.79 (C_n), 141.47 (C_j), 130.31 (C_l), 130.06 (C_m), 128.02 (C_k), 86.68 (C_e), 81.24 (C_g), 80.61 (C_d), 62.09 (C_f), 52.57 (C_h), 52.34 (C_o), 52.04 (C_i), 43.96 (C_b), 31.15 (C_c).

MS-ESI(+): m/z = 368.12 [M+Na]$^+$

HRMS-ESI(+): calculated for [M+Na]$^+$: 368.1217; found: 368.1211.

3.3 Synthetic Route to S12-S15.

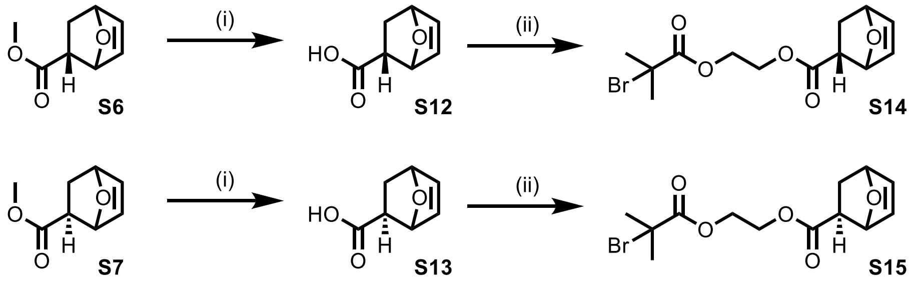

Scheme S3. Synthetic route to **S12-S15.** Conditions: (i) LiOH x H_2O, THF/MeOH/water, 24h, RT, assumed quant.; (ii) EDCl, DMAP, **S1**, DCM, 24h, 54-87%.

3.3.1 Synthesis of S12 and S13

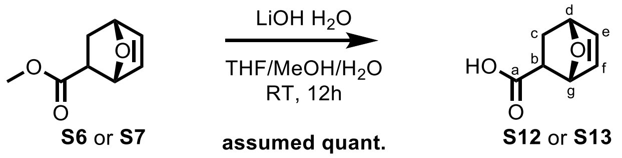

S6 or **S7** (500 mg, 3.24 mmol, 1 equiv.) was dissolved in THF (5 mL) and added into a round bottom flask (25 mL) containing LiOH monohydrate (680 mg, 16.22 mmol, 5 equiv.) in water and methanol mixture (1 and 5 mL, respectively) at room temperature. Setup was closed and the mixture stirred vigorously at RT for 24 hours. Next, mixture was neutralised with acetic acid (3 mL) and diluted with HCl (1 M, 30 mL), aqueous layer extracted with ethyl acetate (3 x 30 mL) and phases separated. Organic layer was then dried over anhydrous magnesium sulphate, filtered, solvent removed and crude dried on high vacuum for 2-3 days. Crude white solids of **S12** and **S13** were used without any purification in the next steps.

3.3.2 Synthesis of S14 and S15

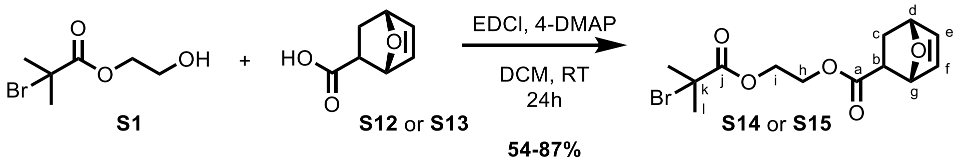

S12 or **S13** (40 mg, 285 µmol, 1 equiv.) was added along **S1** (80 mg, 371 µmol, 1.3 equiv.) in DCM (20 mL) into a microwave vial (5 mL) containing EDC hydrochloride (75 mg, 371 µmol, 1.3 equiv.) and 4-DMAP (45 mg, 371 µmol, 1.3 equiv.) under nitrogen atmosphere. Reaction was left to stir at room temperature for 24 hours before being diluted with deionised water (10 ml). Next, aqueous layer was extracted with ethyl acetate (3 x 10 mL) and phases separated. Organic layer was then dried

over anhydrous magnesium sulphate, filtered and solvent removed. Crude was subjected to separation on preparative TLC (PE/EA : 90/10 to 70/30 in 3 elutions) plate to yield **S14** (82.7 mg, 248 µmol, 87%) or **S15** (51.5 mg, 154 µmol, 54%) as colourless oils.

S14 (*endo*-):

^{1}H NMR (500 MHz, CDCl$_3$) δ = 6.44 (dd, J = 5.8, 1.7 Hz, 1H, H_e), 6.27 (dd, J = 5.8, 1.6 Hz, 1H, H_f), 5.18 (dt, J = 4.8, 1.1 Hz, 1H, H_g), 5.03 (dd, J = 4.8, 1.7 Hz, 1H, H_d), 4.44 – 4.22 (m, 4H, H_h + H_i), 3.14 (ddd, J = 8.9, 4.8, 3.8 Hz, 1H, H_b), 2.11 (ddd, J = 11.5, 9.2, 4.7 Hz, 1H, H_{c1}), 1.97 – 1.91 (m, 6H, H_l), 1.59 (dd, J = 11.5, 3.7 Hz, 1H, H_{c2}).

^{13}C NMR (126 MHz, CDCl$_3$) δ = 172.02 (C_a), 171.58 (C_j), 137.33 (C_e), 132.76 (C_f), 79.19 (C_d), 78.87 (C_g), 63.61 (C_h or C_i), 62.06 (C_h or C_i), 55.39 (C_k), 42.91 (C_b), 30.81 (C_l), 28.64 (C_c).

S15 (*exo*-):

^{1}H NMR (500 MHz, CDCl$_3$) δ = 6.40 (dd, J = 5.8, 1.6 Hz, 1H, H_e), 6.35 (dd, J = 5.8, 1.7 Hz, 1H, H_f), 5.20 (d, J = 1.7 Hz, 1H, H_g), 5.07 (dd, J = 4.8, 1.7 Hz, 1H, H_d), 4.44 – 4.33 (m, 4H, H_h + H_i), 2.46 (dd, J = 8.5, 3.9 Hz, 1H, H_b), 2.17 (dt, J = 11.7, 4.2 Hz, 1H, H_{c1}), 1.94 (s, 6H, H_l), 1.56 (dd, J = 11.6, 8.5 Hz, 1H, H_{c2}).

^{13}C NMR (126 MHz, CDCl$_3$) δ = 173.61 (C_a), 171.62 (C_j), 137.31 (C_e), 134.78 (C_f), 81.04 (C_g), 78.15 (C_d), 63.66 (C_h), 62.30 (C_i), 55.60 (C_k), 42.93 (C_b), 30.81 (C_l), 29.21 (C_c).

MS-ESI(+): m/z = 177.05 [M+Na]$^+$

HRMS-ESI(+): calculated for [M+Na]$^+$: 177.0522; found: 177.0518.

3.4 Synthetic Route to 1-4.

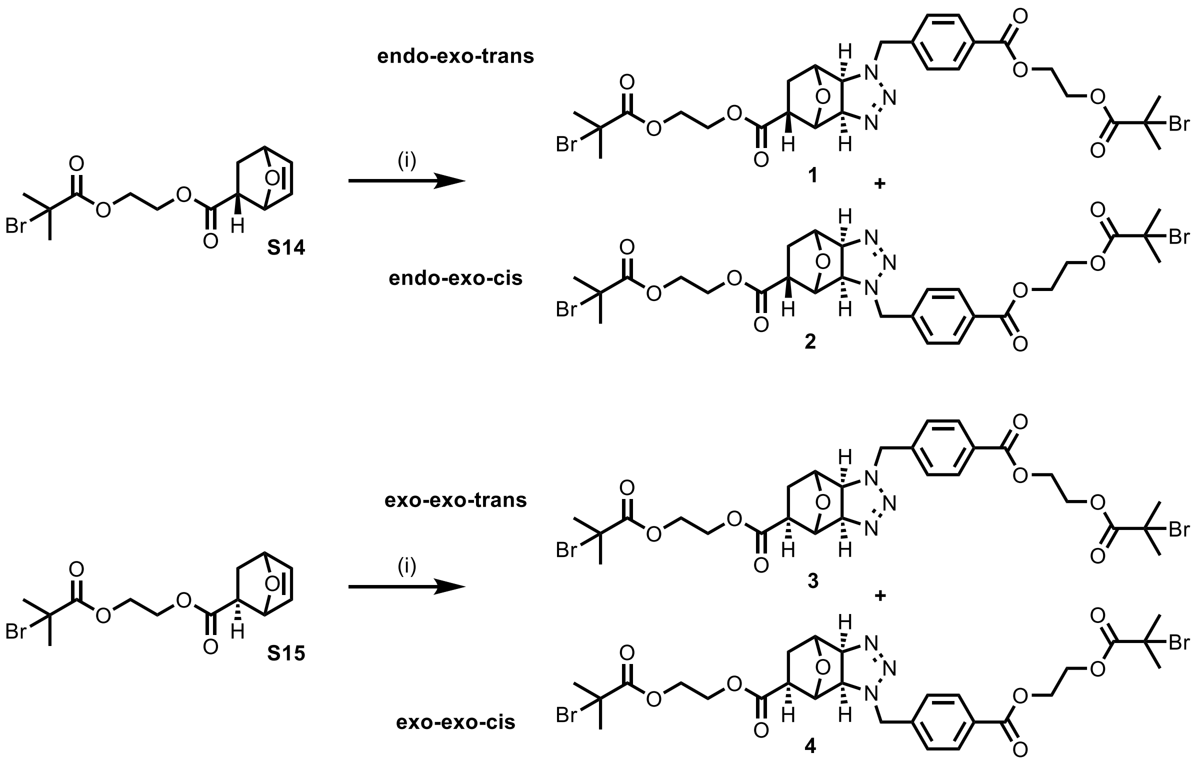

Scheme S4. Synthetic route to **1-4.** Conditions: (i) **S5**, toluene, 50°C, 3 days, 19-34%.

3.4.1 Synthesis of 1-4

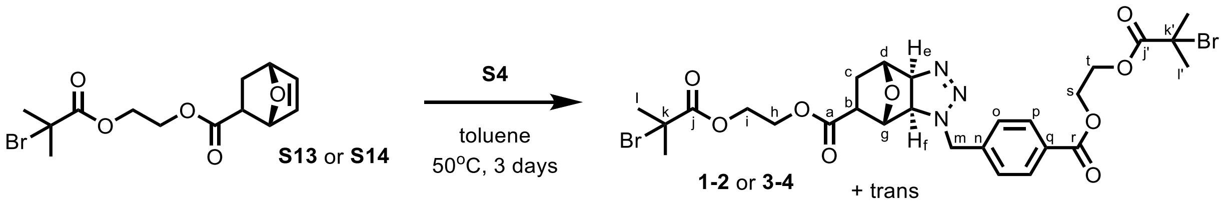

S14 (30 mg, 90 µmol, 1 equiv.) was added in toluene into a microwave vial (5 mL) containing **S5** (40 mg, 108 µmol, 1.2 equiv.) in toluene (0.5 mL). Next, the mixture was heated to 50°C and left for 3 days. Upon reaction completion, the solvent was removed and crude purified by preparative TLC (PE/EA : 70/30 to 50/50 in 3 elutions, then DCM/EA : 90/10) to yield two isomers: *endo-exo-trans* (**1**, 21.4 mg, 30 µmol, 34%) and *endo-exo-cis* (**2**, 16.3 mg, 23 µmol, 26%) as light yellow oils.

The same procedure, as well as quantities, were employed starting with **S15** to yield two isomers: *exo-exo-trans* (**3**, 15 mg, 21.6 µmol, 24%) and *exo-exo-cis* (**4**, 12 mg, 17.1 µmol, 19%).

1 (*endo-exo-trans*):
^{1}H NMR (500 MHz, acetone-d$_6$) δ = 8.05 – 8.02 (m, 2H, H_p), 7.51 – 7.46 (m, 2H, H_o), 4.95 (dd, J = 48.1, 15.6 Hz, 2H, H_m), 4.83 (s, 1H, H_g), 4.81 (d, J = 4.1 Hz, 1H, H_f), 4.63 – 4.58 (m, 2H, H_s), 4.58 – 4.53 (m, 2H, H_t), 4.49 – 4.36 (m, 5H, H_d + H_h + H_i), 3.59 (d, J = 9.3 Hz, 1H, H_e), 3.06 (dt, J = 11.1, 5.4 Hz, 1H, H_b), 1.92 (d, J = 6.3 Hz, 12H, H_l + $H_{l'}$), 1.88 – 1.84 (m, 1H, H_{c1}), 1.77 (dd, J = 12.8, 5.3 Hz, 1H, H_{c2}).
^{13}C NMR (126 MHz, acetone-d$_6$) δ = 172.04 (C_a), 171.94 (C_j and $C_{j'}$), 166.48 (C_r), 144.12 (C_n), 130.79 (C_p), 130.34 (C_q), 129.19 (C_o), 84.49 (C_f), 81.77 (C_d), 81.64 (C_g), 64.60 (C_t), 64.50 (C_i), 63.45 (C_h), 63.33 (C_s), 63.29 (C_e), 57.17 (C_k or $C_{k'}$), 57.13 (C_k or $C_{k'}$), 52.31 (C_m), 45.94 (C_b), 31.04 (C_l and $C_{l'}$), 30.05 (C_c).

2 (*endo-exo-cis*):
^{1}H NMR (500 MHz, acetone-d$_6$) δ = 8.08 – 8.02 (m, 2H, H_p), 7.53 – 7.48 (m, 2H, H_o), 4.93 (s, 2H, H_m), 4.76 (d, J = 2.0 Hz, 1H, H_d), 4.74 (d, J = 6.1 Hz, 1H, H_e), 4.62 – 4.59 (m, 2H, H_s), 4.58 – 4.54 (m, 2H, H_t), 4.47 (d, J = 5.6 Hz, 1H, H_g), 4.40 – 4.25 (m, 4H, H_h + H_i), 3.66 (d, J = 9.2 Hz, 1H, H_f), 2.99 (dt, J = 11.0, 5.6 Hz, 1H, H_b), 2.00 – 1.94 (m, 2H, H_c), 1.94 – 1.88 (m, 12H, H_l + $H_{l'}$).
^{13}C NMR (126 MHz, acetone-d$_6$) δ = 171.99 (C_j or $C_{j'}$ or C_a), 171.96 (C_j or $C_{j'}$ or C_a), 171.95 (C_j or $C_{j'}$ or C_a), 166.46 (C_r), 143.92 (C_n), 130.83 (C_p), 130.43 (C_q), 129.37 (C_o), 87.97 (C_e), 82.57 (C_d), 80.67 (C_g), 64.60 (C_t), 64.48 (C_i), 63.34 (C_s), 63.23 (C_h), 60.71 (C_f), 57.18 (C_k or $C_{k'}$), 57.15 (C_k or $C_{k'}$), 52.71 (C_m), 45.55 (C_b), 31.15 (C_c), 31.04 (C_l or $C_{l'}$), 31.04 (C_l or $C_{l'}$).

3 (*exo-exo-trans*):
^{1}H NMR (500 MHz, acetone-d$_6$) δ = 8.05 – 8.01 (m, 2H, H_p), 7.49 – 7.46 (m, 2H, H_o), 5.00 (d, J = 15.8 Hz, 1H, H_{m1}), 4.95 (s, 1H, H_g), 4.92 – 4.86 (m, 2H, H_{m2} + H_f), 4.62 – 4.58 (m, 2H, H_s), 4.57 – 4.54 (m, 2H, H_t), 4.48 (d, J = 5.6 Hz, 1H, H_d), 4.44 – 4.36 (m, 4H, H_h + H_i), 3.66 – 3.62 (m, 1H, H_e), 2.91 (dd, J = 9.1, 4.6 Hz, 1H, H_b), 1.98 (ddd, J = 12.8, 5.7, 4.6 Hz, 1H, H_{c1}), 1.95 – 1.88 (m, 12H, H_l + $H_{l'}$), 1.76 (dd, J = 12.8, 9.1 Hz, 1H, H_{c2}).
^{13}C NMR (126 MHz, acetone-d$_6$) δ = 173.04 (C_a), 171.91 (C_j or $C_{j'}$), 171.89 (C_j or $C_{j'}$), 166.46 (C_r), 144.18 (C_n), 130.75 (C_p), 130.29 (C_q), 129.13 (C_r), 87.40 (C_f), 83.43 (C_g), 80.30 (C_d), 64.58 (C_t), 64.49 (C_i), 63.32 (C_e or C_h), 63.27 (C_e or C_h), 63.15 (C_s), 57.15 (C_k and $C_{k'}$), 52.30 (C_m), 45.30 (C_b), 31.01 (C_l and $C_{l'}$), 30.53 (C_c).

4 (*endo-exo-cis*):
^{1}H NMR (500 MHz, acetone-d$_6$) δ = 8.06 – 8.02 (m, 2H, H_p), 7.50 – 7.46 (m, 2H, H_o), 5.04 (d, J = 15.9 Hz, 1H, H_{m1}), 4.87 (d, J = 15.9 Hz, 1H, H_{m2}), 4.80 (d, J = 9.2 Hz, 1H, H_e), 4.76 (d, J = 5.5 Hz, 1H, H_d), 4.69 (s, 1H, H_g), 4.62 – 4.59 (m, 2H, H_s), 4.57 – 4.55 (m, 2H, H_t), 4.40 – 4.31 (m, 4H, H_i + H_h), 3.73 (d, J = 9.2

Hz, 1H, H_f), 2.76 (dd, J = 9.1, 5.1 Hz, 1H, H_b), 2.09 – 2.07 (m, 1H, H_{c1}), 1.97 – 1.94 (m, 1H, H_{c2}), 1.93 (s, 6H, H_l and/or $H_{l'}$), 1.90 (s, 6H, H_l and/or $H_{l'}$).
^{13}C NMR (126 MHz, acetone-d_6) δ = 173.08 (C_a), 171.92 (C_j or $C_{j'}$), 171.87 (C_j or $C_{j'}$), 166.47 (C_r), 144.14 (C_n), 130.79 (C_p), 130.31 (C_q), 129.09 (C_o), 87.84 (C_e), 82.45 (C_g), 81.25 (C_d), 64.60 (C_t), 64.46 (C_i), 63.33 (C_s), 63.06 (C_h), 62.97 (C_f), 57.23 (C_k or $C_{k'}$), 57.16 (C_k or $C_{k'}$), 52.30 (C_m), 44.50 (C_b), 31.74 (C_c), 31.03 – 30.99 (m, C_l and $C_{l'}$).

MS-ESI(-): m/z = 702.05 $[M-H]^-$
HRMS-ESI(-): calculated for $[M-H]^-$: 700.0500; found: 700.0511.

MS-ESI(+): m/z = 726.04 $[M+Na]^+$
HRMS-ESI(+): calculated for $[M+Na]^+$: 724.0476; found: 724.0495.

4 Synthesis of Polymers:

4.1 Representative procedure: synthesis of P-1

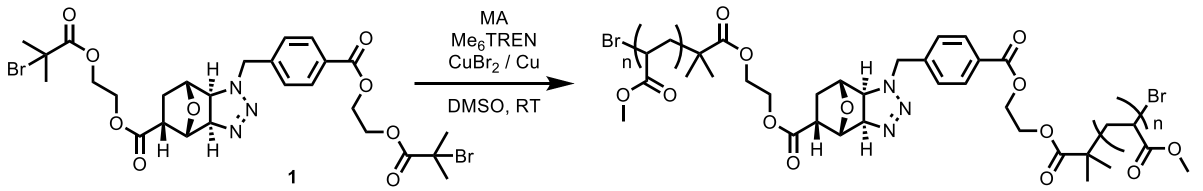

Methyl acrylate was passed through basic alumina and degassed with nitrogen for 10 minutes prior to use. Stock catalyst solution was prepared by dissolving copper (II) bromide (5.6 mg) and Me_6TREN (16µL) in DMSO (1 mL). **1** (5 mg, 7.1 µmol, 1.0 equiv.) was dissolved in DMSO (0.65 mL), methyl acrylate (0.65 mL, 7.11 mmol, 1000 equiv.) and stock solution of the catalyst (60 µL, 0.48 equiv.) were combined in a microwave vial (5 mL). Extra amount of methyl acrylate (1 mL) was added and the resulting mixture degassed. Next, stirrer bar was wrapped with copper wire (2.5 cm) and submerged in concentrated hydrochloric acid for 10 min, then washed with acetone and dried on high vacuum prior to addition into the reaction mixture. Once the stirrer was added, the mixture was sealed and degassed for further 1 minute with nitrogen. After 35 minutes, reaction was stopped by exposing it to the atmosphere. Next, the viscous solution was added onto stirring methanol (800 mL) in a beaker (1 L) to precipitate the polymer, which was then transferred into a vial and dried on high vacuum for 4 days.

4.2 List of polymers

All polymers were analysed using GPC with the obtained results visible in *Table S1* and *Figures S1 – S4*. Polymers **P-S14** and **P-S15** show a high mass shoulder in their GPC chromatograms. This was attributed to a possibility of the small number of chains interacting with double bond within compounds **S14** and **S15**. Regardless of the origin of the high mass shoulder, majority of **P-S14** and **P-S15** consists of the correct species.

Table S1. M_n and *Đ* values for polymers **P-1 – P4**, **P-S5**, **P-S14** and **P-S15** as measured by GPC.

Polymer	M_n / *kDa*	M_w / kDa	*Đ*
**P-1**	72.8	80.5	1.11
**P-2**	75.9	88.1	1.16
**P-3**	67.3	75.8	1.13
**P-4**	79.1	88.1	1.11
**P-S5**	43.5	48.0	1.10
**P-S14**	78.6	97.6	1.24
**P-S15**	98.6	137.0	1.40

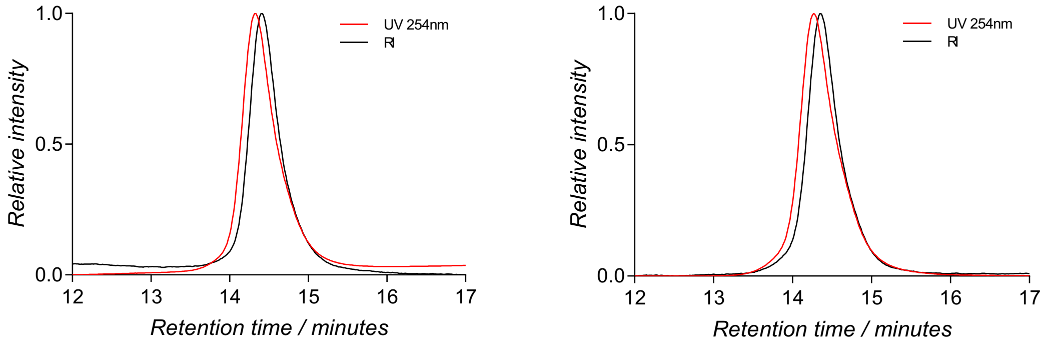

Figure S1. GPC chromatograms of polymers **P-1** and **P-2**, left to right, respectively.

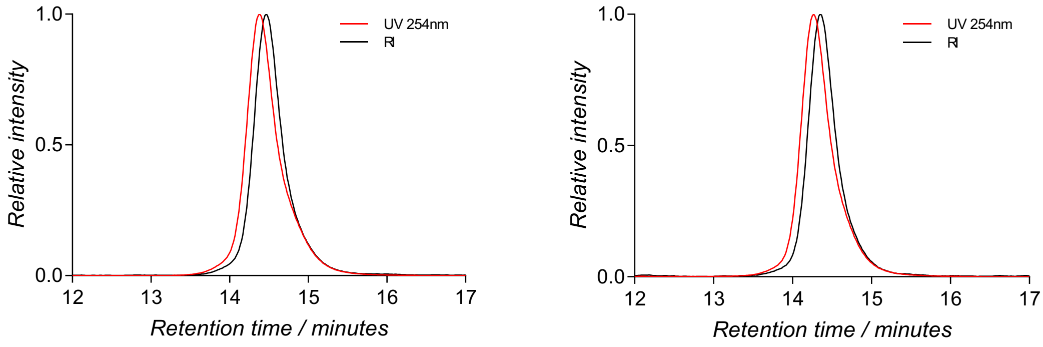

Figure S2. GPC chromatograms of polymers **P-3** and **P-4**, left to right, respectively.

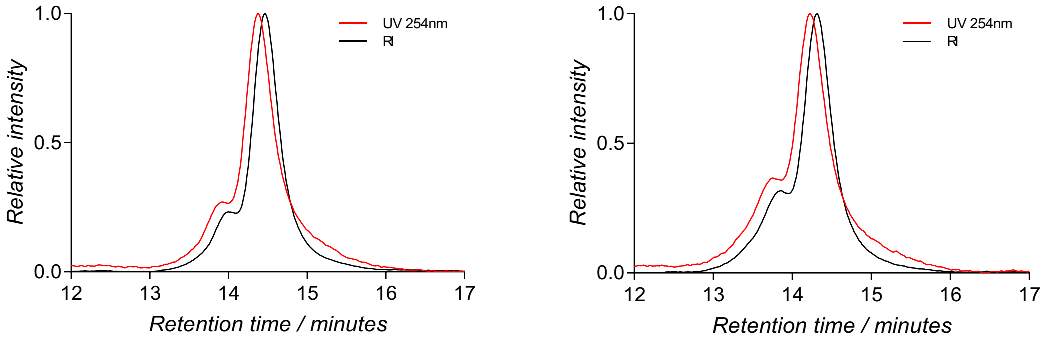

Figure S3. GPC chromatograms of polymers **P-S14** and **P-S15**, left to right, respectively.

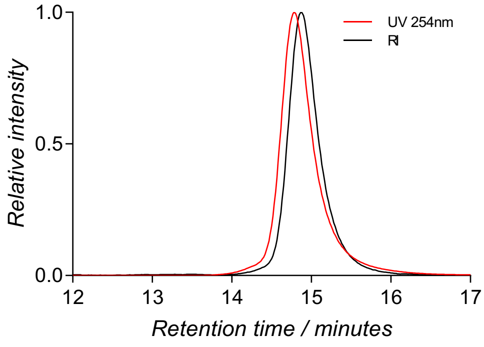

Figure S4. GPC chromatogram of polymer **P-S5**.

5 NMR Spectra

5.1 Spectra of S3

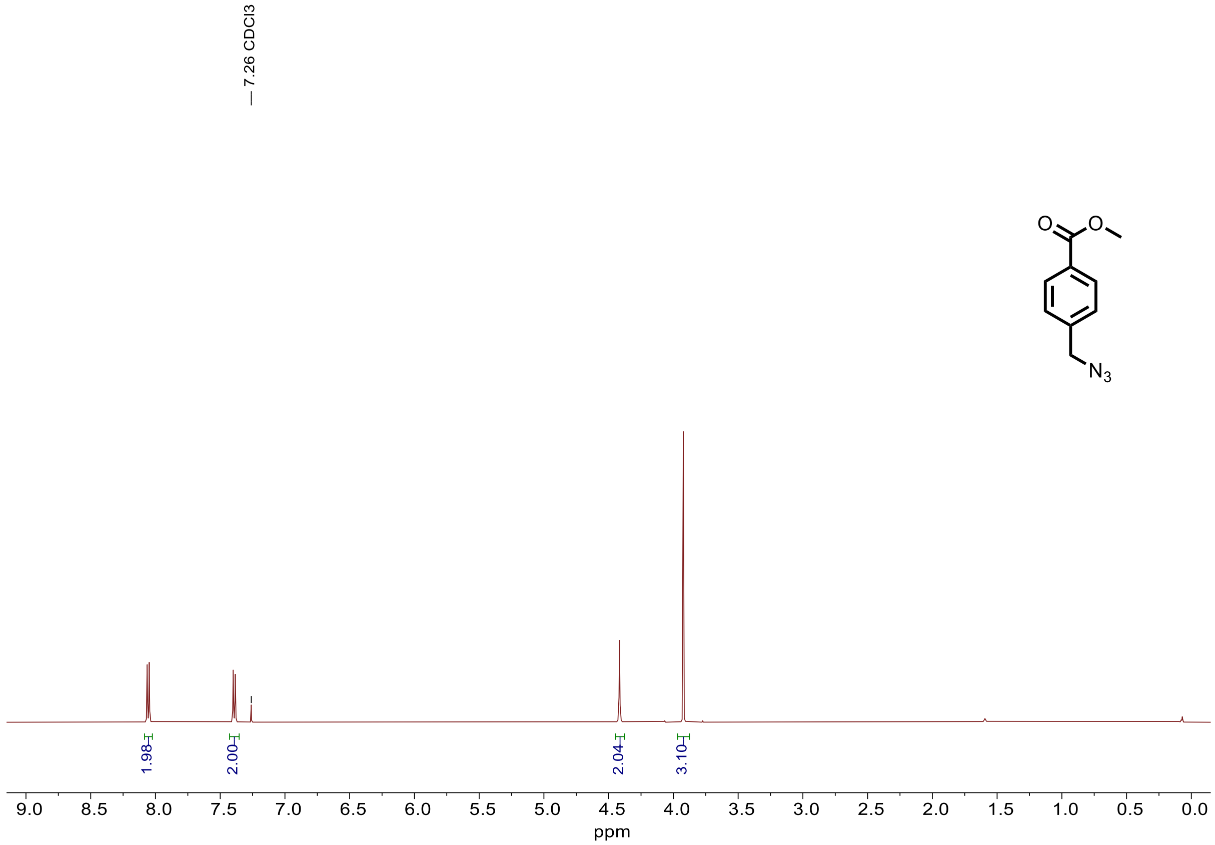

Spectrum S1. ^{1}H NMR (500 MHz, $CDCl_3$, 298 K) of compound **S3**.

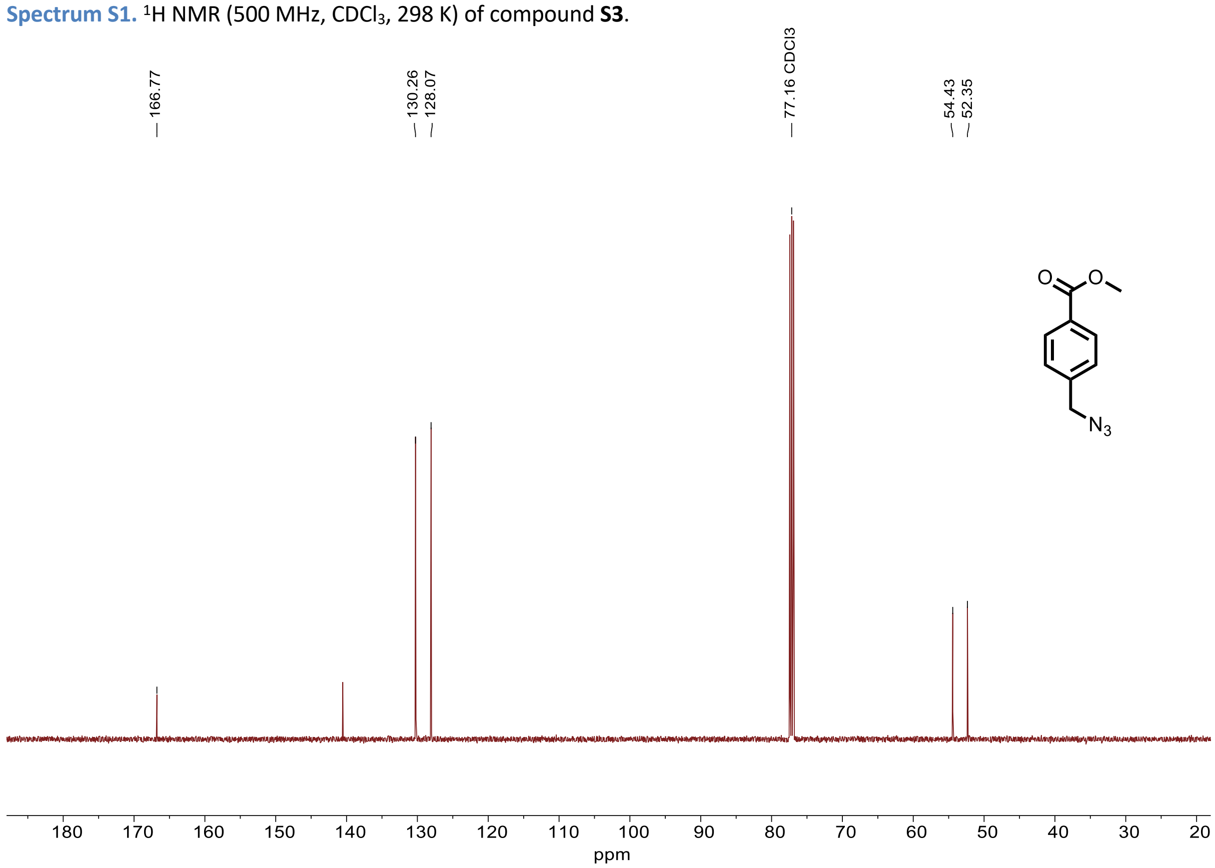

Spectrum S2. ^{13}C NMR (126 MHz, $CDCl_3$, 298 K) of compound **S3**.

5.2 Spectra of S4

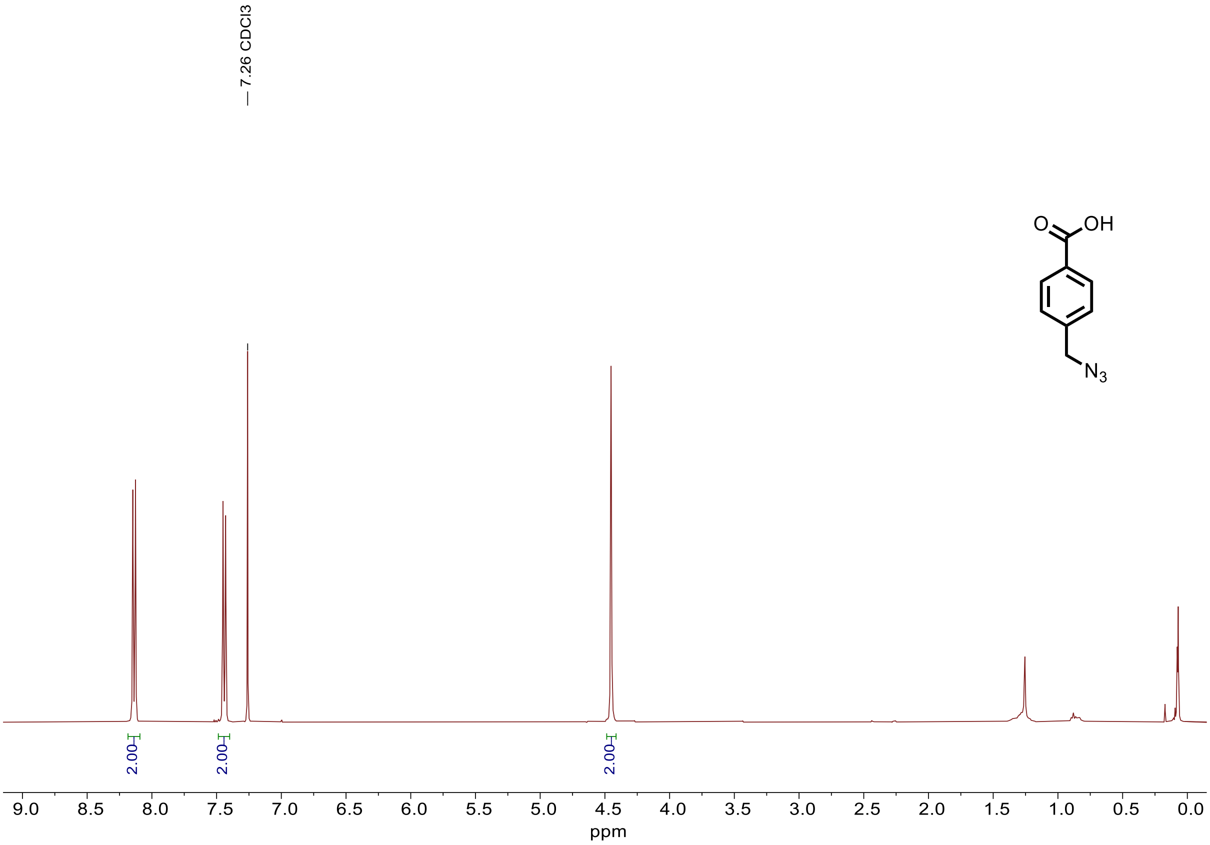

Spectrum S3. ^{1}H NMR (500 MHz, $CDCl_3$, 298 K) of compound **S4**.

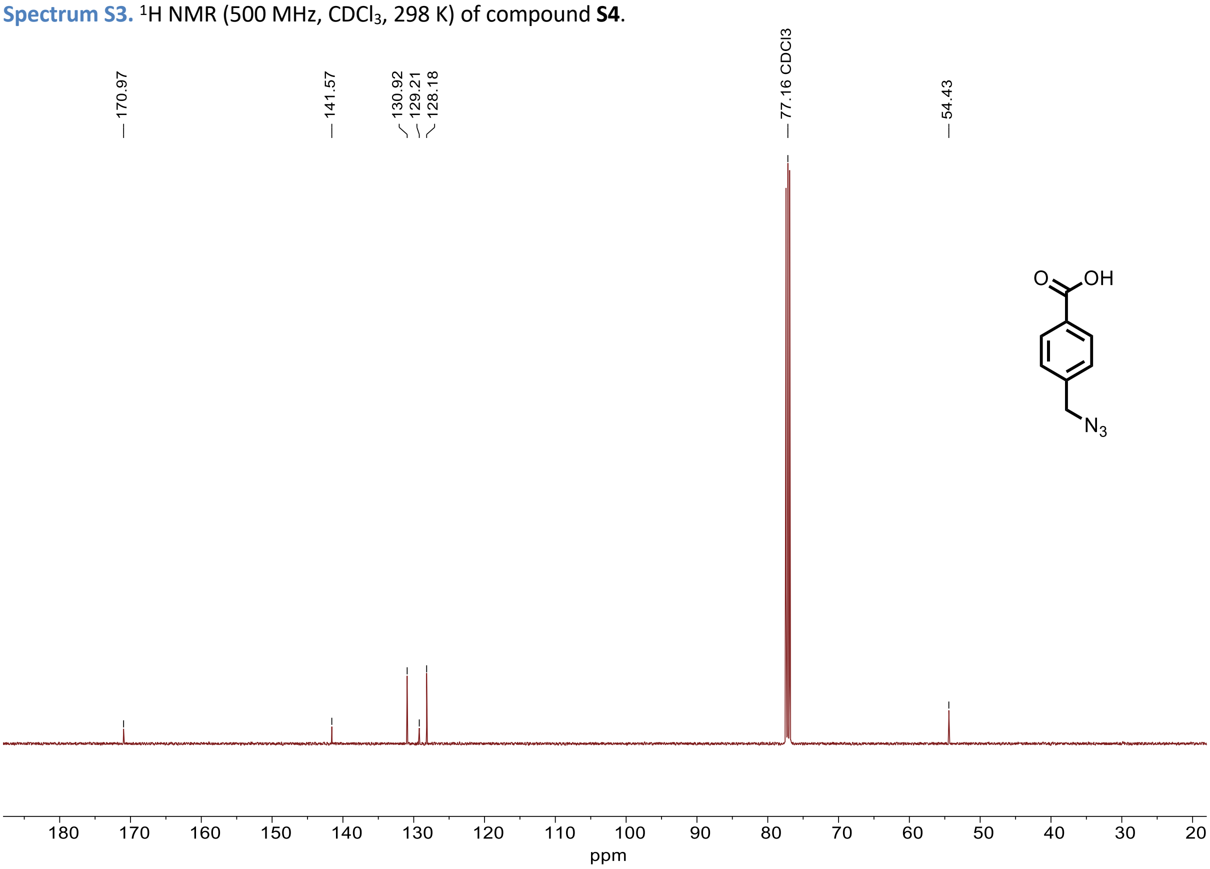

Spectrum S4. ^{13}C NMR (126 MHz, $CDCl_3$, 298 K) of compound **S4**.

5.3 Spectra of S5

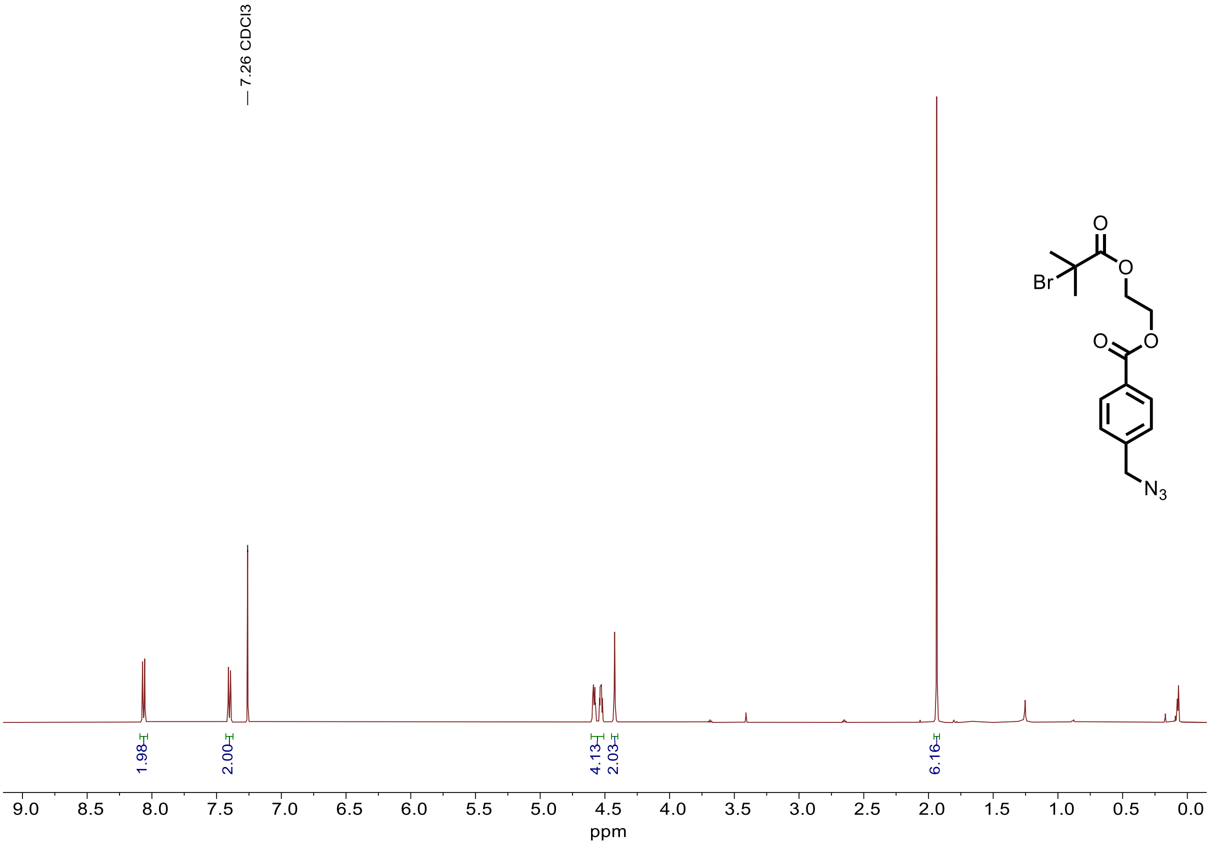

Spectrum S5. ^1H NMR (500 MHz, $CDCl_3$, 298 K) of compound **S5**.

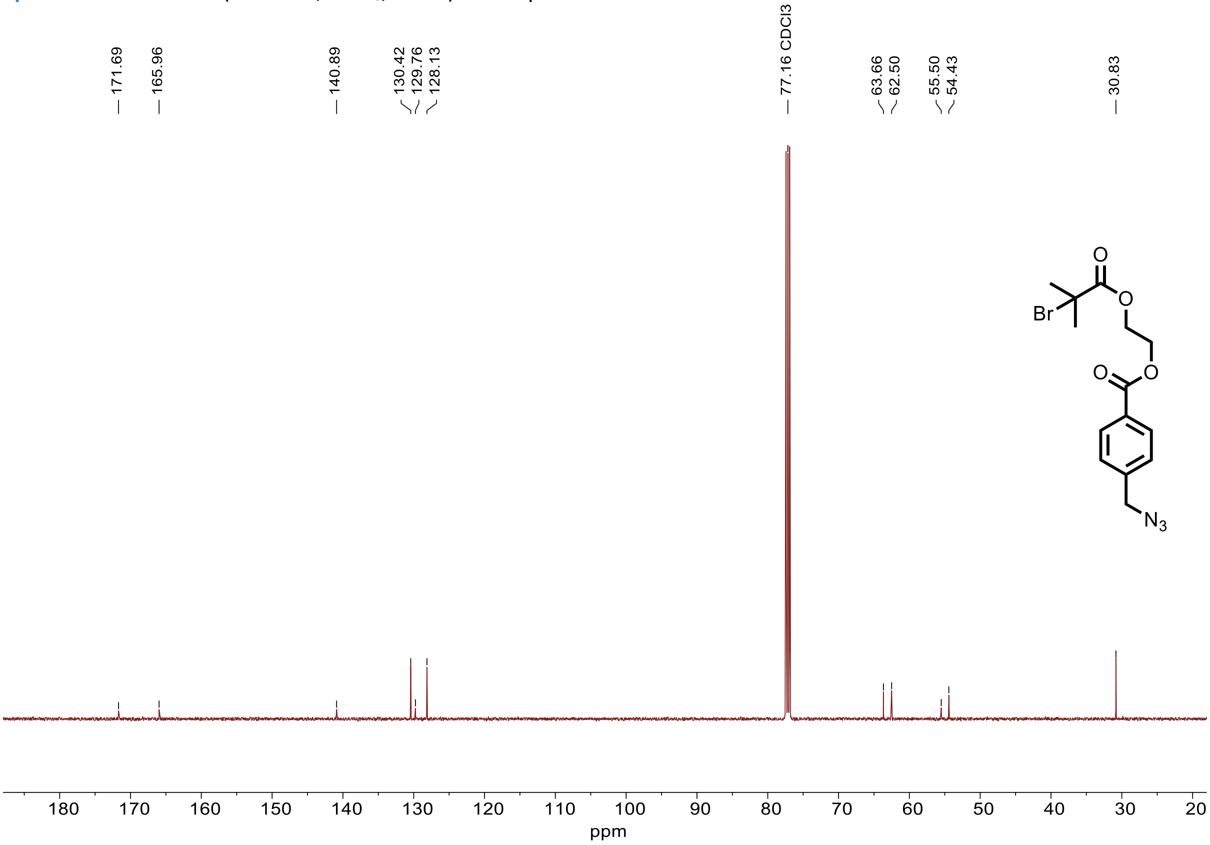

Spectrum S6. ^{13}C NMR (126 MHz, $CDCl_3$, 298 K) of compound **S5**.

5.4 Spectra of S6

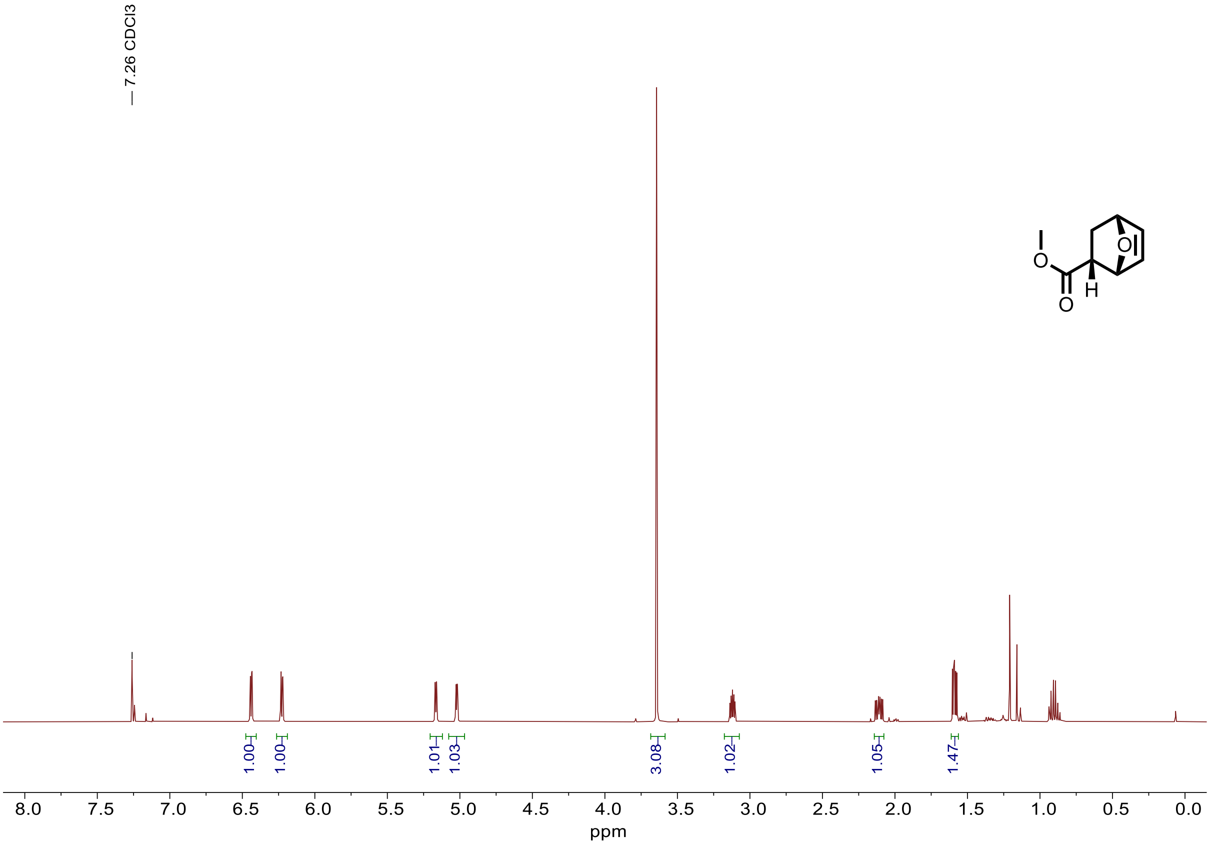

Spectrum S7. ^{1}H NMR (500 MHz, $CDCl_3$, 298 K) of compound **S6**.

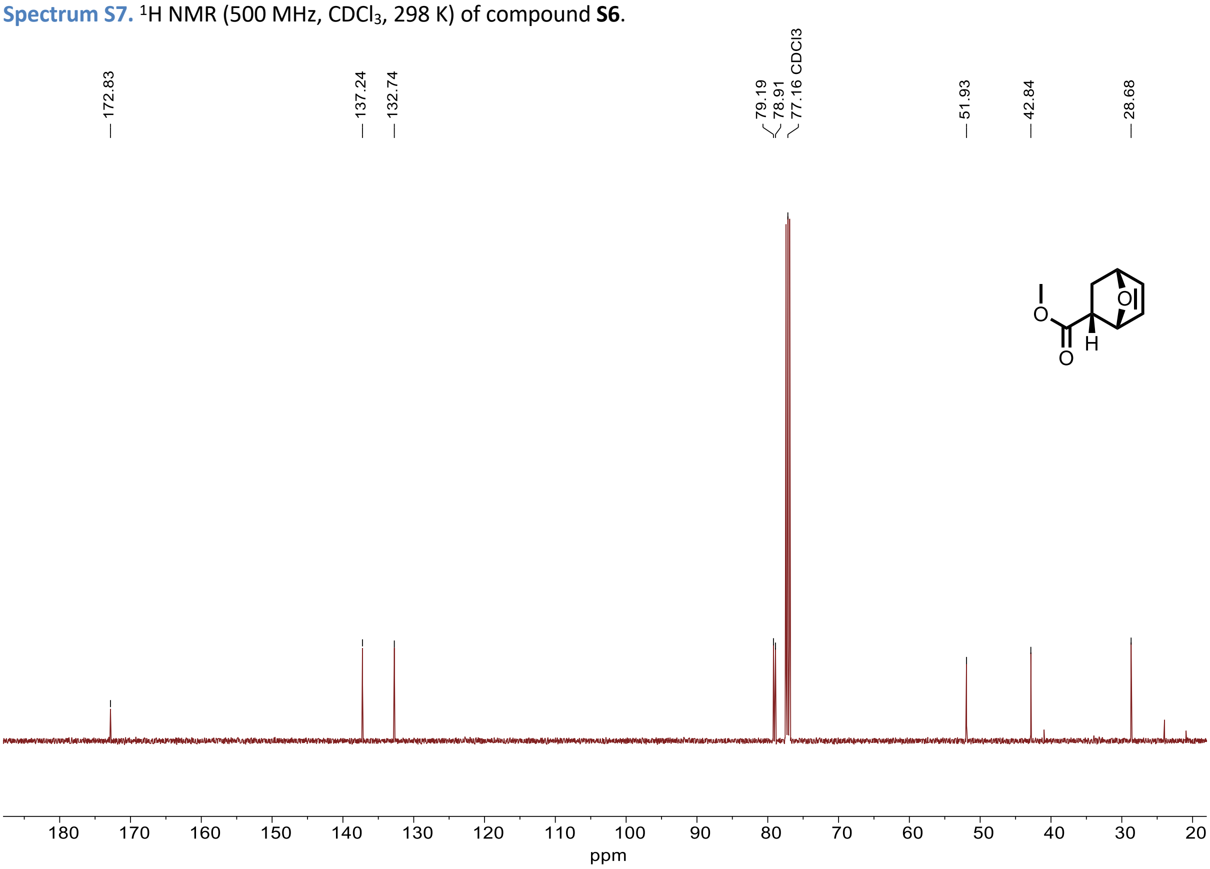

Spectrum S8. ^{13}C NMR (126 MHz, $CDCl_3$, 298 K) of compound **S6**.

5.5 Spectra of S7

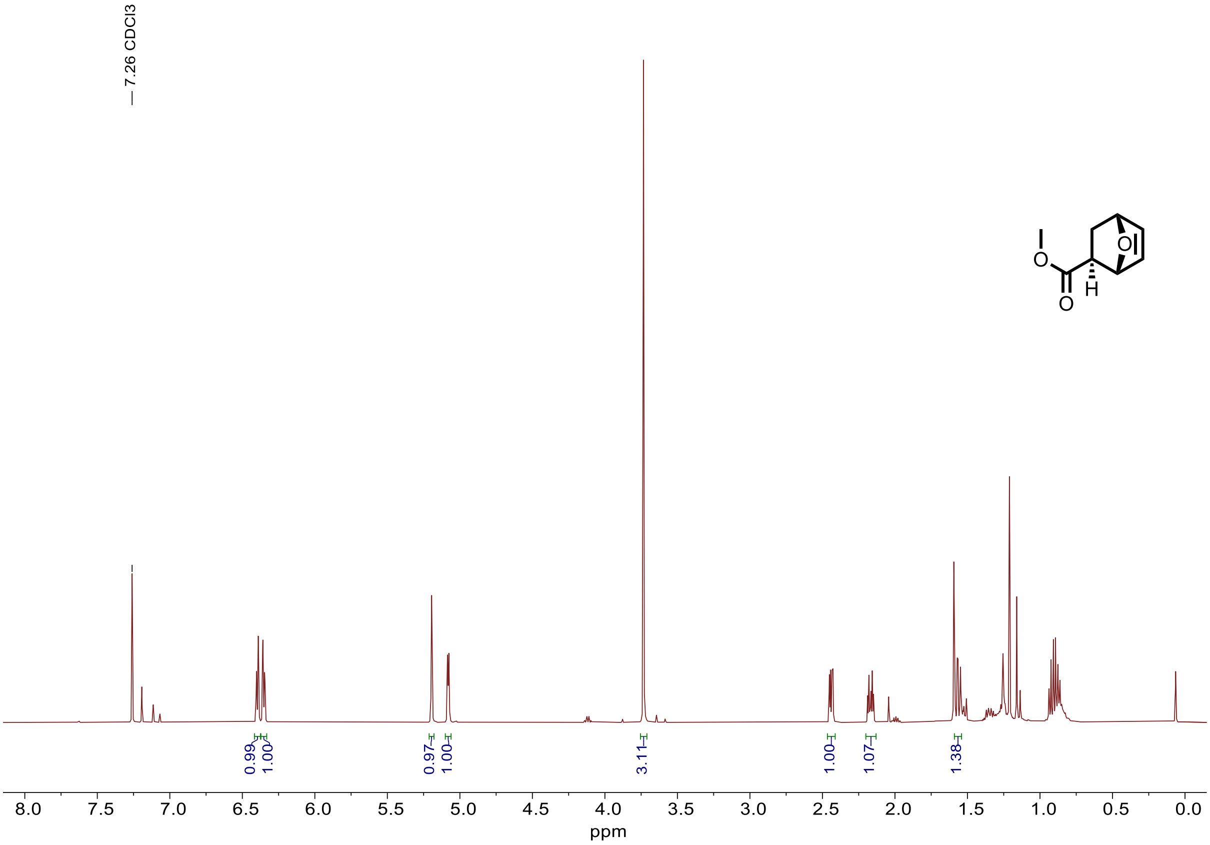

Spectrum S9. ^{1}H NMR (500 MHz, $CDCl_3$, 298 K) of compound **S7**.

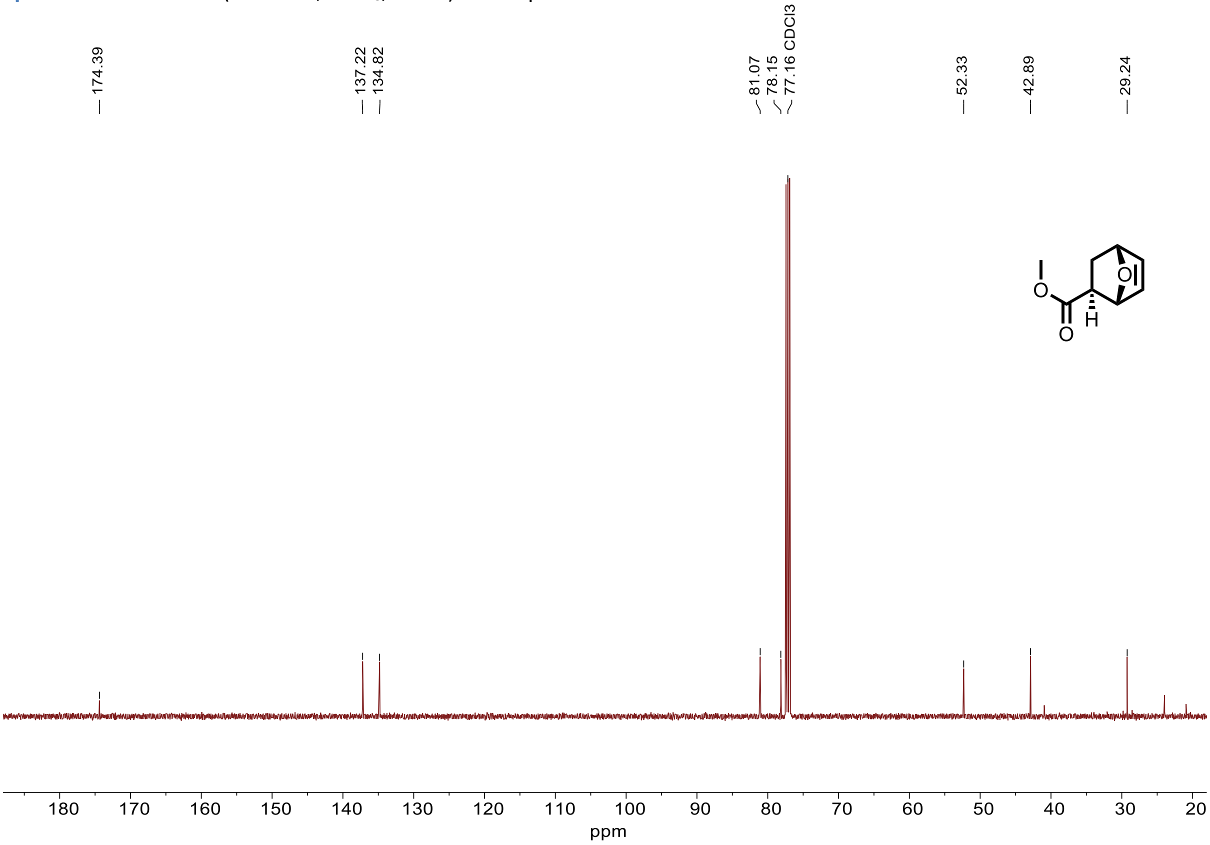

Spectrum S10. ^{13}C NMR (126 MHz, $CDCl_3$, 298 K) of compound **S7**.

5.6 Spectra of S8

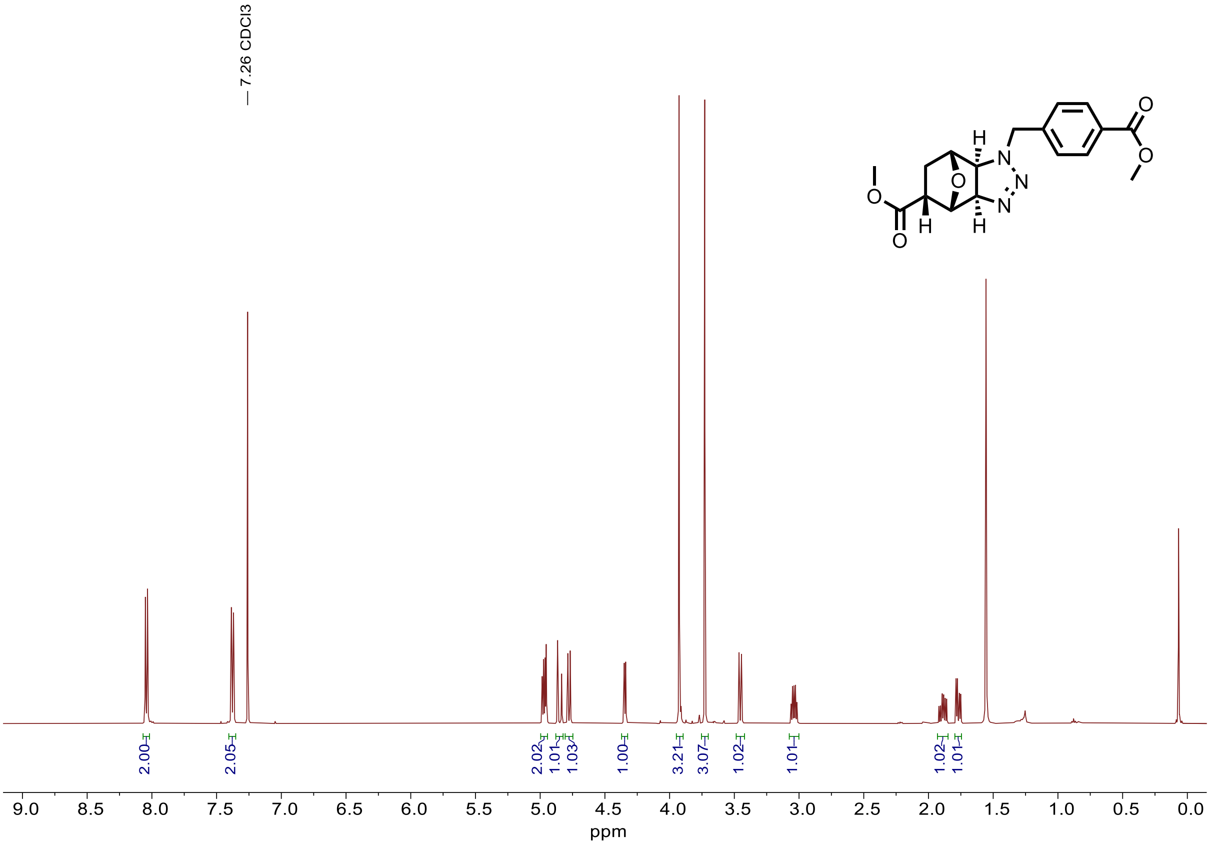

Spectrum S11. ^{1}H NMR (500 MHz, $CDCl_3$, 298 K) of compound **S8**.

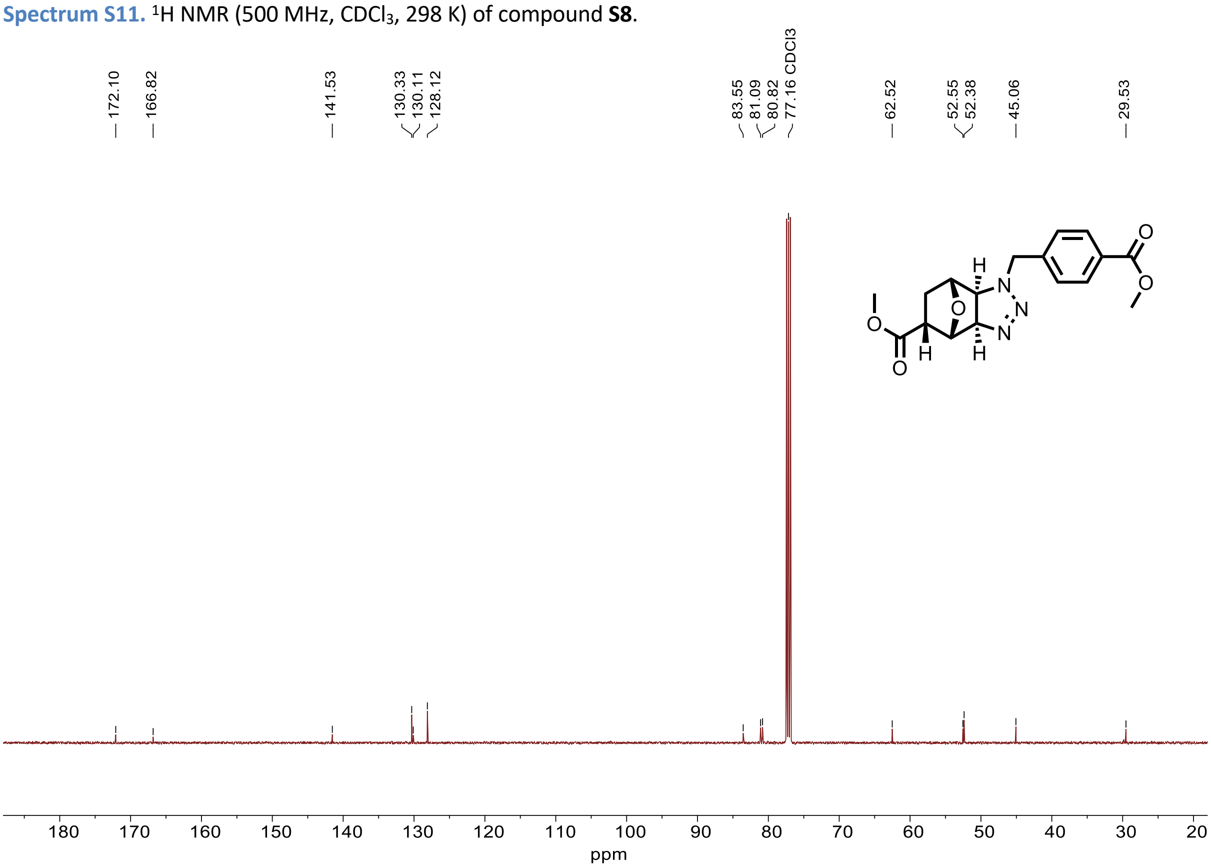

Spectrum S12. ^{13}C NMR (126 MHz, $CDCl_3$, 298 K) of compound **S8**.

5.7 Spectra of S9

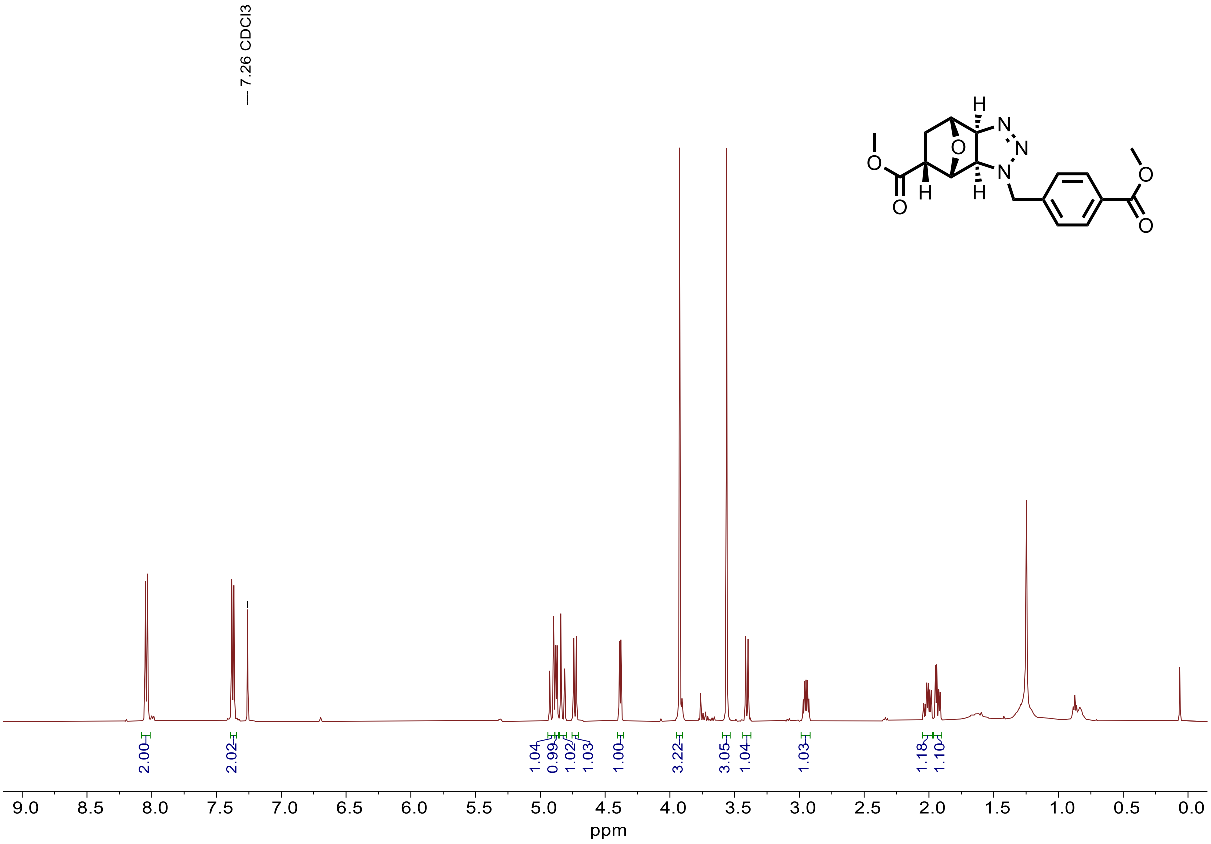

Spectrum S13. ^{1}H NMR (500 MHz, $CDCl_3$, 298 K) of compound **S9**.

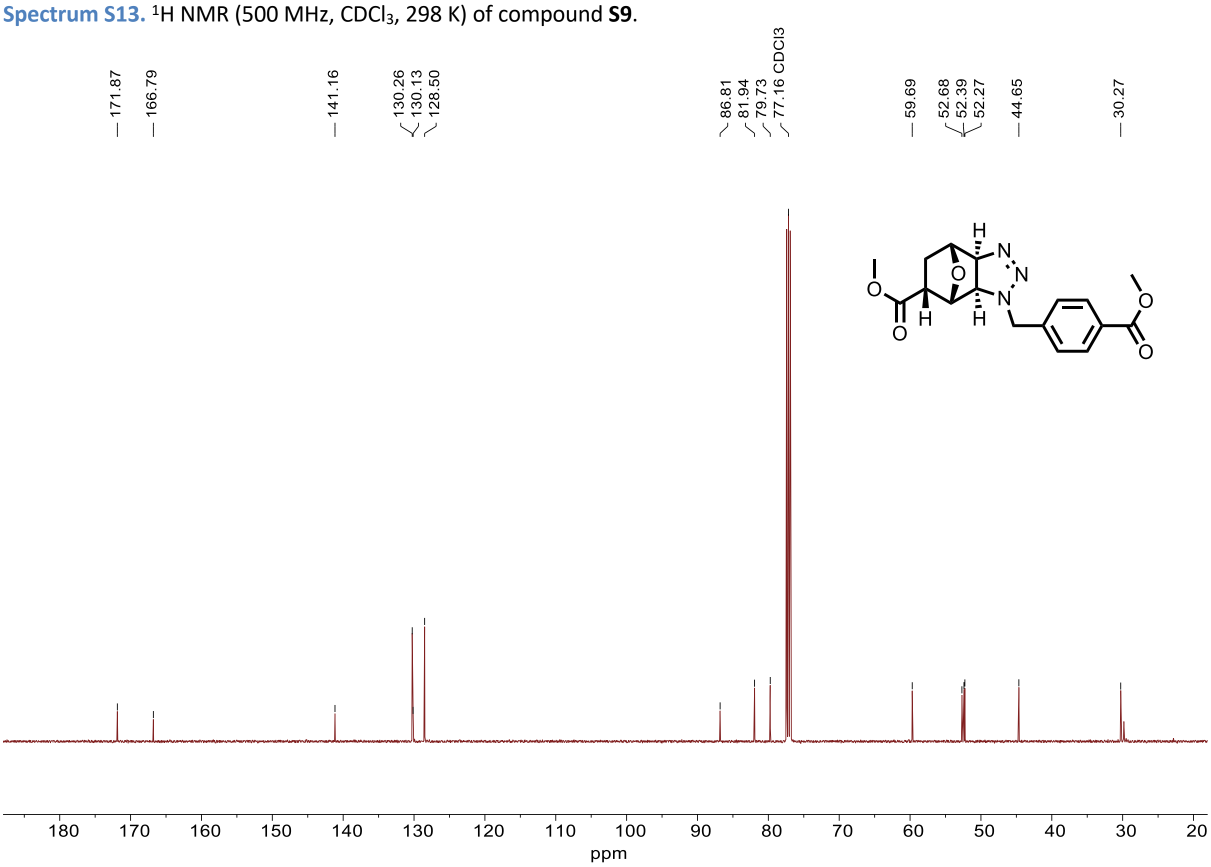

Spectrum S14. ^{13}C NMR (126 MHz, $CDCl_3$, 298 K) of compound **S9**.

5.8 Spectra of S10

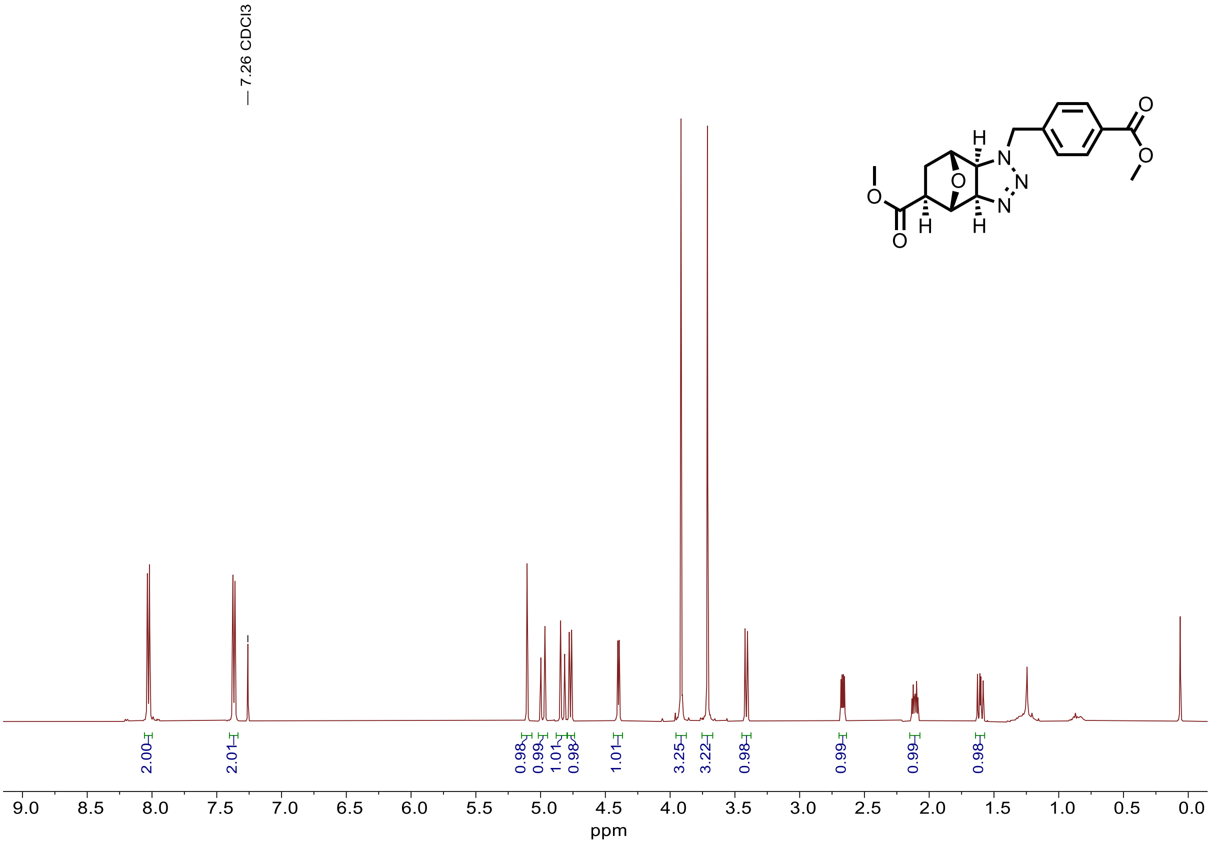

Spectrum S15. ^{1}H NMR (500 MHz, $CDCl_3$, 298 K) of compound **S10**.

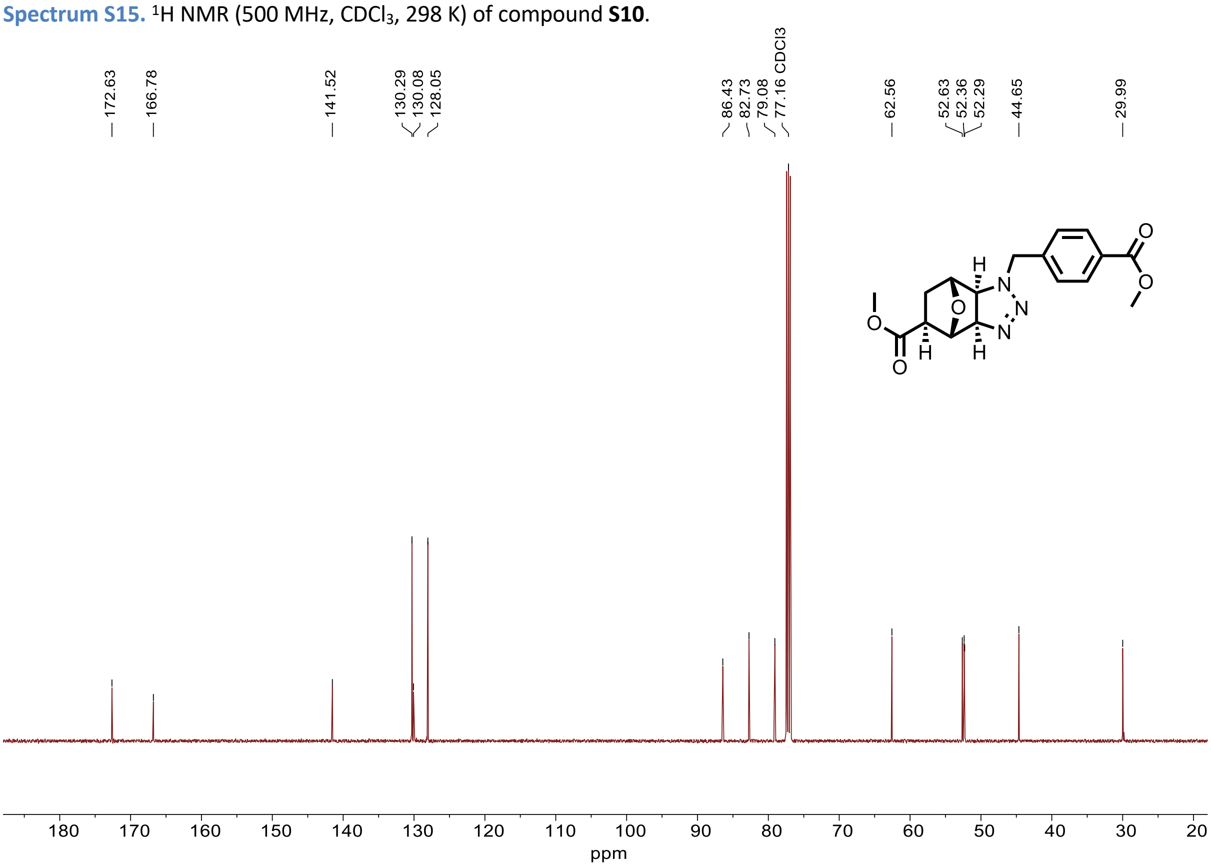

Spectrum S16. ^{13}C NMR (126 MHz, $CDCl_3$, 298 K) of compound **S10**.

5.9 Spectra of S11

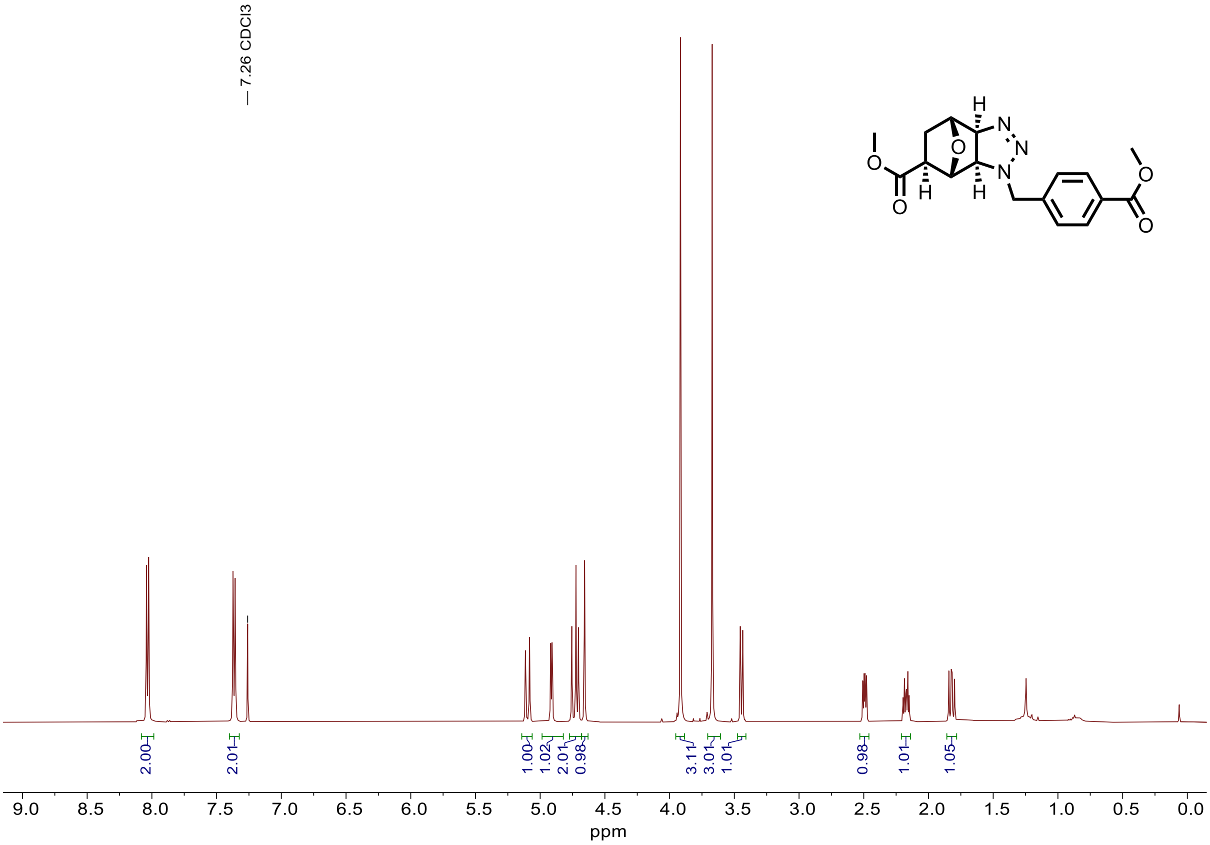

Spectrum S17. ^{1}H NMR (500 MHz, $CDCl_3$, 298 K) of compound **S11**.

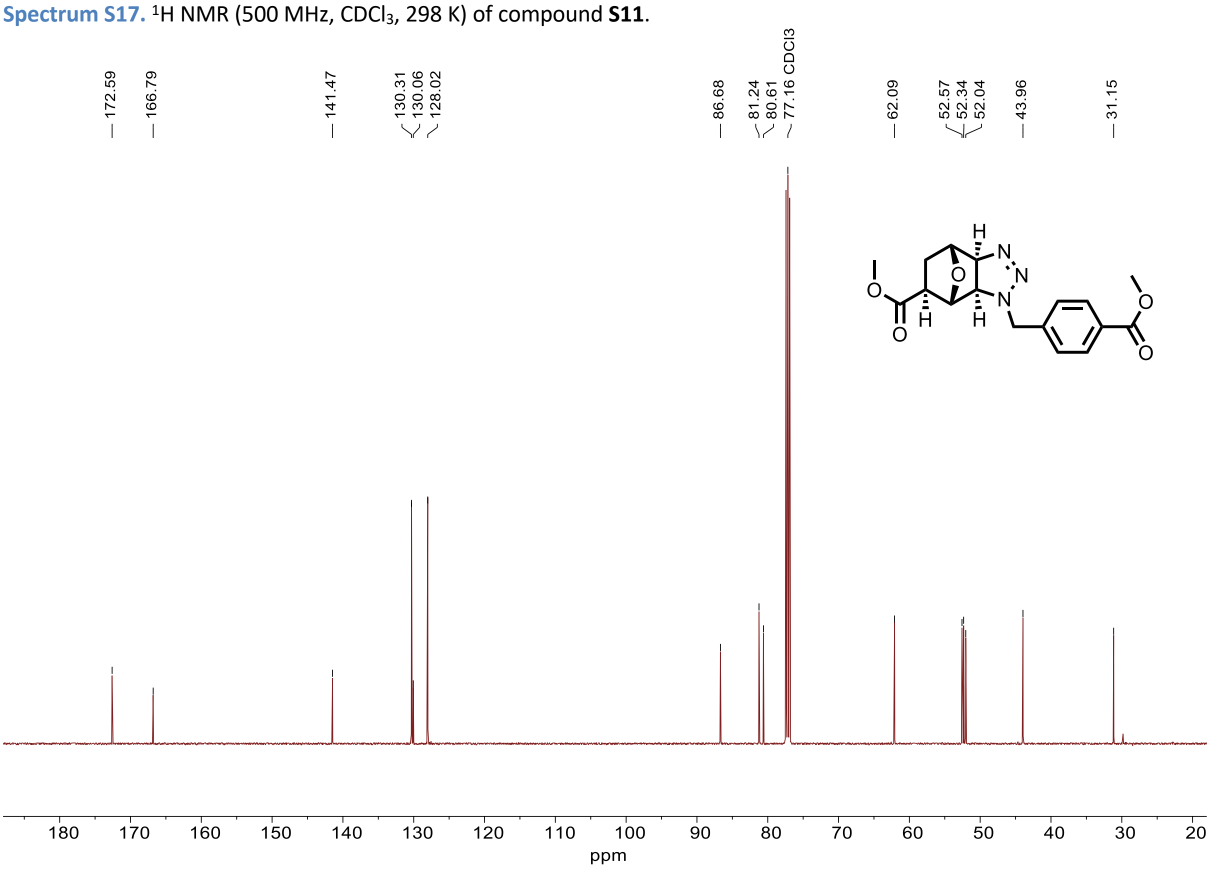

Spectrum S18. ^{13}C NMR (126 MHz, $CDCl_3$, 298 K) of compound **S11**.

5.10 Spectra of S14

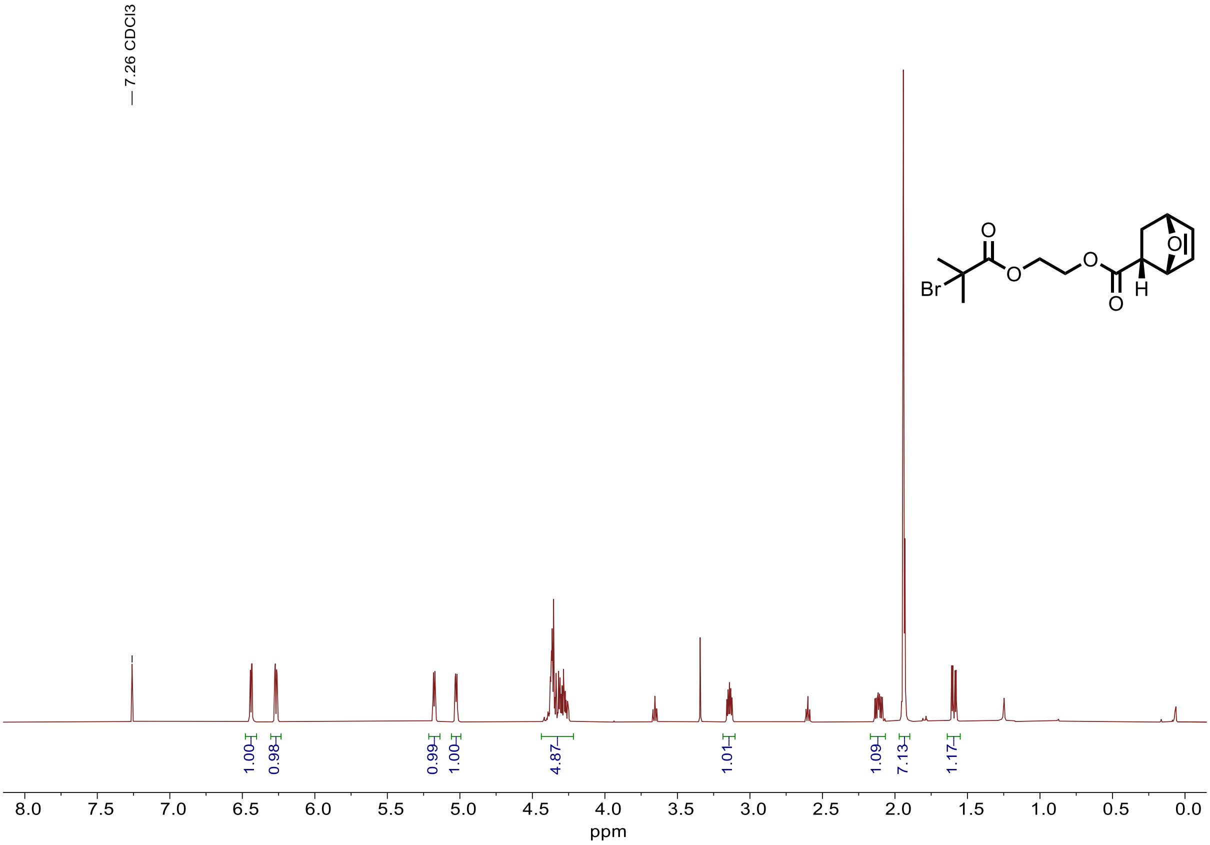

Spectrum S19. ^{1}H NMR (500 MHz, $CDCl_3$, 298 K) of compound **S14**.

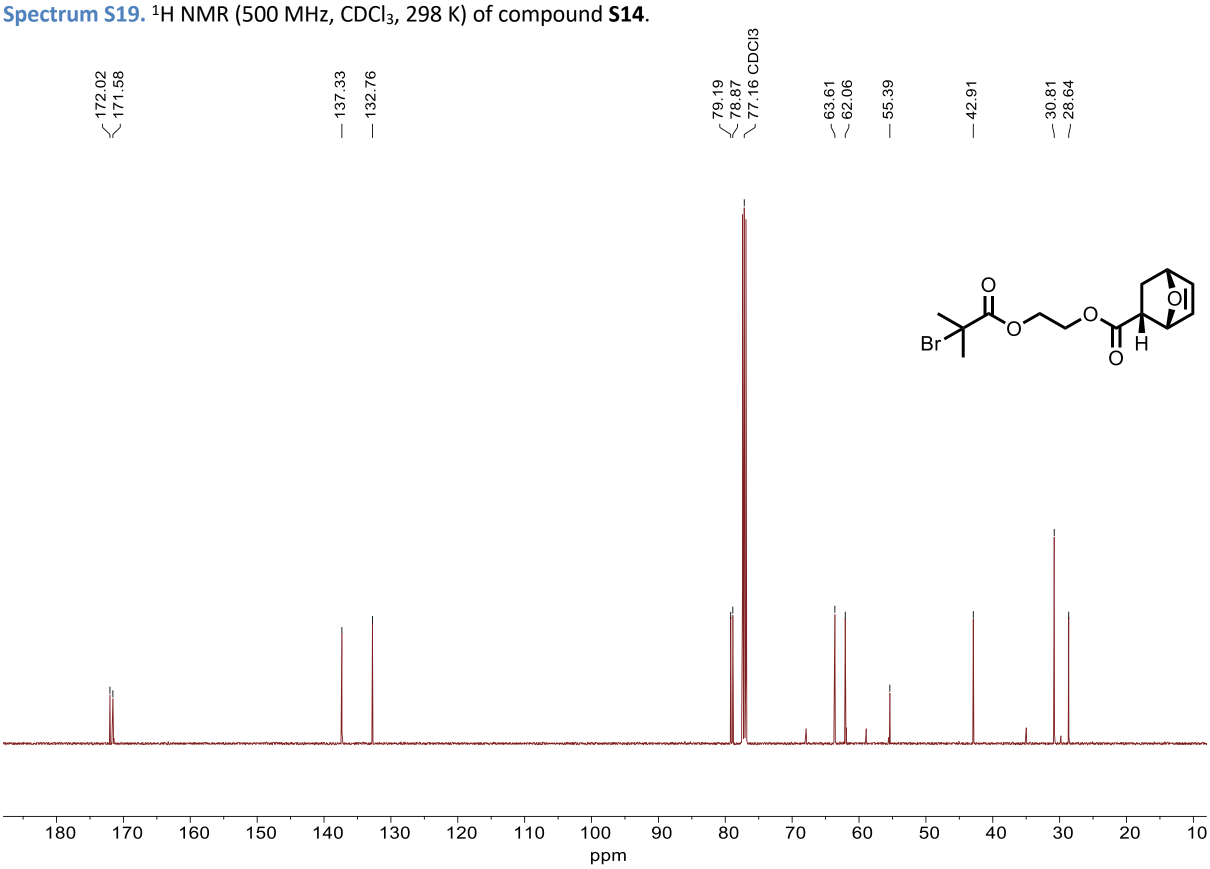

Spectrum S20. ^{13}C NMR (126 MHz, $CDCl_3$, 298 K) of compound **S14**.

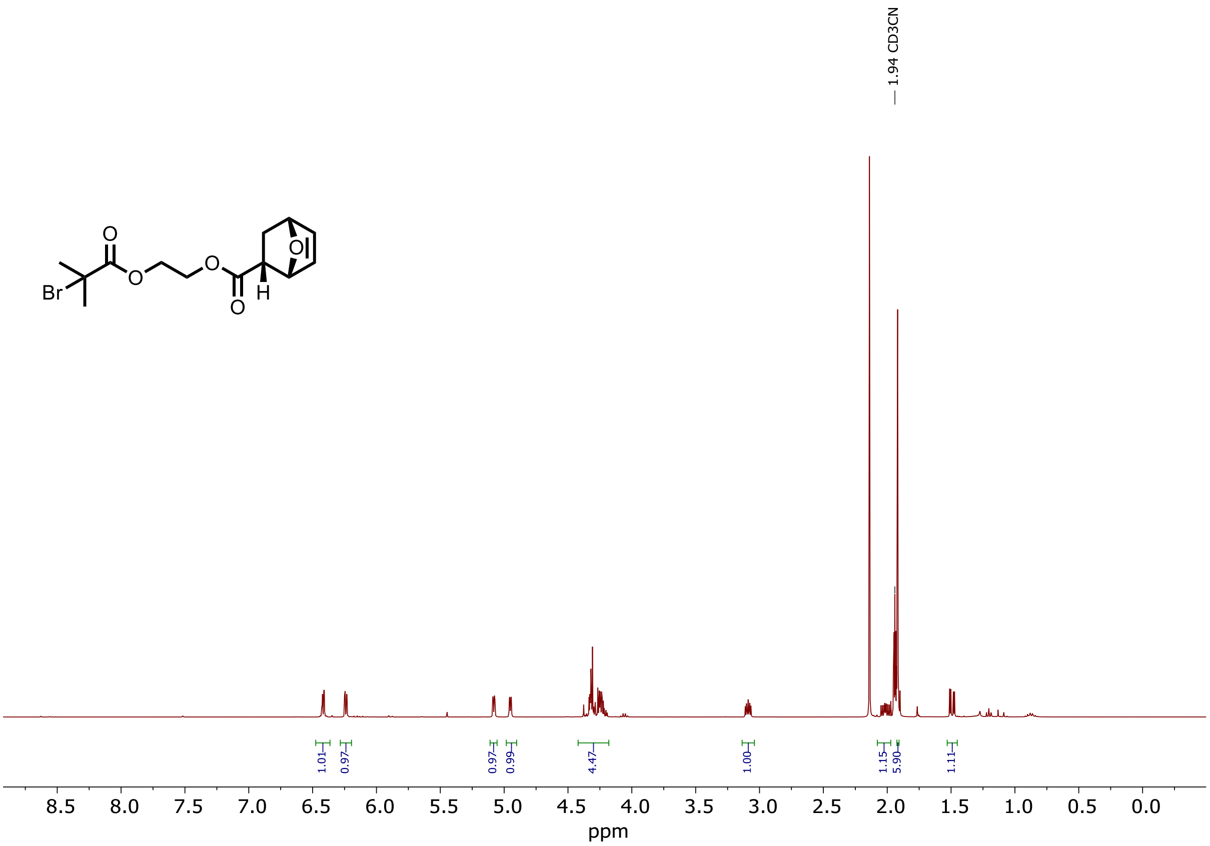

Spectrum S21. ^{1}H NMR (400 MHz, acetonitrile-d_3, 298 K, 16 scans) of compound **S-14**.

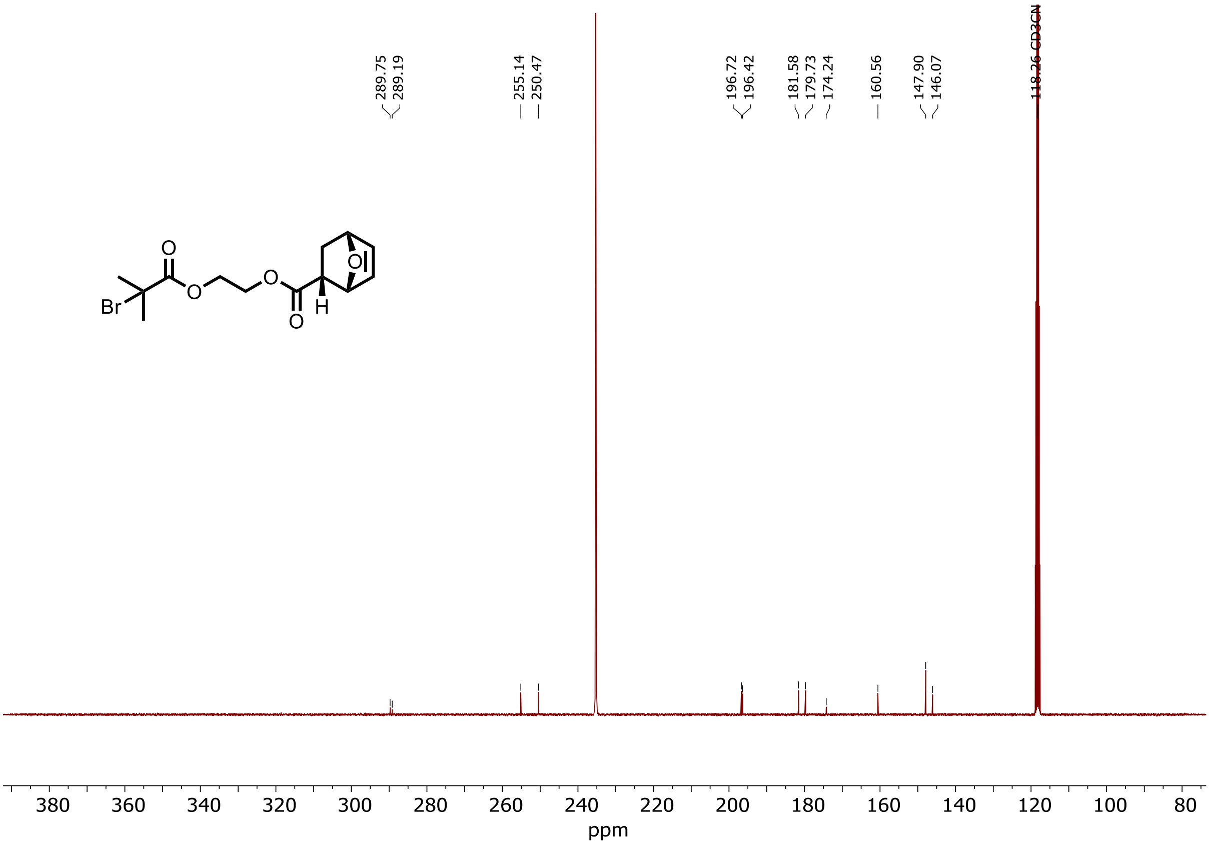

Spectrum S22. ^{13}C NMR (100 MHz, acetonitrile-d_3, 298 K, 256 scans) of compound **S14**.

5.11 Spectra of S15

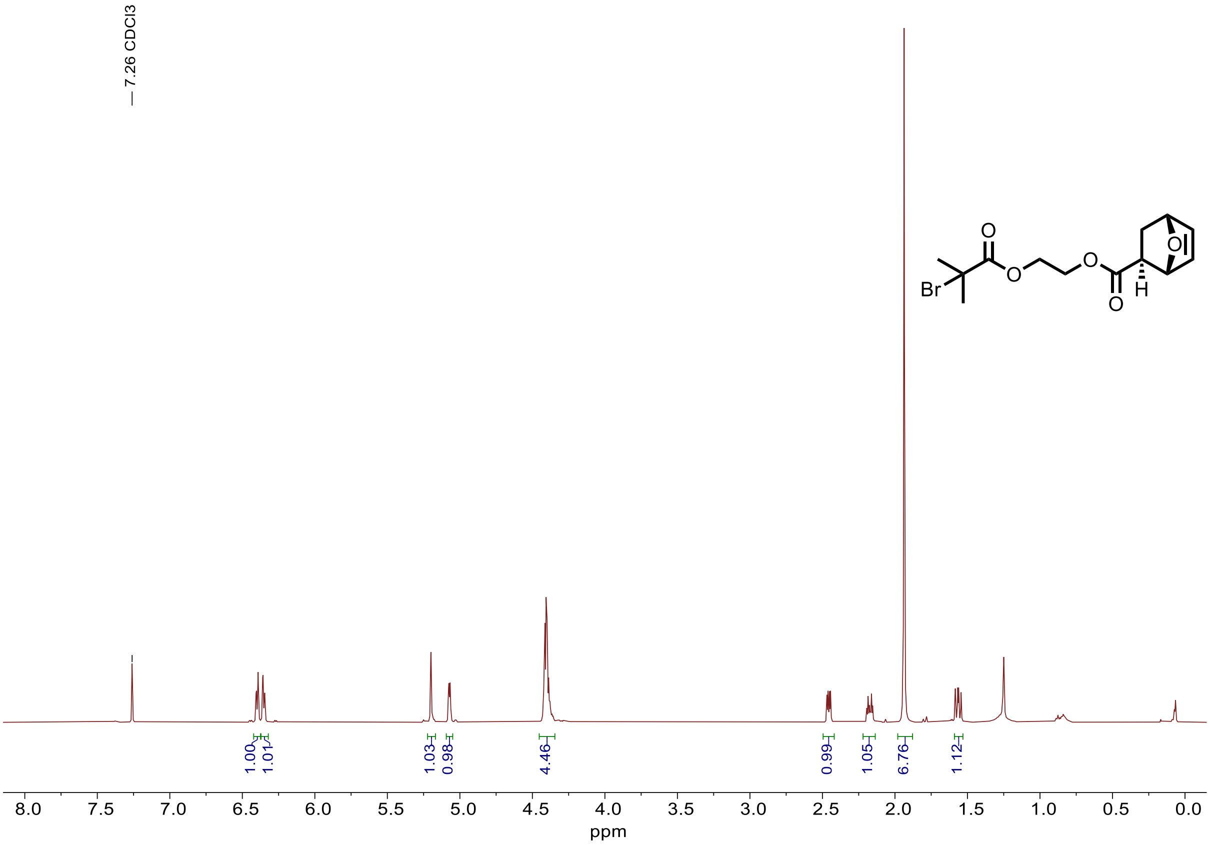

Spectrum S23. ^{1}H NMR (500 MHz, $CDCl_3$, 298 K) of compound **S15**.

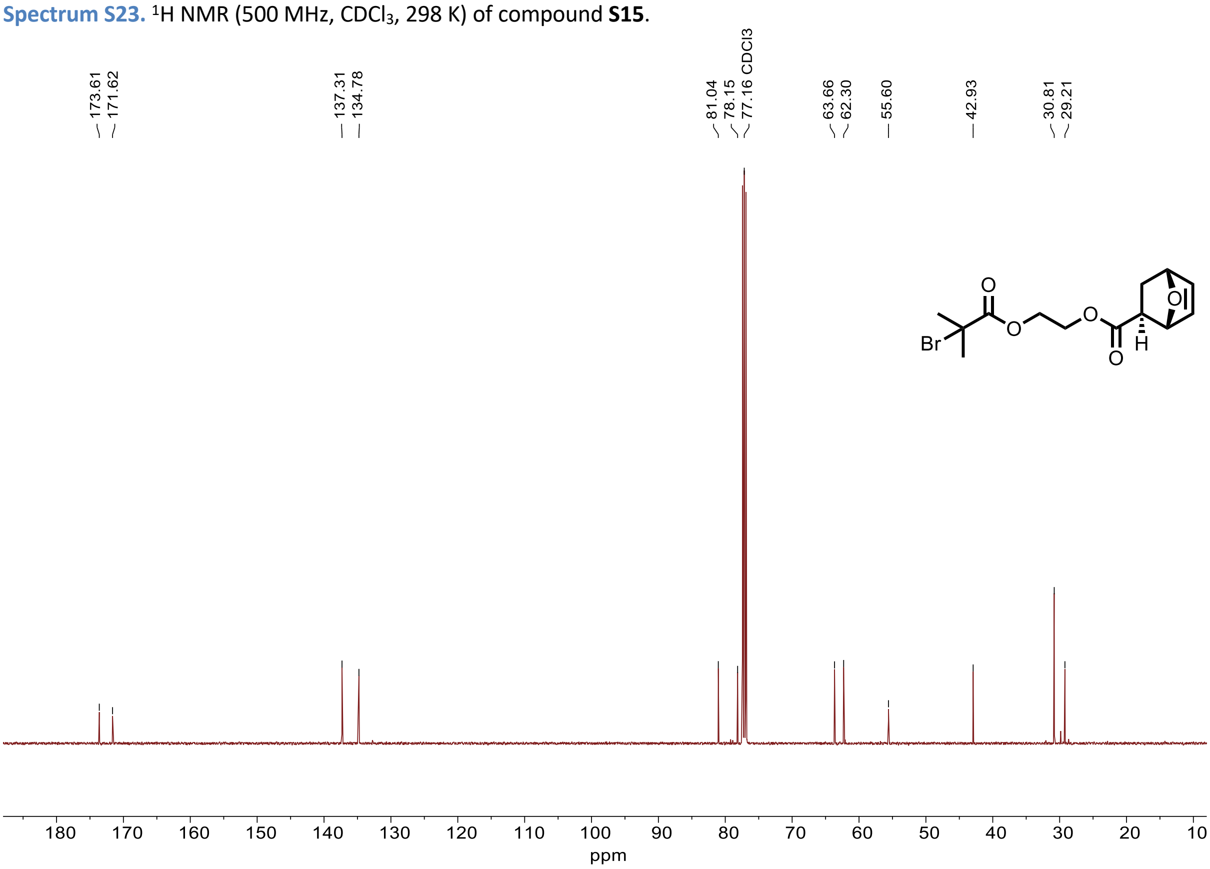

Spectrum S24. ^{13}C NMR (126 MHz, $CDCl_3$, 298 K) of compound **S15**.

5.12 Spectra of 1

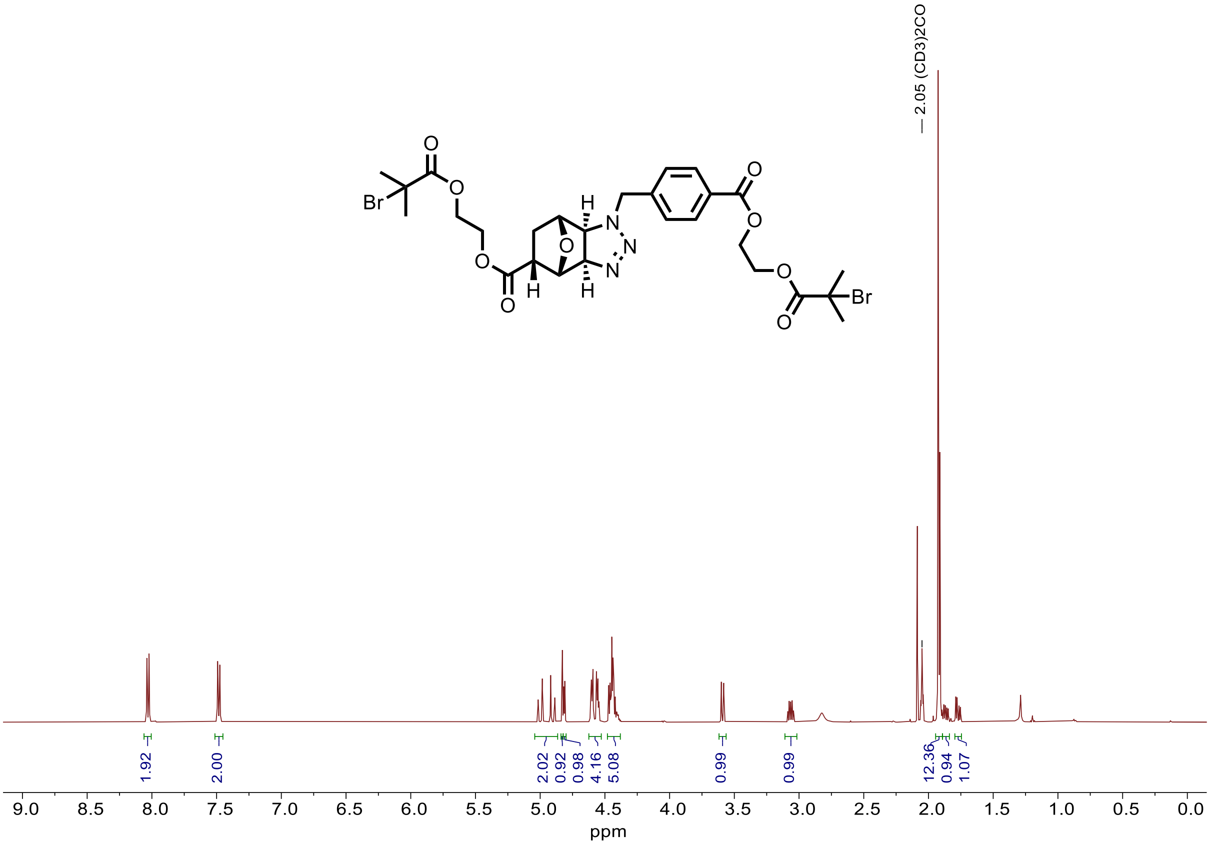

Spectrum S25. ^{1}H NMR (500 MHz, acetone-d_6, 298 K) of compound **1**.

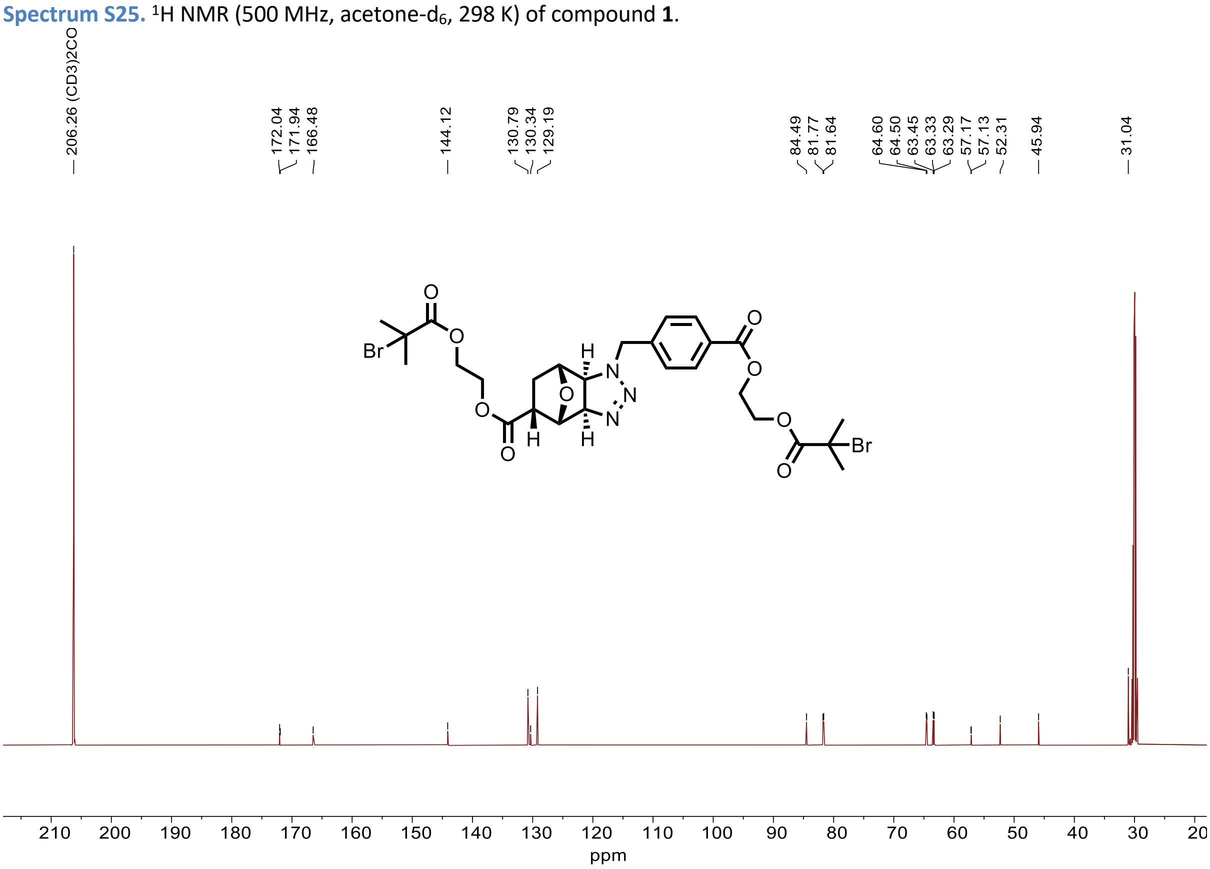

Spectrum S26. ^{13}C NMR (126 MHz, acetone-d_6, 298 K) of compound **1**.

5.13 Spectra of 2

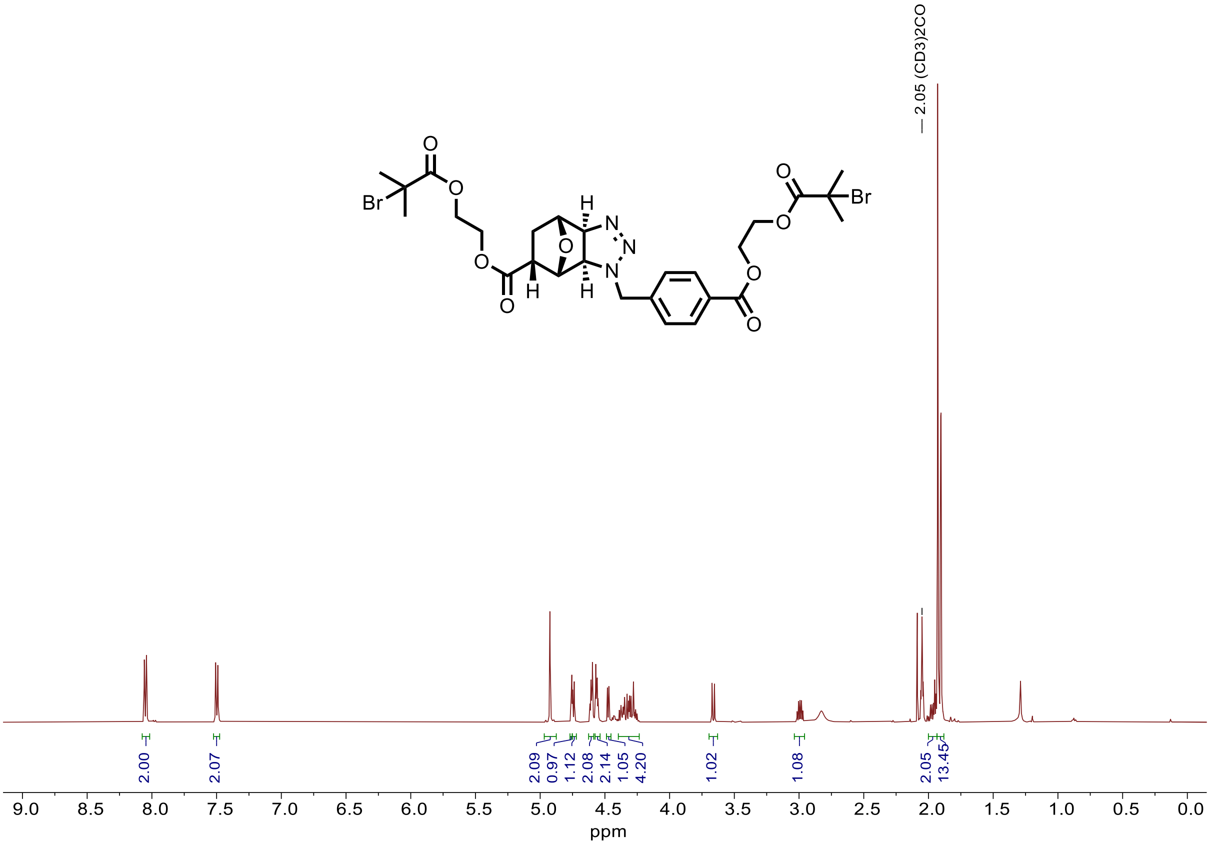

Spectrum S27. ^{1}H NMR (500 MHz, acetone-d_6, 298 K) of compound **2**.

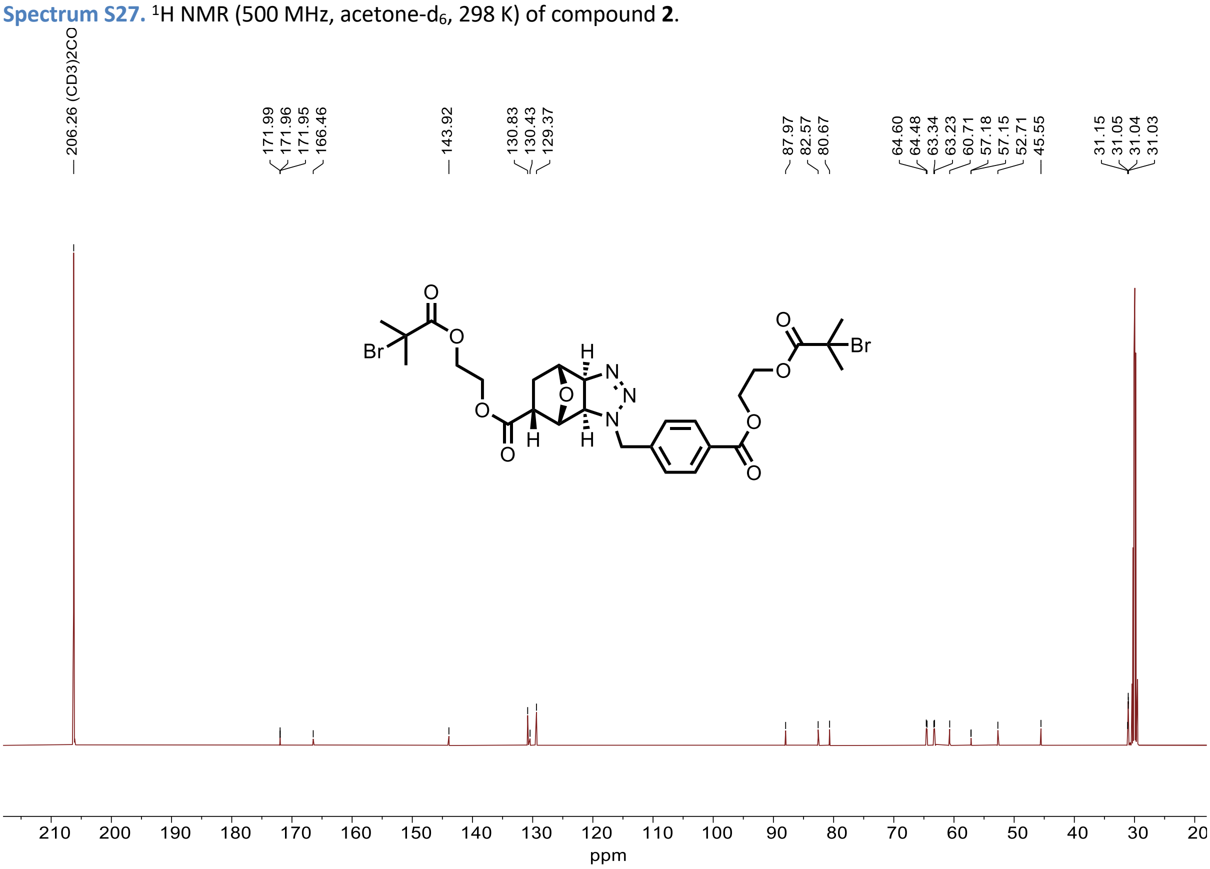

Spectrum S28. ^{13}C NMR (126 MHz, acetone-d_6, 298 K) of compound **2**.

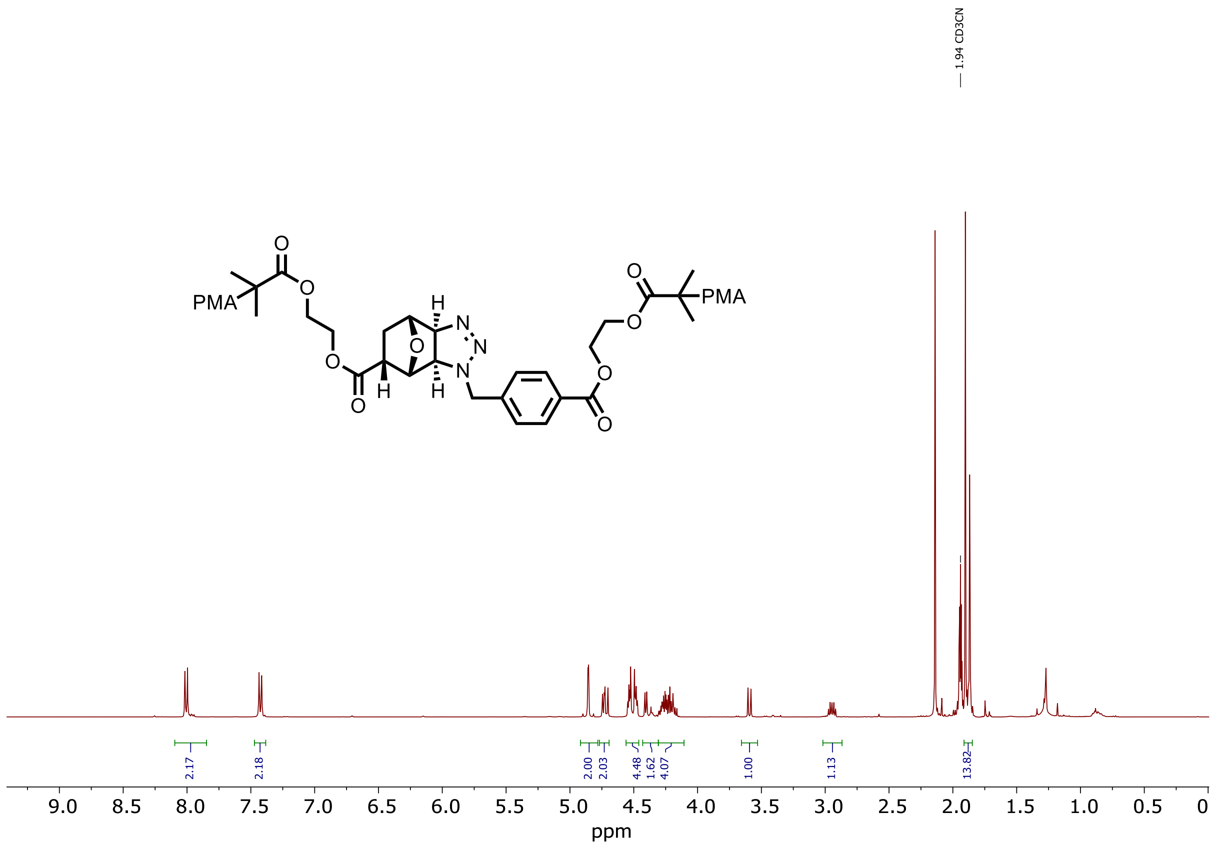

Spectrum S29. ^{1}H NMR (400 MHz, acetonitrile-d_3, 298 K, 16 scans) of Compound **2**.

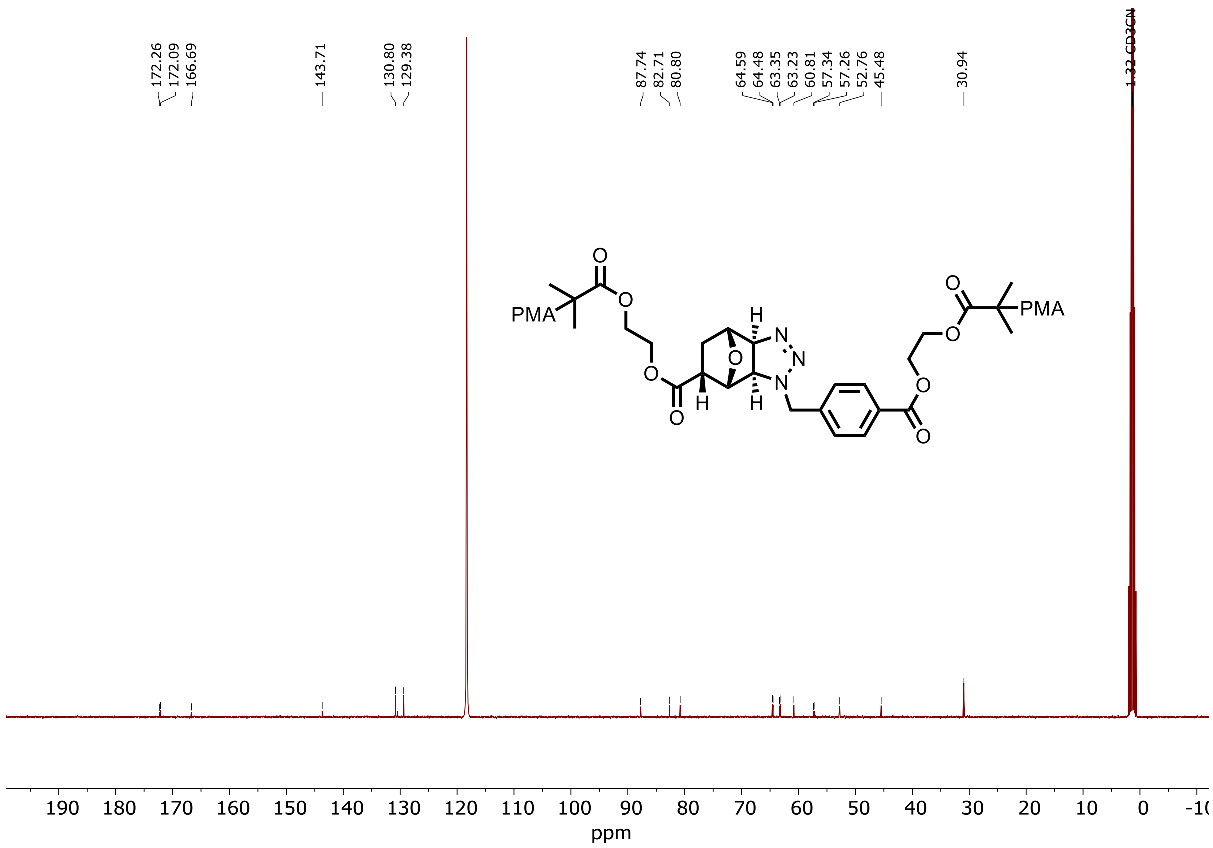

Spectrum S30. ^{13}C NMR (100 MHz, acetonitrile-d_3, 298 K, 256 scans) of compound **2**.

5.14 Spectra of 3

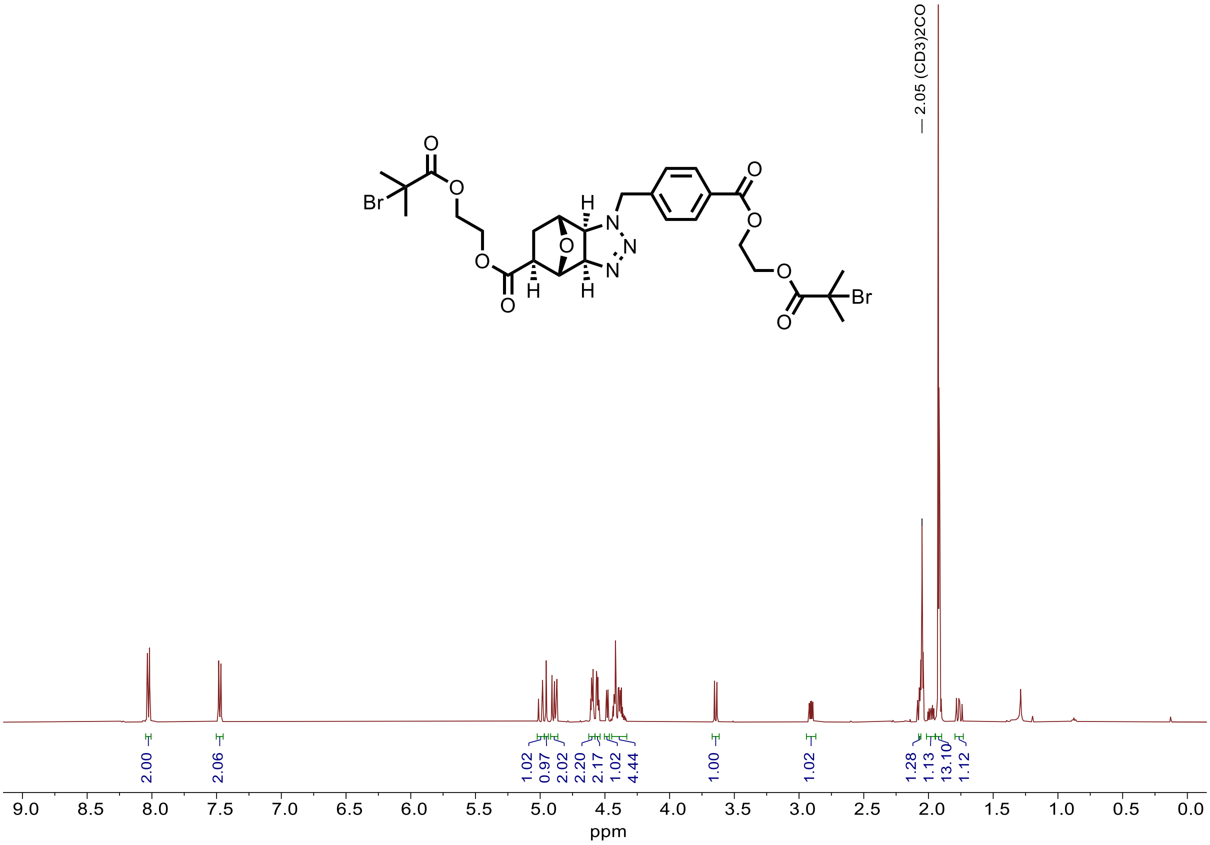

Spectrum S31. ^{1}H NMR (500 MHz, acetone-d$_6$, 298 K) of compound **3**.

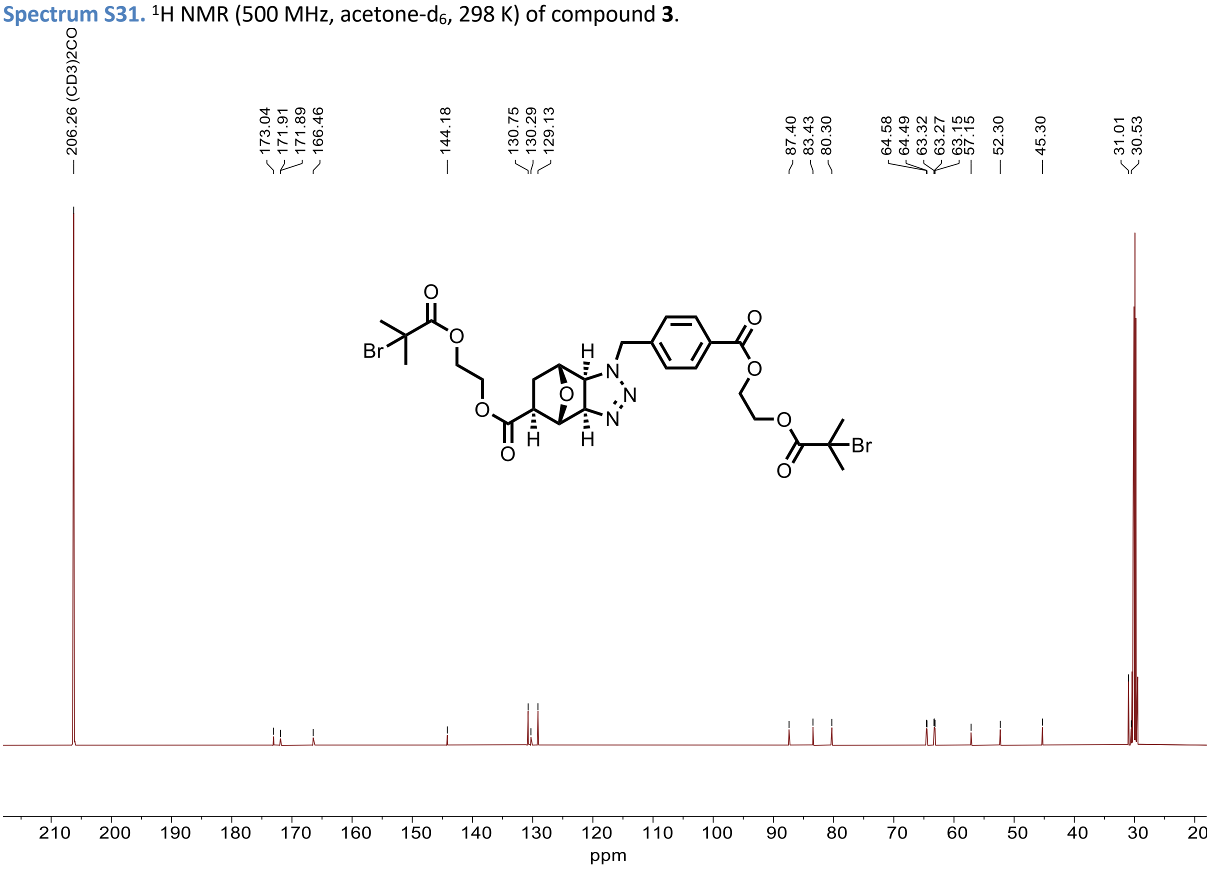

Spectrum S32. ^{13}C NMR (126 MHz, acetone-d$_6$, 298 K) of compound **3**.

5.15 Spectra of 4

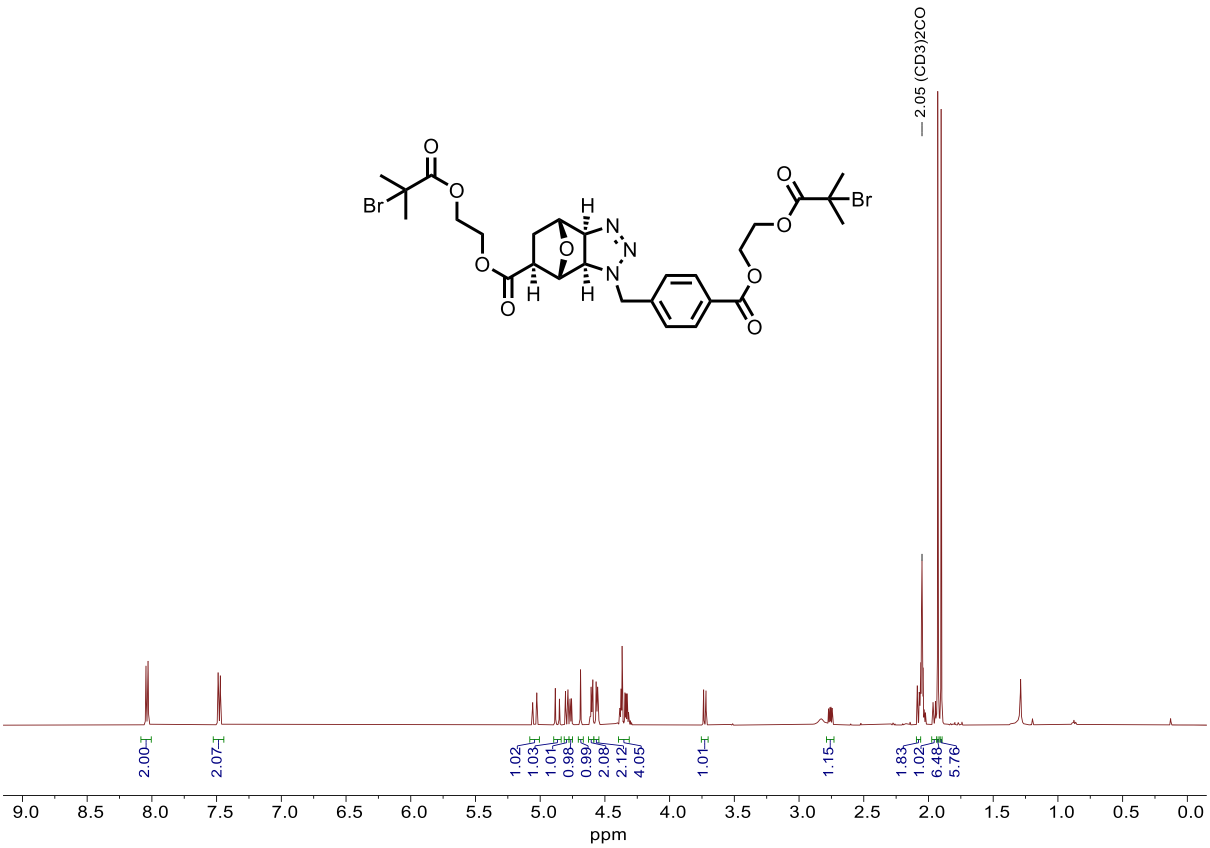

Spectrum S33. ^{1}H NMR (500 MHz, acetone-d$_6$, 298 K) of compound **4**.

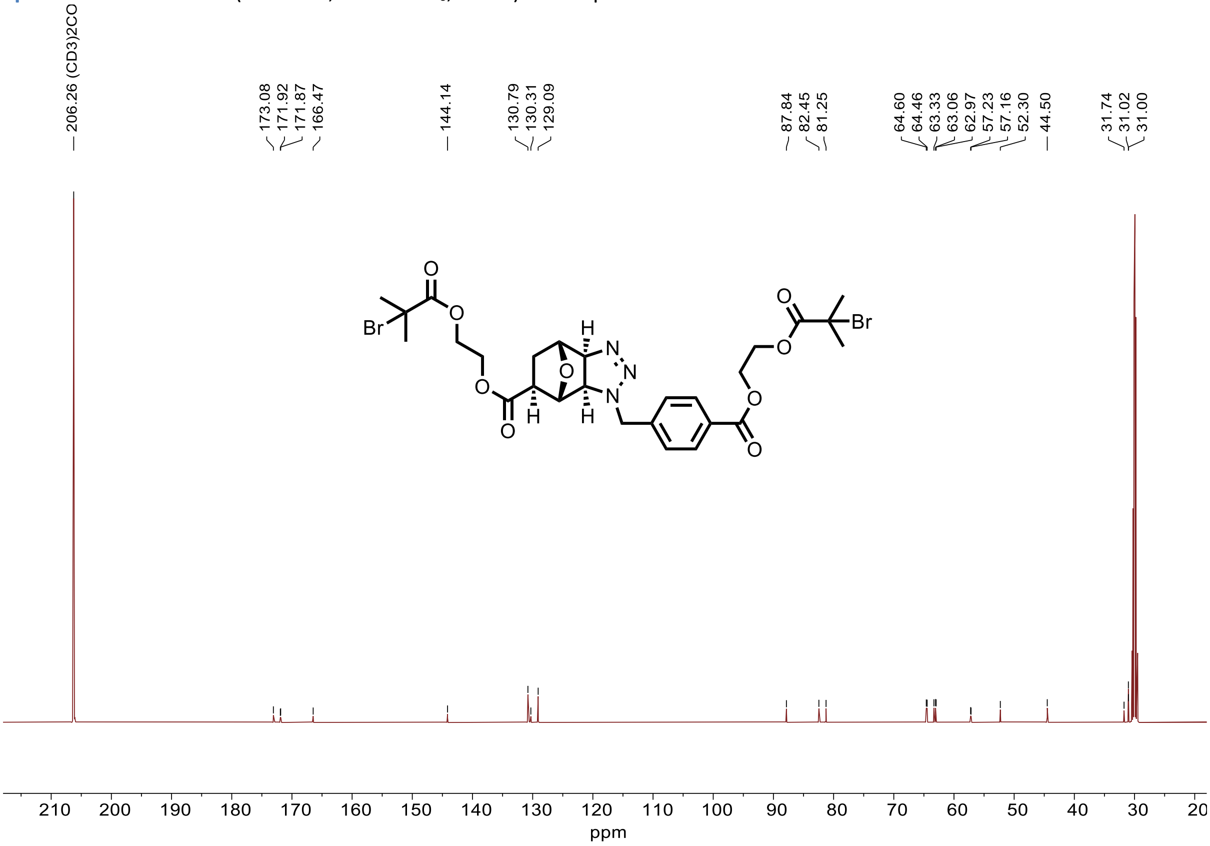

Spectrum S34. ^{13}C NMR (126 MHz, acetone-d$_6$, 298 K) of compound **4**.

5.16 Spectra of P-1

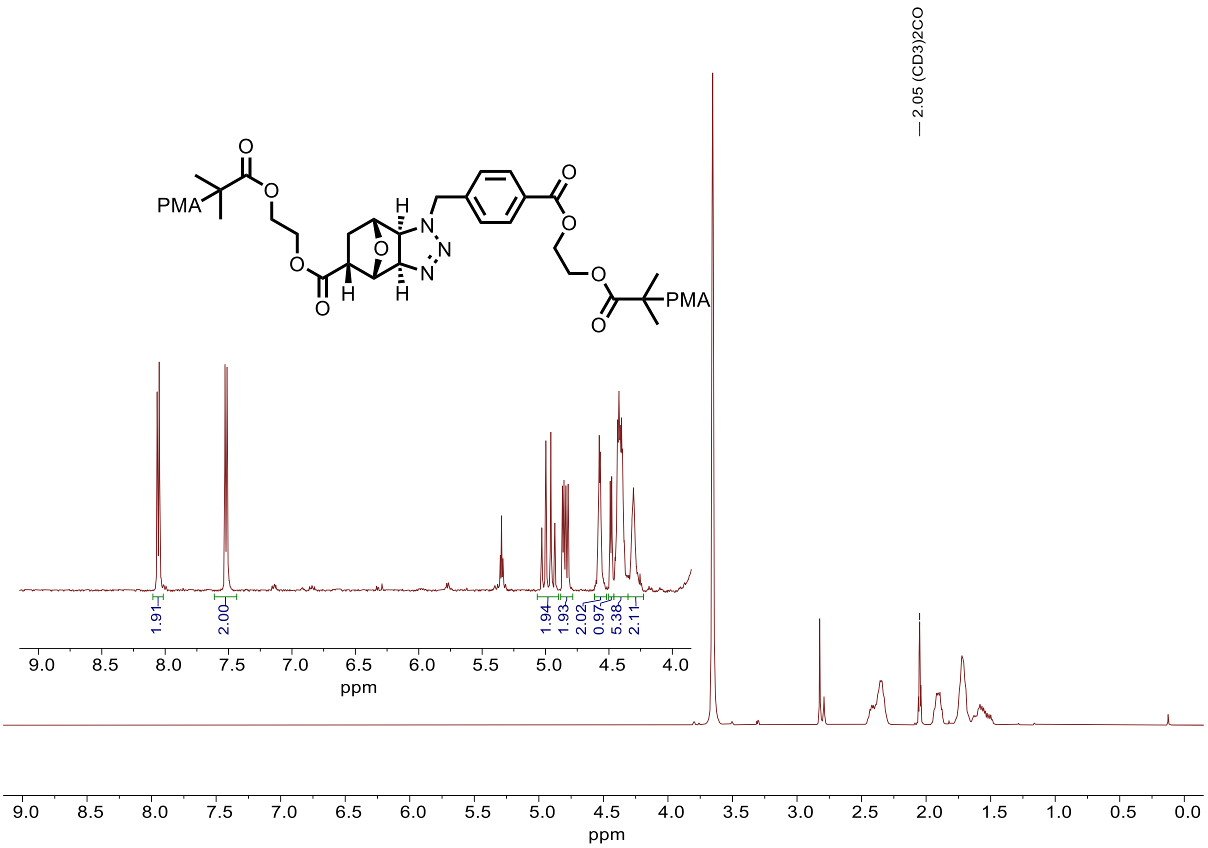

Spectrum S35. ^{1}H NMR (500 MHz, acetone-d$_6$, 298 K, 1024 scans) of polymer **P-1**.

5.17 Spectra of P-2

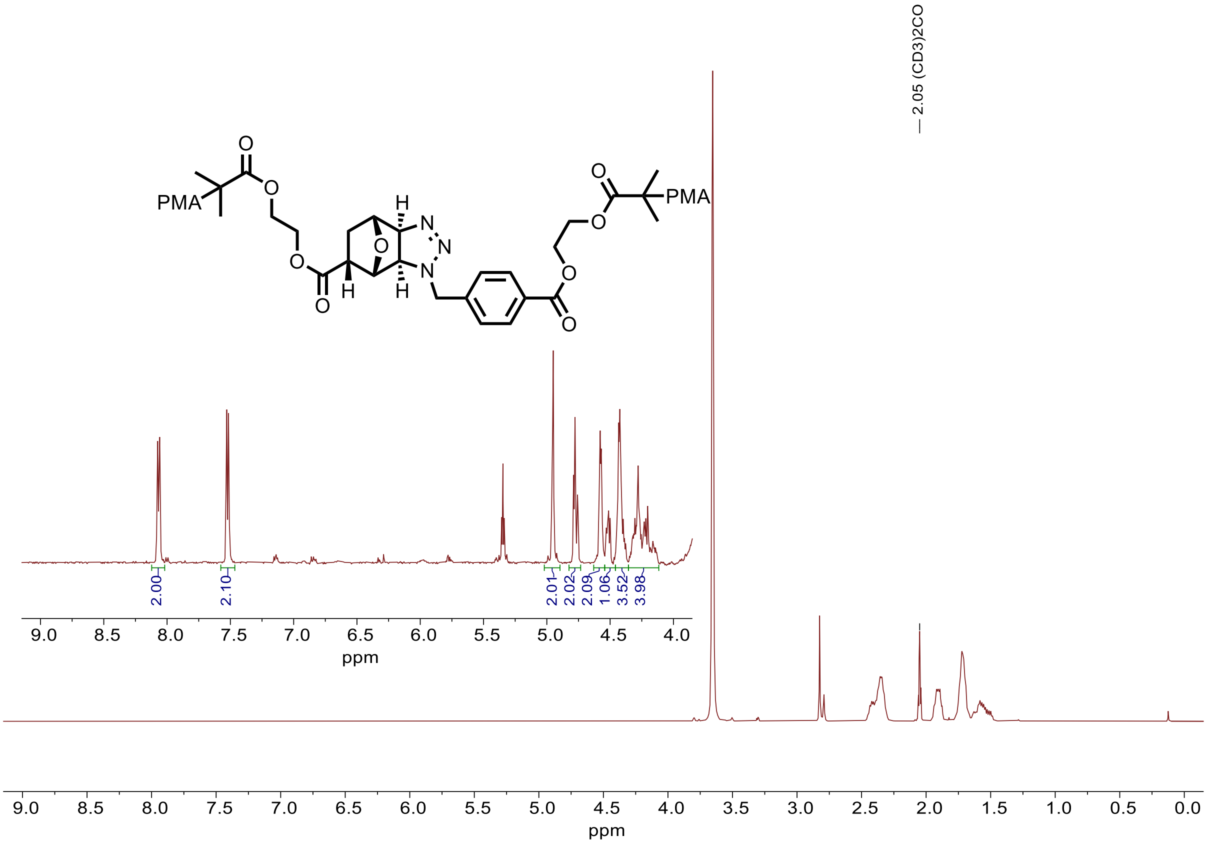

Spectrum S36. ^{1}H NMR (500 MHz, acetone-d_6, 298 K, 1024 scans) of polymer **P-2**.

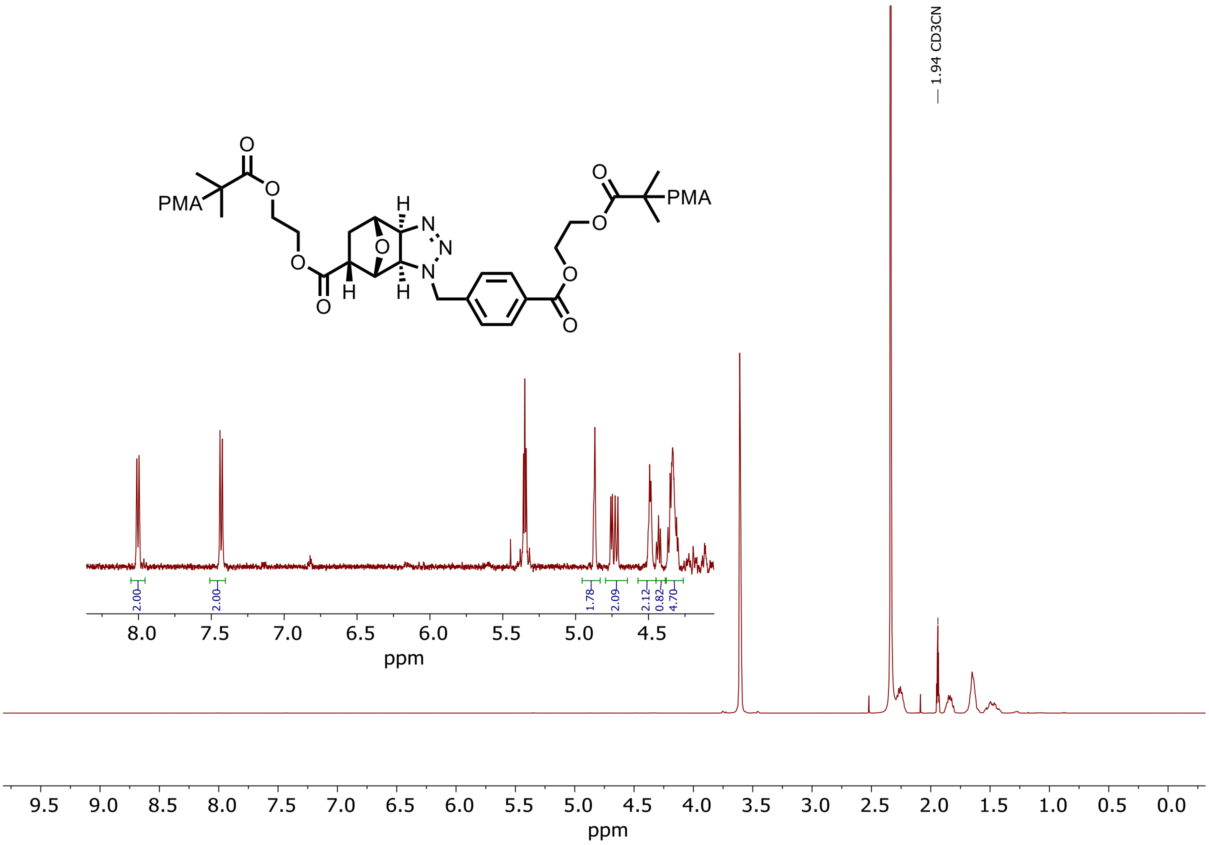

Spectrum S37. ^{1}H NMR (500 MHz, acetonitrile-d_3, 298 K, 256 scans) of polymer **P-2**.

5.18 Spectra of P-3

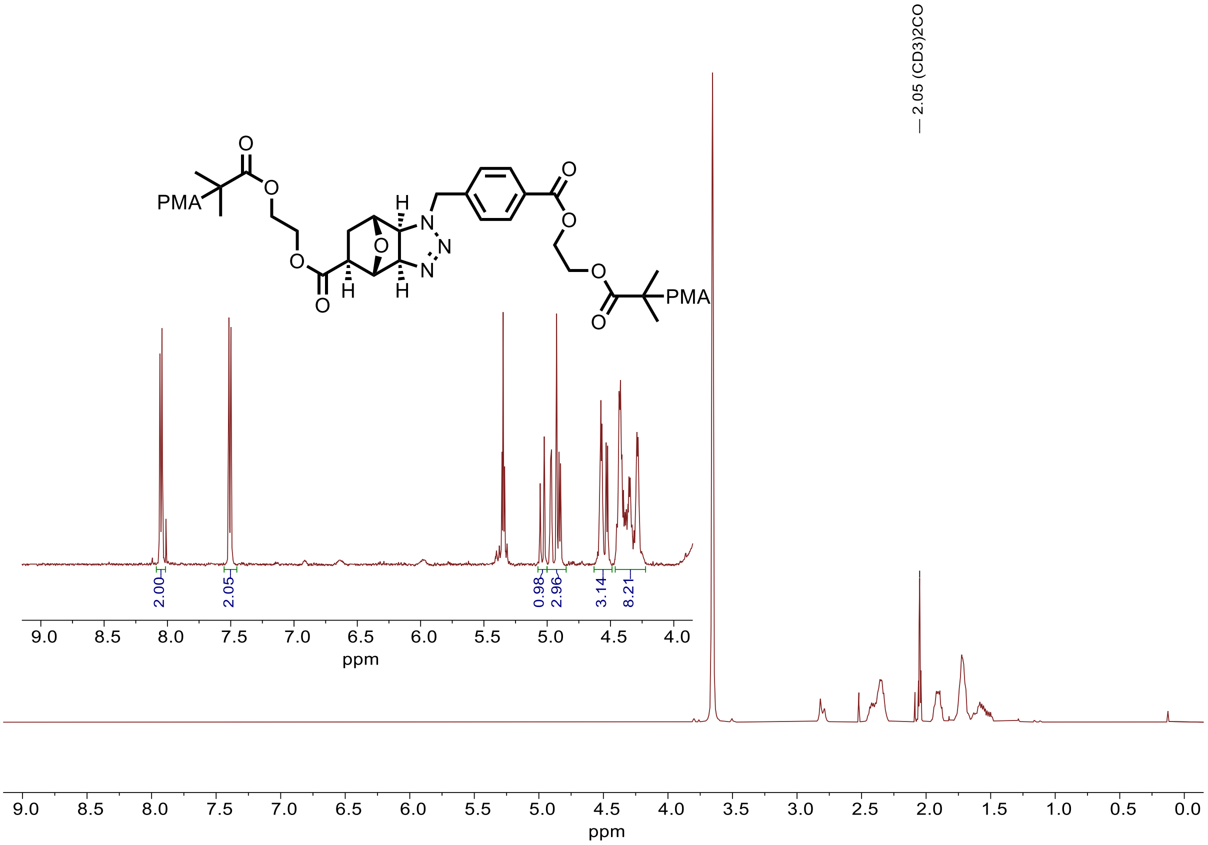

Spectrum S38. ^{1}H NMR (500 MHz, acetone-d_6, 298 K, 1024 scans) of polymer **P-3**.

5.19 Spectra of P-4

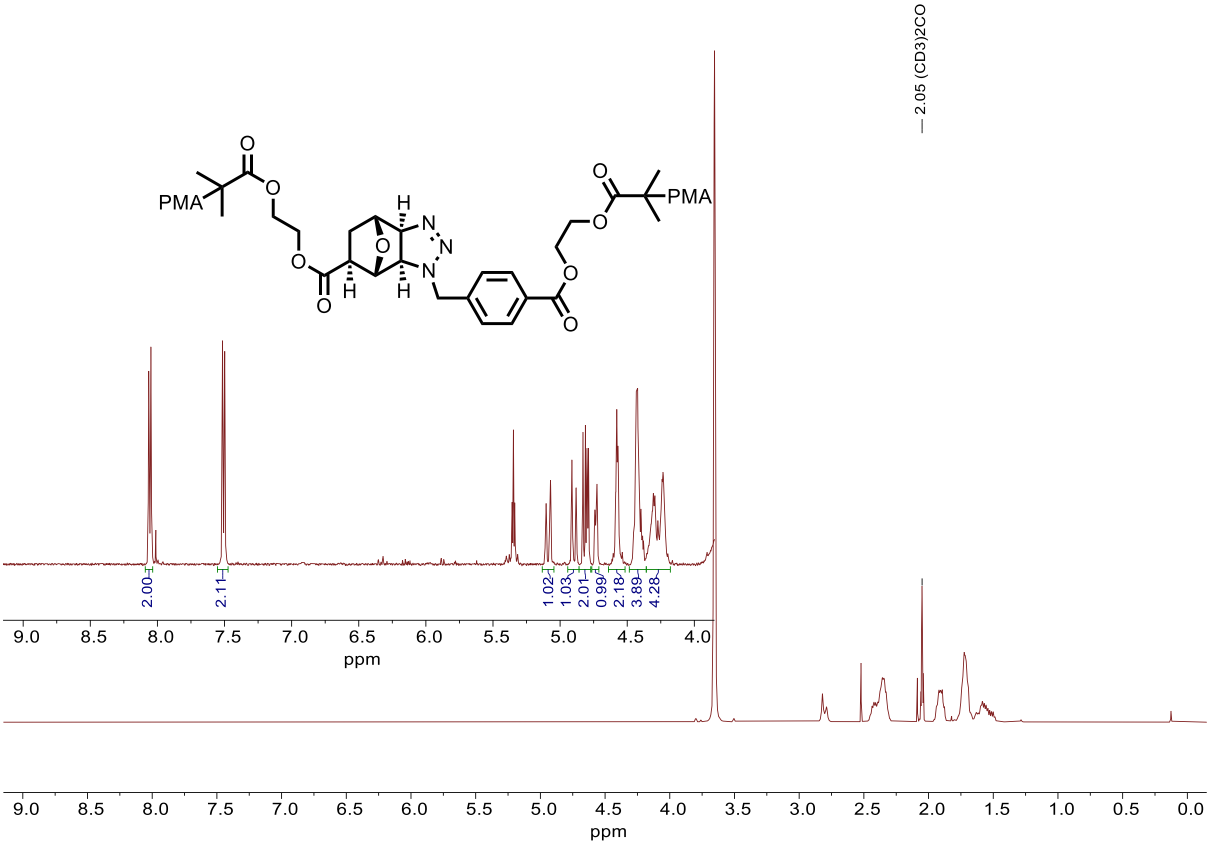

Spectrum S39. ^{1}H NMR (500 MHz, acetone-d_6, 298 K, 1024 scans) of polymer **P-4**.

5.20 Spectra of P-S5

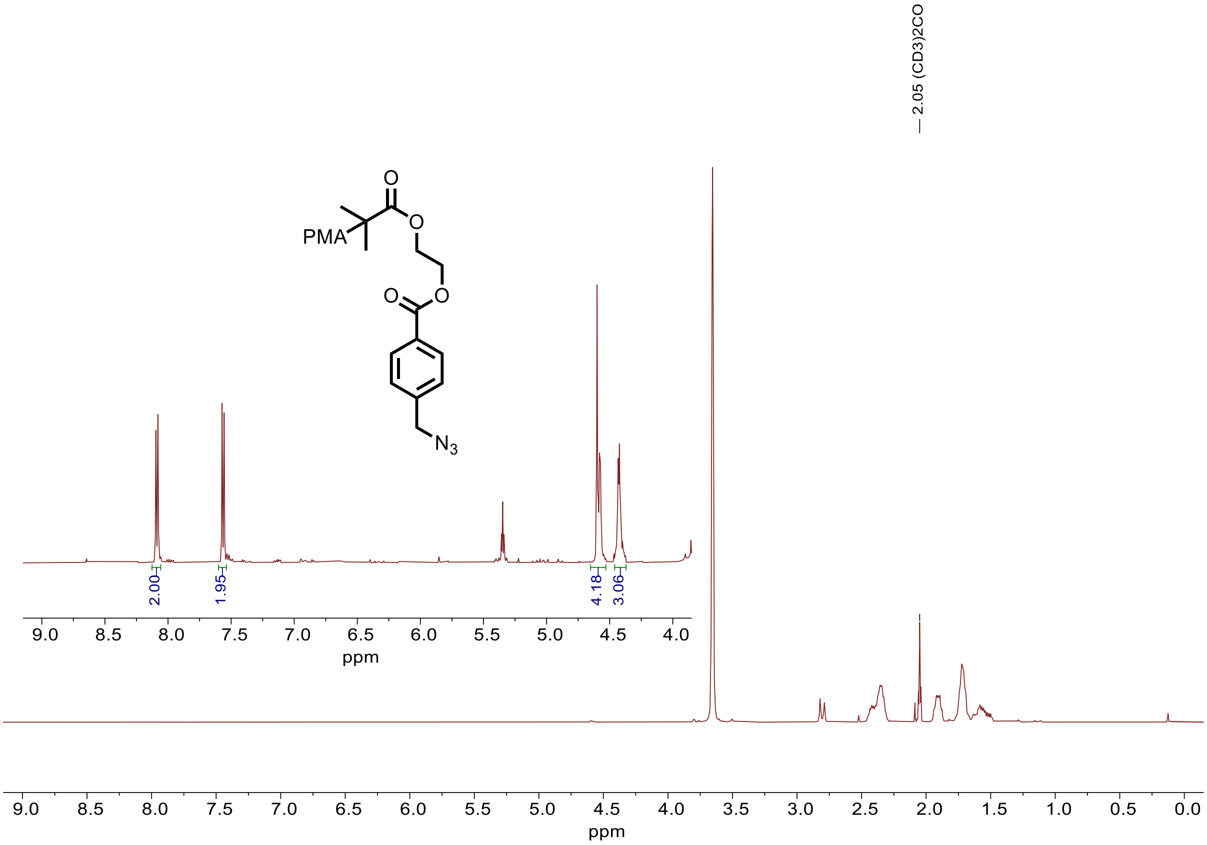

Spectrum S40. ^{1}H NMR (500 MHz, acetone-d$_6$, 298 K, 1024 scans) of polymer **P-S5**.

5.21 Spectra of P-S14

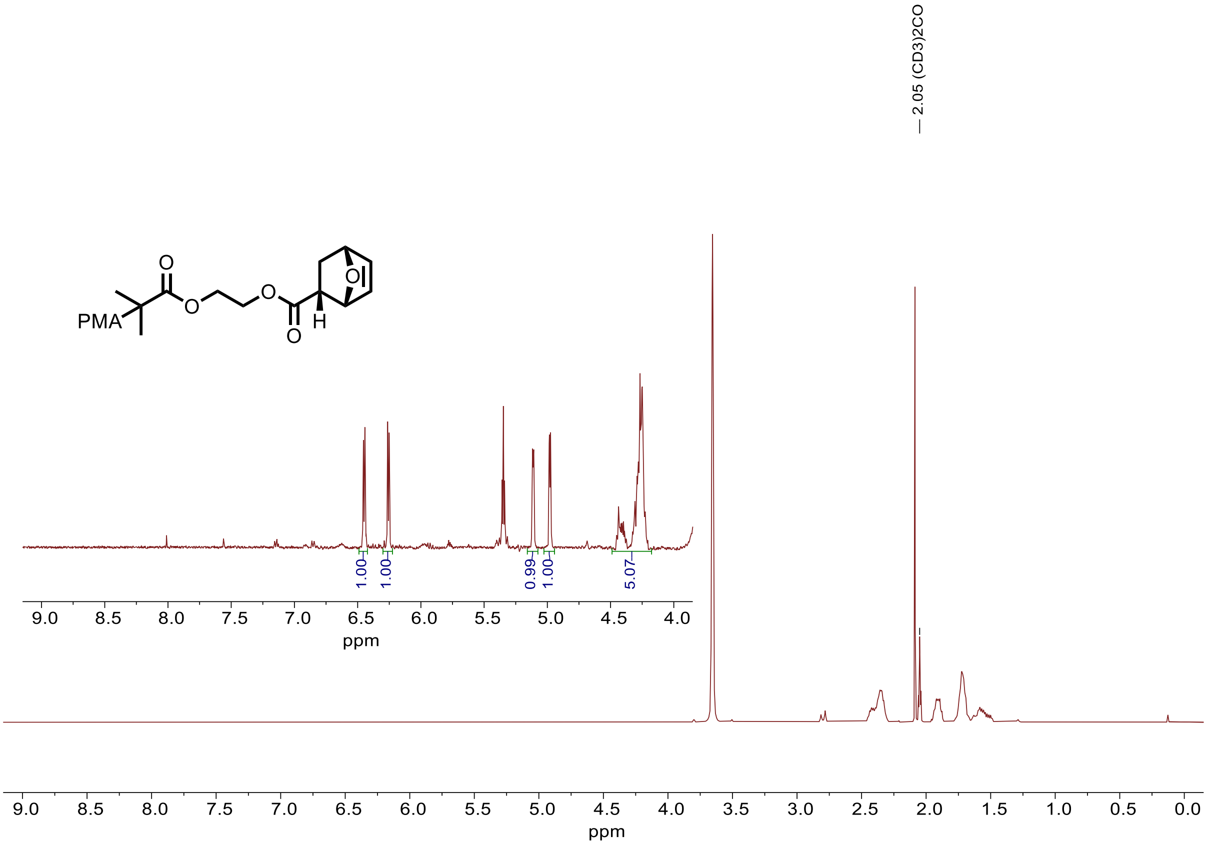

Spectrum S41. ^{1}H NMR (500 MHz, acetone-d_6, 298 K, 1024 scans) of polymer **P-S14**.

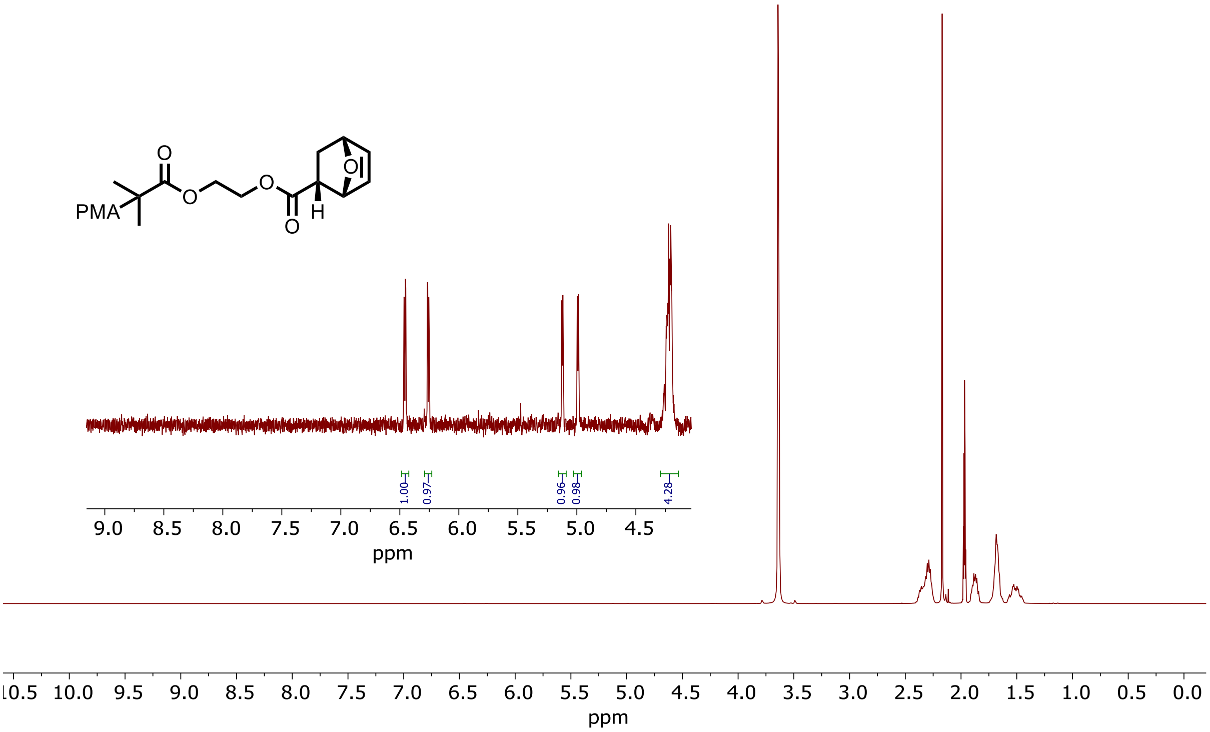

Spectrum S42. ^{1}H NMR (500 MHz, acetonitrile-d_3, 298 K, 256 scans) of polymer **P-S14**.

5.22 Spectra of P-S15

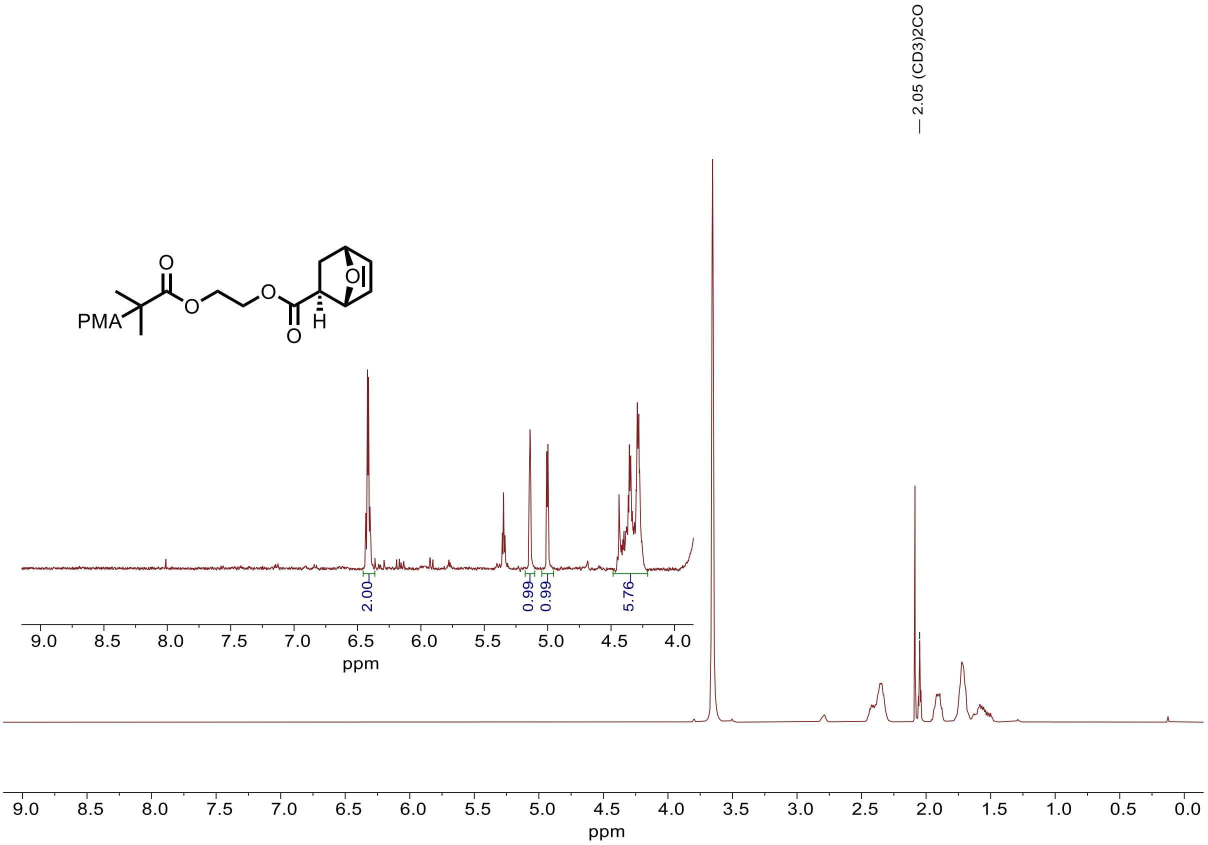

Spectrum S43. ^{1}H NMR (500 MHz, acetone-d_6, 298 K, 1024 scans) of polymer **P-S15**.

5.23 Spectra of PMA

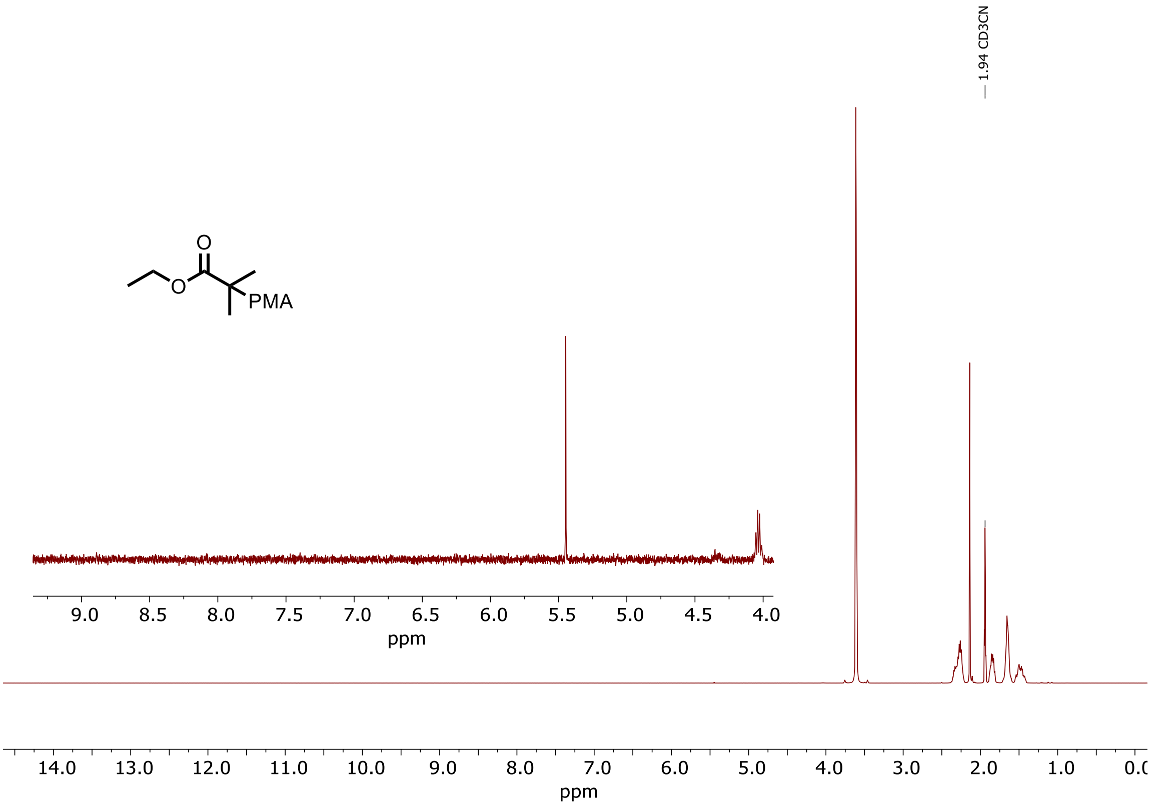

Spectrum S44. ^{1}H NMR (500 MHz, acetonitrile-d_3, 298 K, 32 scans) of polymer **PMA**.

5.24 Spectra of Furan

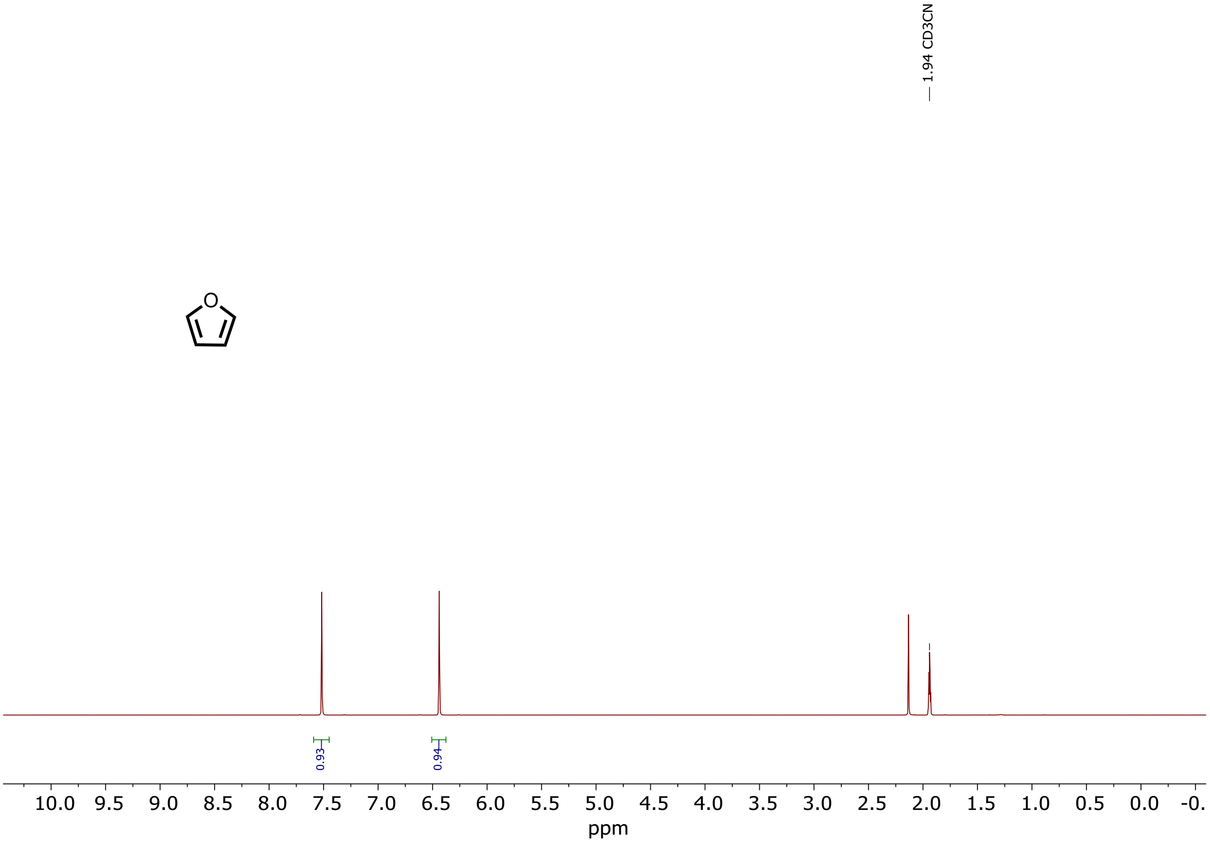

Spectrum S45. ^{1}H NMR (500 MHz, acetonitrile-d_3, 298 K, 16 scans) of furan reference.

6 Sonication Experiments

All experiments were carried out using the same sonication conditions (20 kHz, 13.0 W cm^{-2}, 1 s ON / 1 s OFF, 5 – 10 °C).

6.1 Standard Sonication Procedure

The desired polymer (45 mg) was dissolved in the dry acetonitrile (approx. 15 mL in total) and added to a modified Suslick cell. Nitrogen was gently bubbled through the solution as it was sonicated. The mixture was kept in an ice bath for the whole duration of the process and was sampled periodically (varied time intervals) over the course of 240 minutes of total sonication time. After 240 min of sonication time, the mixture was filtered through Whatman Puradisc 10 mm syringe filter with 0.2 µm PTFE membrane to remove metal impurities. After drying under high vacuum for an extended period of time (approximately 24 h), the polymer was washed with methanol (5 mL) before drying again.

6.2 Sonication Experiments Overview

The four mechanophore-centred polymers were subjected to mechanical activation following the standard sonication procedure. All samples were found to have fully cleaved after 240 minutes of sonication.

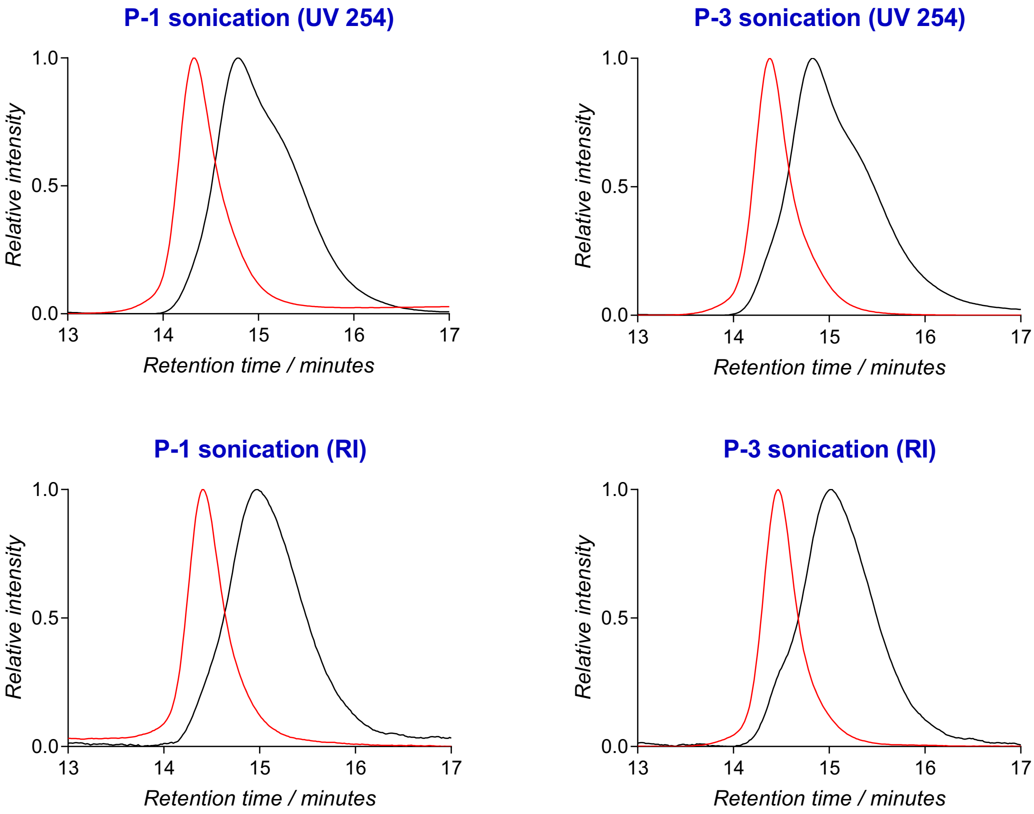

Figure S5. GPC chromatograms of pre- and post-sonication GPC traces (red and black, respectively) of -*trans* mechanophores.

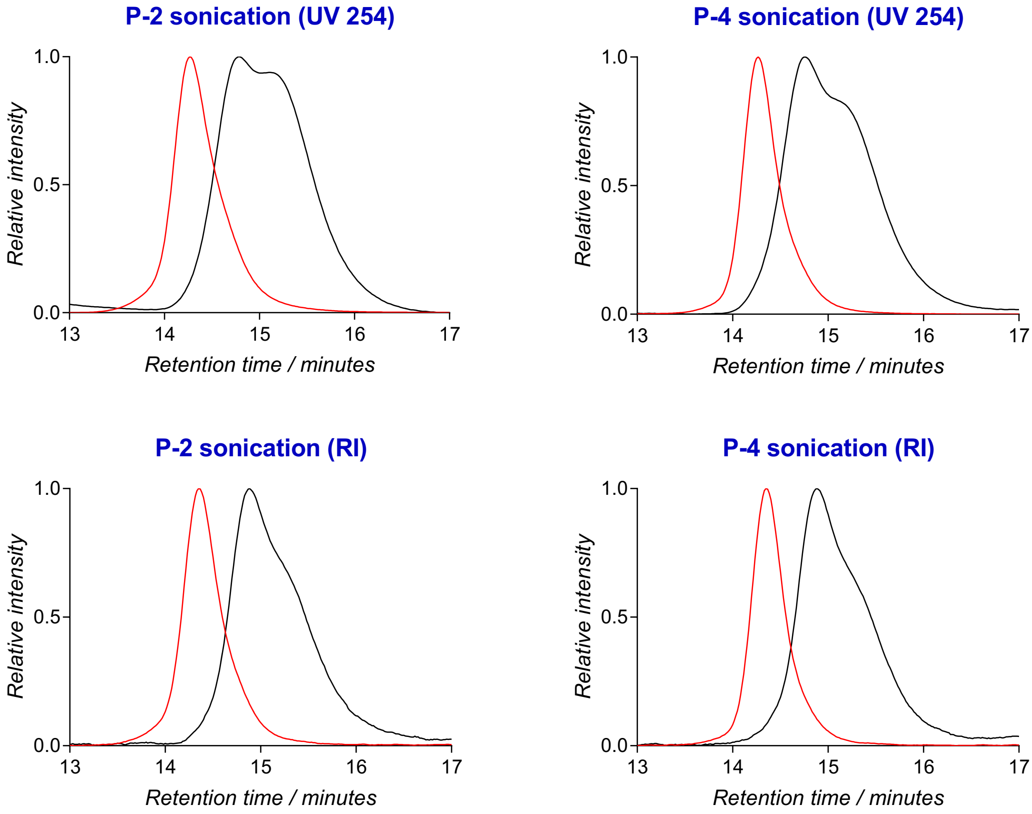

Figure S6. GPC chromatograms of pre- and post-sonication GPC traces (red and black, respectively) of -*cis* mechanophores.

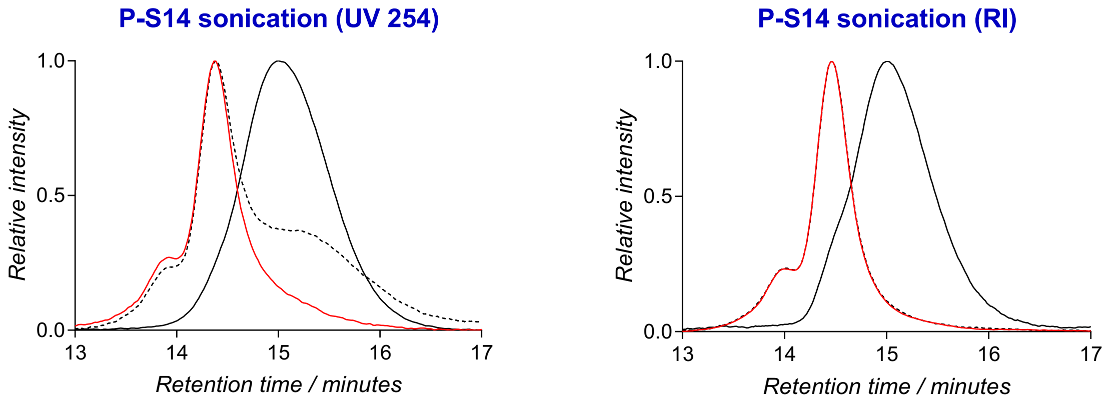

Figure S7. GPC chromatograms of pre- and post-sonication GPC traces (red and black, respectively) of **P-S14** reference compound. GPC of the heated sample is shown in black, dotted line.

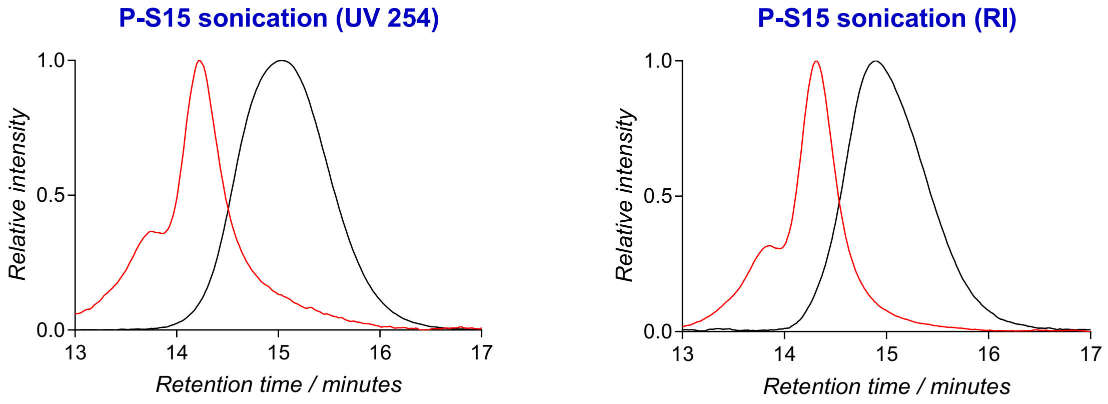

Figure S8. GPC chromatograms of pre- and post-sonication GPC traces (red and black, respectively) of **P-S15** reference compound.

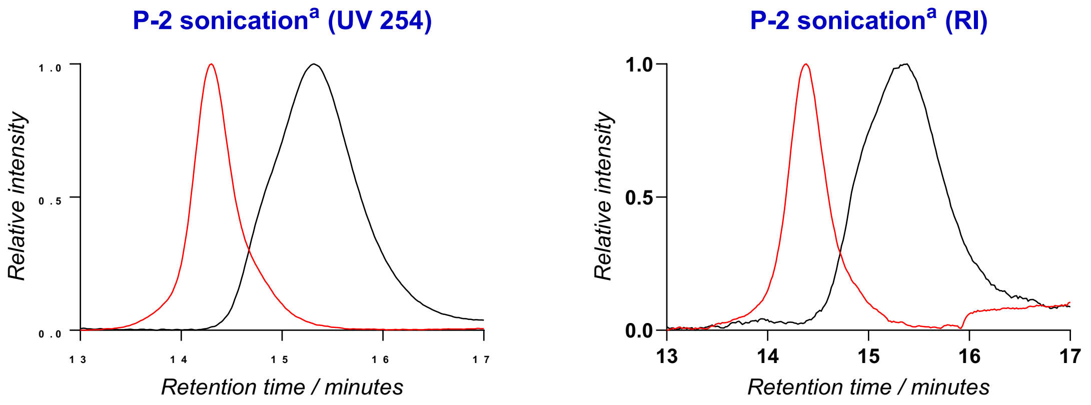

Figure S9. GPC chromatograms of pre- and post-sonication GPC traces (red and black, respectively) of polymer **P-2**. [a]Data referred to the sonication experiment of polymer **P-2** for furan observation.

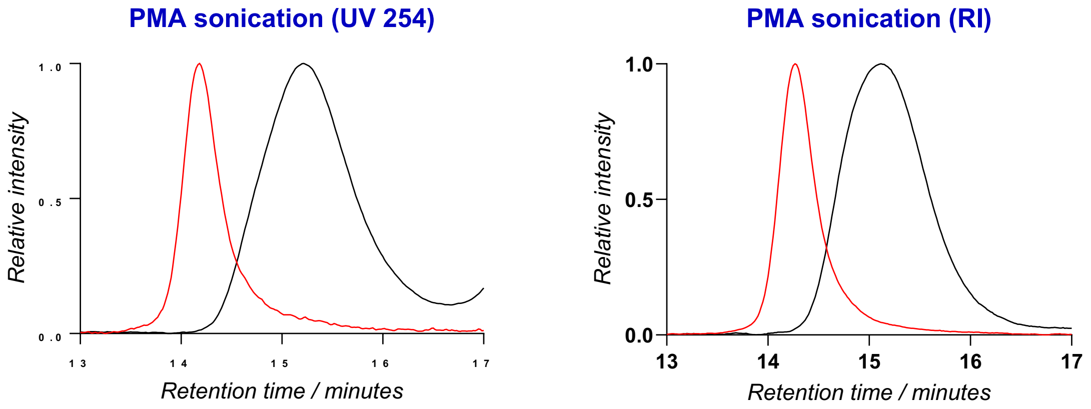

Figure S10. GPC chromatograms of pre- and post-sonication GPC traces (red and black, respectively) of polymer **PMA**.

6.3 Kinetic Investigation Overview

All samples were investigated according to standard sonication procedure. The sonication was sampled at constant time intervals across all samples. The sonications were sampled at sonication times of: 0, 5, 10, 20, 30, 45, 60, 90, 120, 180 and 240 minutes. Upon sonication, the average molecular weight of the sample tends toward 30 kDa (c) and the apparent rate constant (k*) can be derived with Nalepa's method (Table S2, Figure S9-S16).[S3]

Table S2. M_n and k* values for polymers **P1-P4**.

Polymer	Run	M_n at t = 0'	M_n at t = 240'	k* / $min^{-1}.kDa.10^5$	Average k*
	1	76 220	31 344	0.1421	
**P-1**	2	75 941	29 276	0.1204	0.1273
	3	76 215	31 097	0.1194	
	1	80 835	33 571	0.1262	
**P-2**	2	81 632	29 489	0.1408	0.1281
	3	82 157	33 149	0.1173	
	1	74 595	30 688	0.1009	
**P-3**	2	73 732	32 533	0.0925	0.0999
	3	76 872	32 047	0.1064	
	1	87 063	35 557	0.1189	
**P-4**	2	87 466	30 438	0.1340	0.1249
	3	87 038	32 759	0.1218	

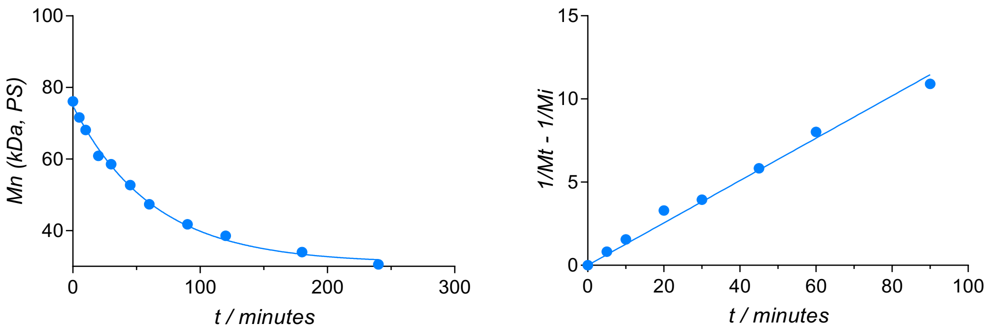

Figure S11. Kinetics of **P-1**, showing M_n decay over the course of the sonication as well as k* determination (left and right, respectively).

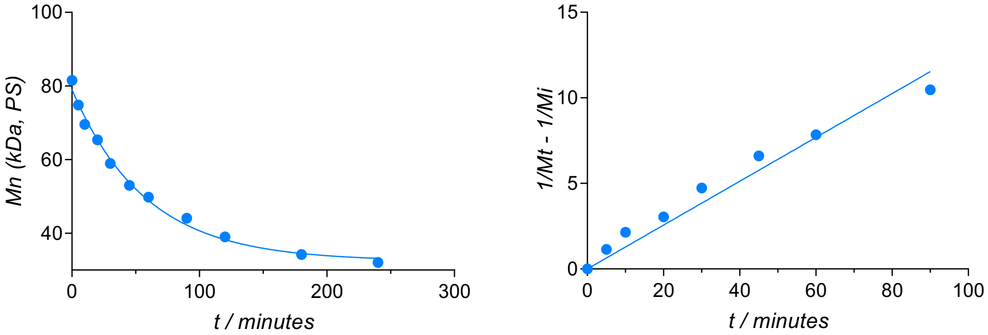

Figure S12. Kinetics of **P-2**, showing M_n decay over the course of the sonication as well as k* determination (left and right, respectively).

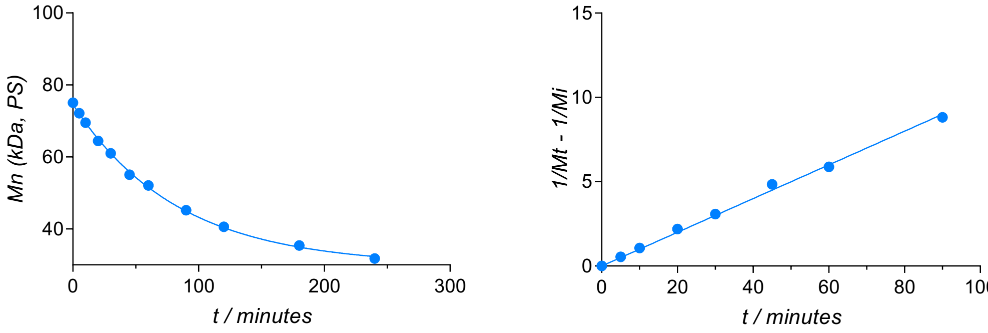

Figure S13. Kinetics of **P-3**, showing M_n decay over the course of the sonication as well as k* determination (left and right, respectively).

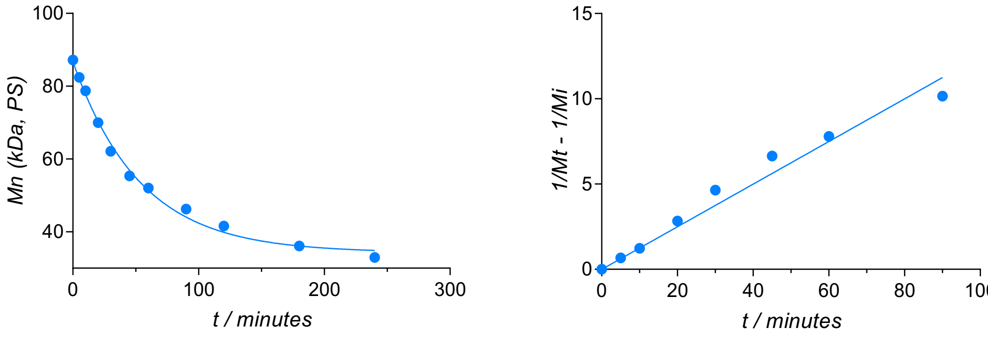

Figure S14. Kinetics of **P-4**, showing M_n decay over the course of the sonication as well as k* determination (left and right, respectively

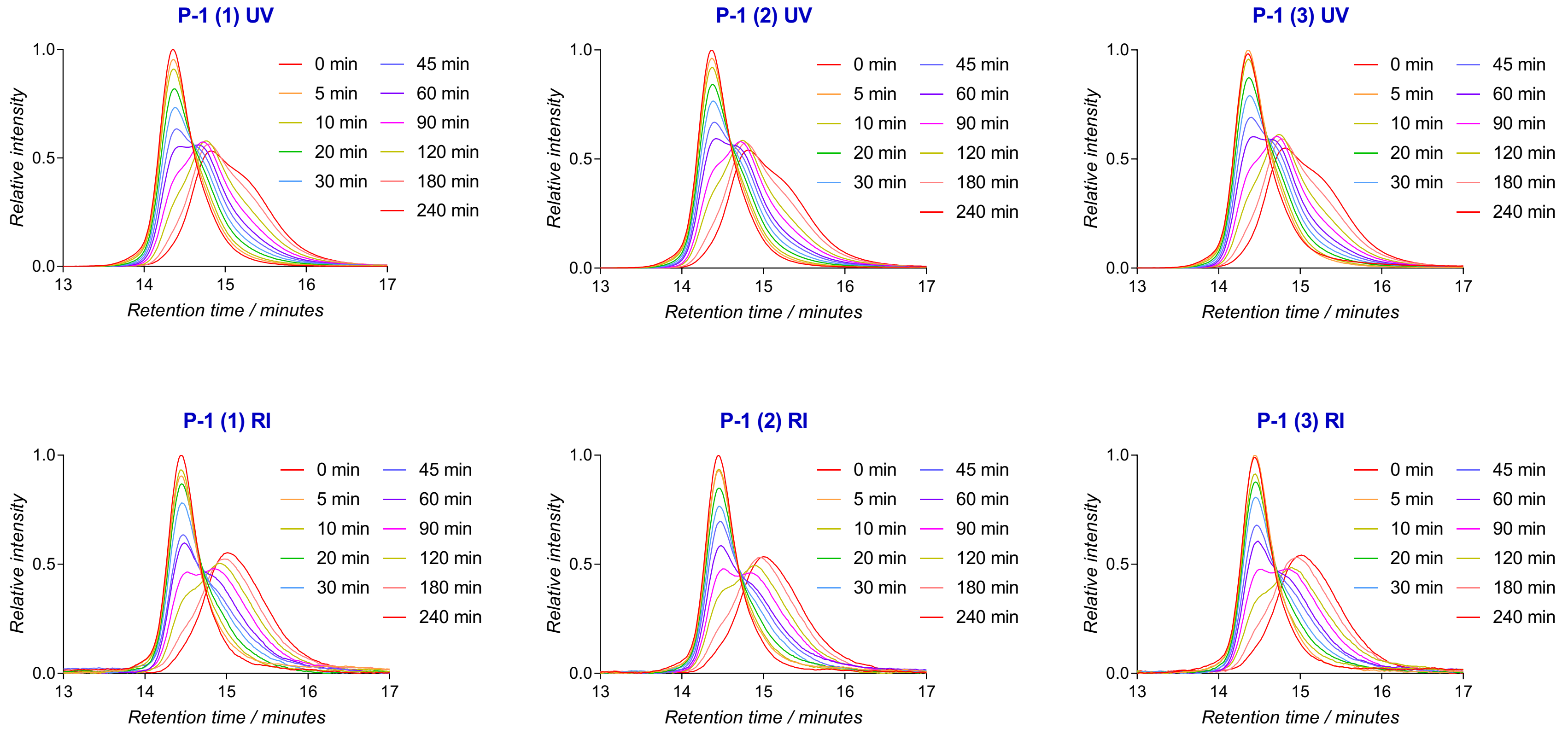

Figure S15. Kinetics of **P-1** carried out in triplicate, showing area-normalised response traces for UV 254 nm and RI detectors (top and bottom, respectively).

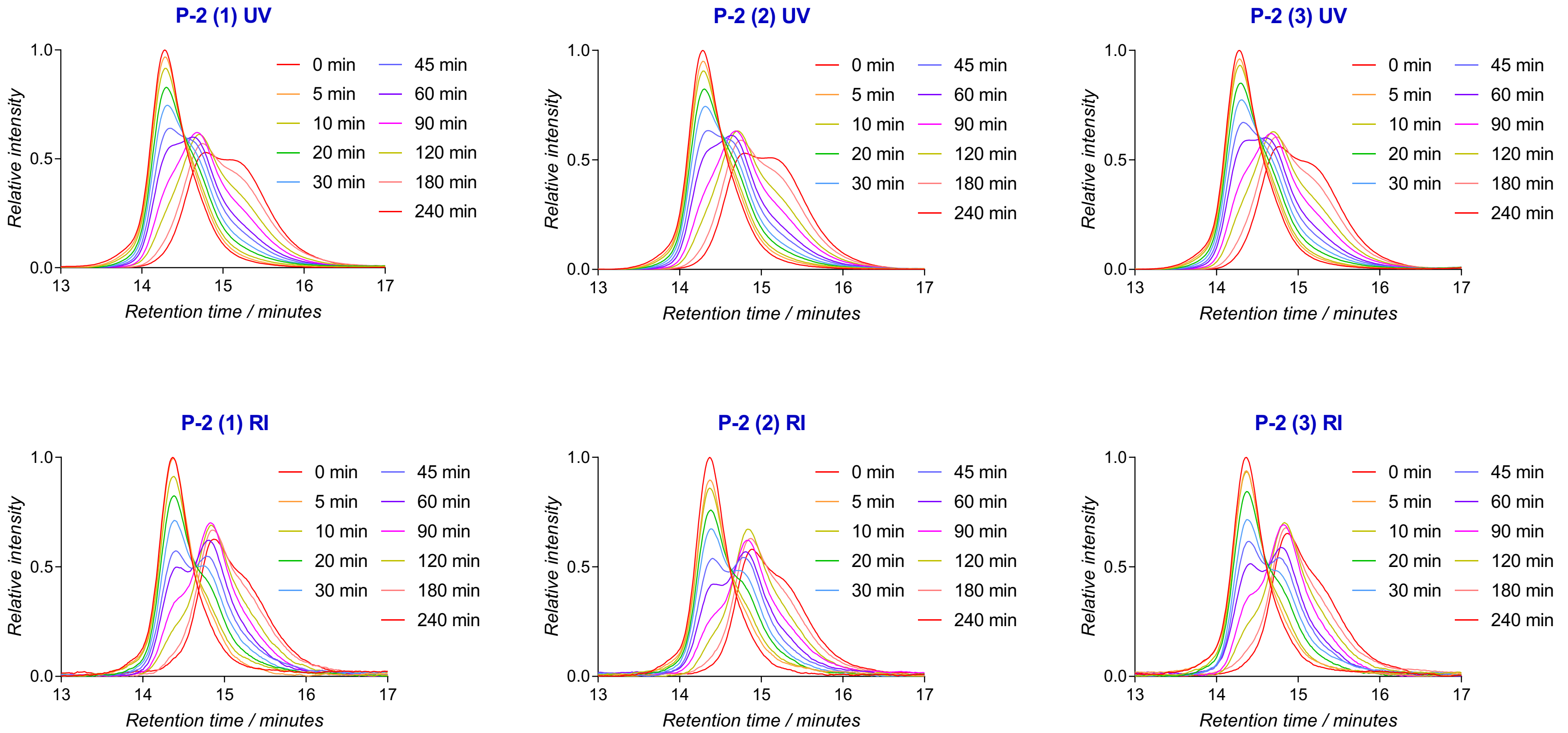

Figure S16. Kinetics of **P-2** carried out in triplicate, showing area-normalised response traces for UV 254 nm and RI detectors (top and bottom, respectively).

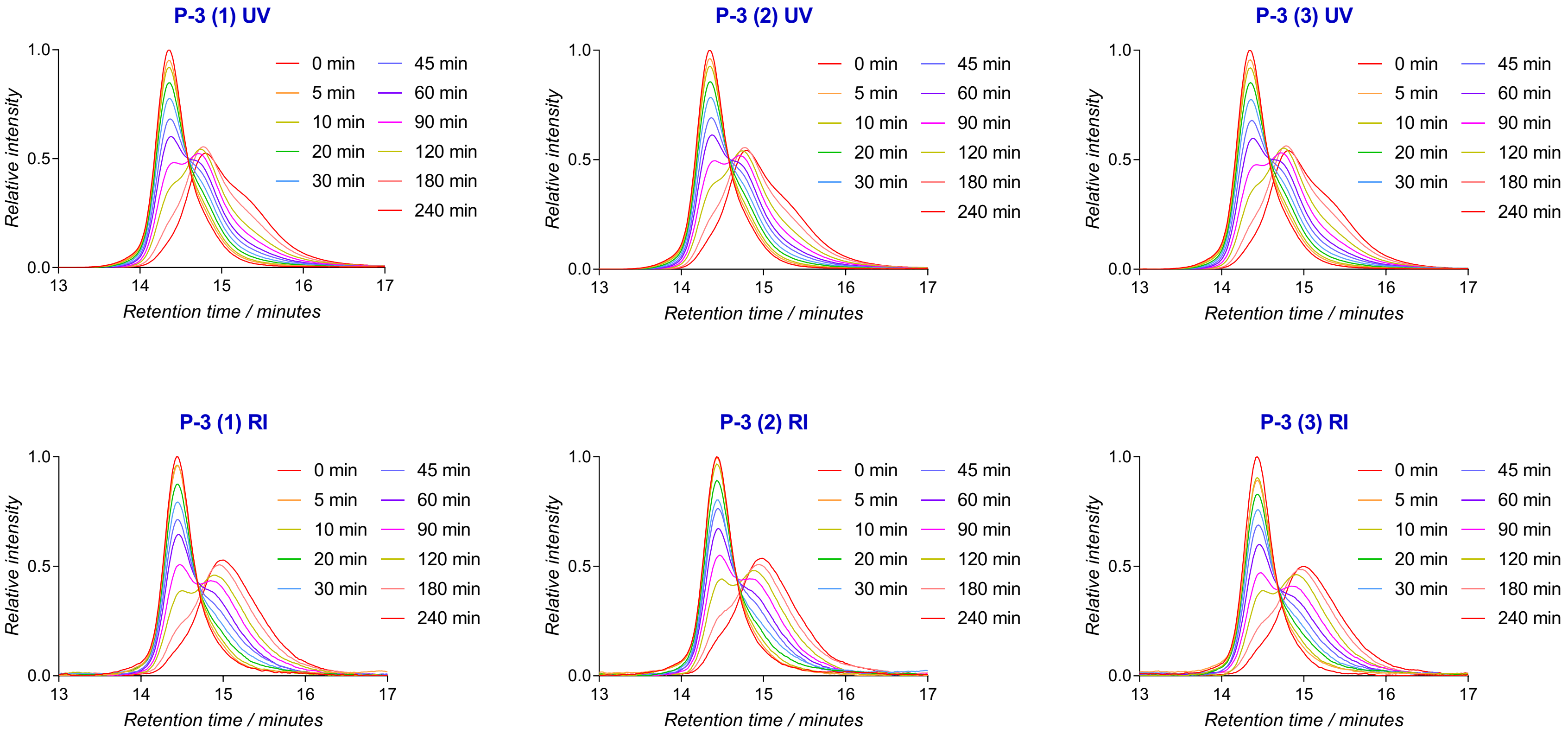

Figure S17. Kinetics of **P-3** carried out in triplicate, showing area-normalised response traces for UV 254 nm and RI detectors (top and bottom, respectively).

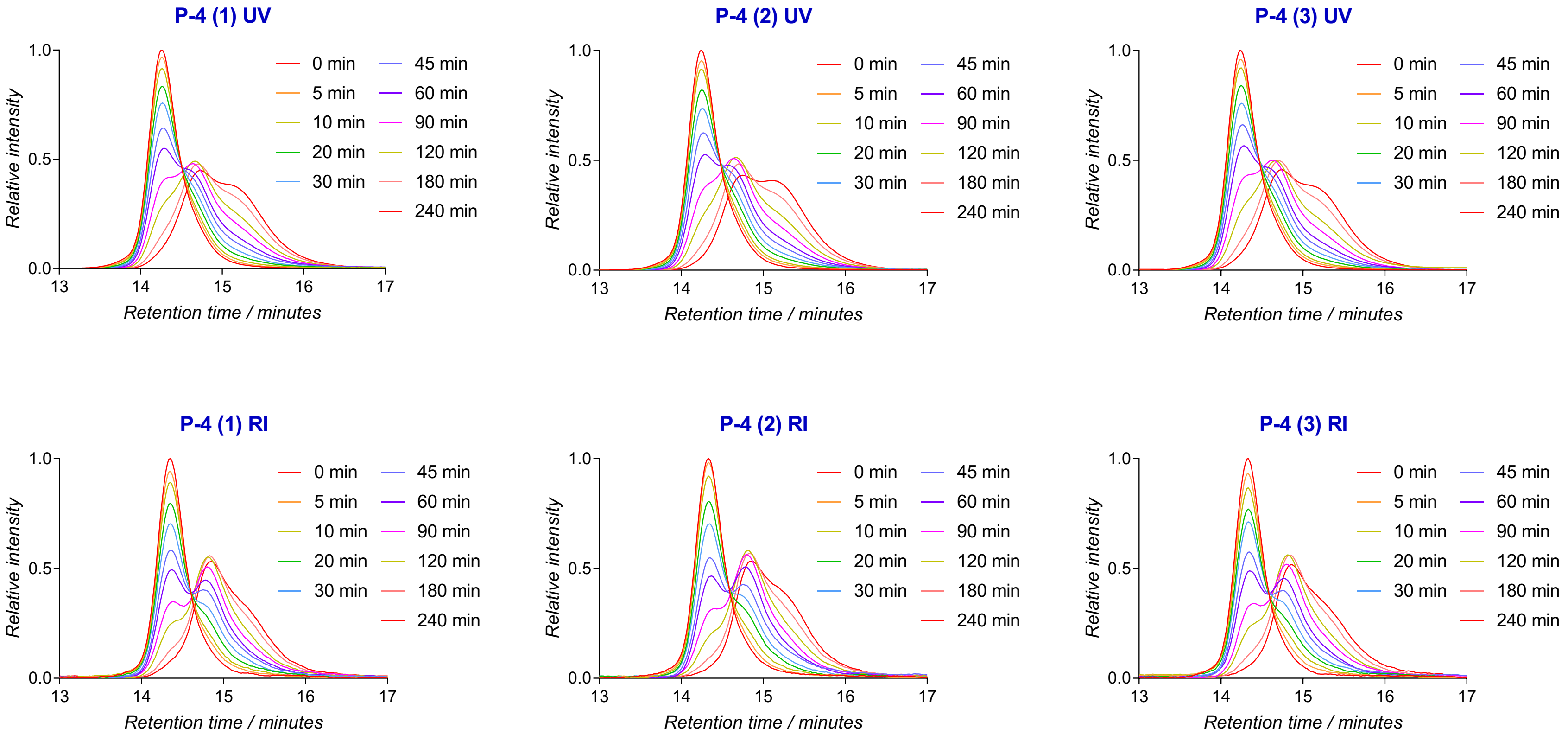

Figure S18. Kinetics of **P-4** carried out in triplicate, showing area-normalised response traces for UV 254 nm and RI detectors (top and bottom, respectively)

6.4 Sonication Experiment of Polymer P-1

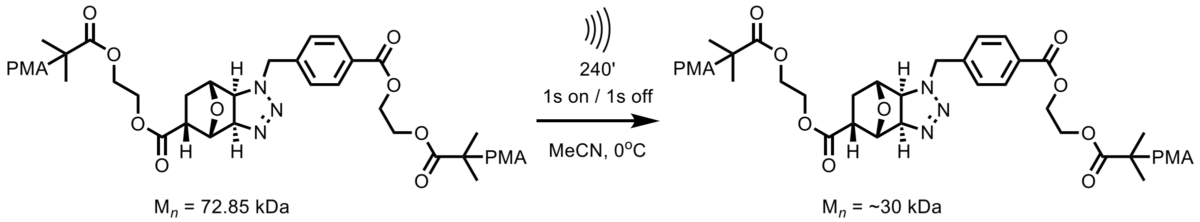

Scheme S5. Sonication of polymer **P-1** (*endo-exo-trans*).

Complete scission of polymer **P-1** was confirmed by GPC. However, NMR analysis of the post-sonication solid, it was clear that the footprint of the mechanophore within polymer **P-1** was not affected by the sonication (Scheme S5). Application of the force led to random polymer backbone scission within PMA chains attached to the mechanophore, as observed by the GPC of the post-sonication mixture (Scheme S5). The results indicate that *endo-exo-trans* geometry is not capable of force transduction leading to the retro-cycloaddition products (Figure S19).

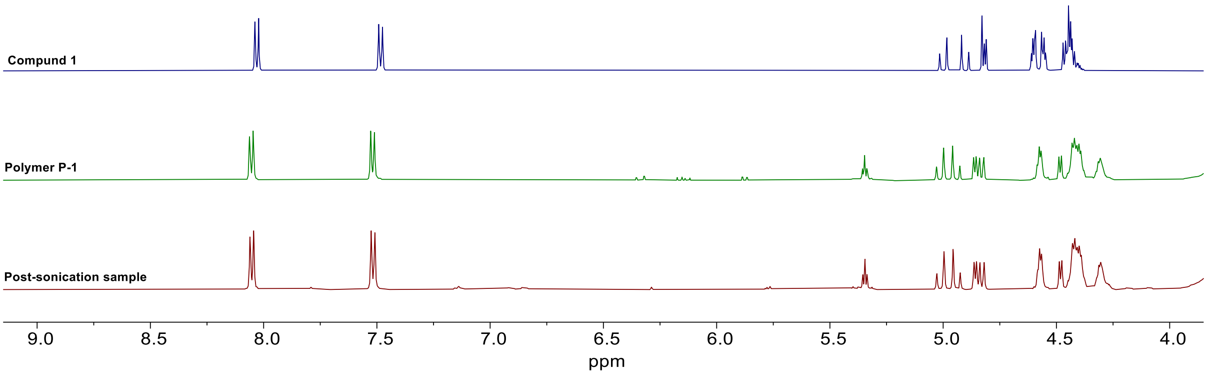

Figure S19. Partial ^{1}H NMR stack (500 MHz, acetone-d$_6$, 1024 scans) of **1**, **P-1** and post-sonication sample of **P-1** (top to bottom, respectively).

6.5 Sonication Experiment of Polymer P-2

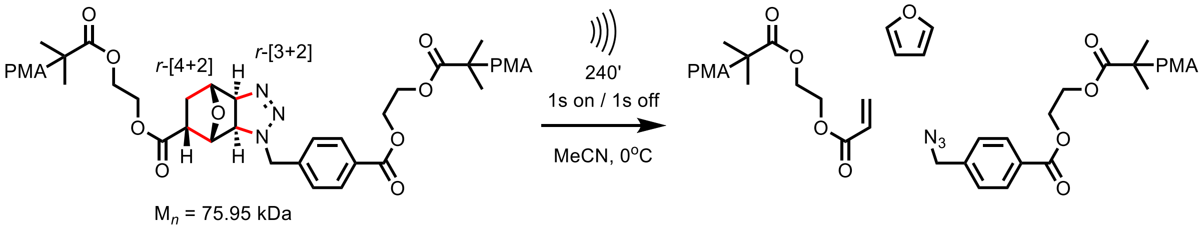

Scheme S6. Sonication of polymer **P-2** (*endo-exo-cis*). Scissile bonds were marked in red. Main products are shown.

Complete scission of polymer **P-2** was confirmed by GPC. From the NMR analysis of the post-sonication solid, it was clear that the mechanophore within polymer **P-2** had undergone a chemical change. The geometry of the adduct (*endo-exo-cis*) was found to be mechanically susceptible to undergo a mechanochemically triggered process to yield retro-[3+2] and retro-[4+2][3+2] products; to release free furan, along polymer attached moieties containing acrylate and azido-benzyl derivative (Scheme S6). Indicative of the generation of products of the retro-cycloaddition of the triazoline moiety (retro-[3+2]) was the

appearance of the sharp singlet at 4.59 ppm in the ^{1}H NMR of the post-sonication polymer (Figure S20), which corresponds to the methylene adjacent to the azido group in the reference polymer **P-S5**, as well as shift of the aromatic peaks (blue dotted lines). There have been new signals appearing in the region between 5.8 and 6.6 ppm in the post sonication sample, which was attributed to the trace of the **P-S14** present in the mixture (black dotted lines) as the product of the retro-[3+2] along the product from retro-[4+2][3+2] whose reference was obtained by heating a **P-S14** sample to 70°C for 24h in toluene (red dotted lines).

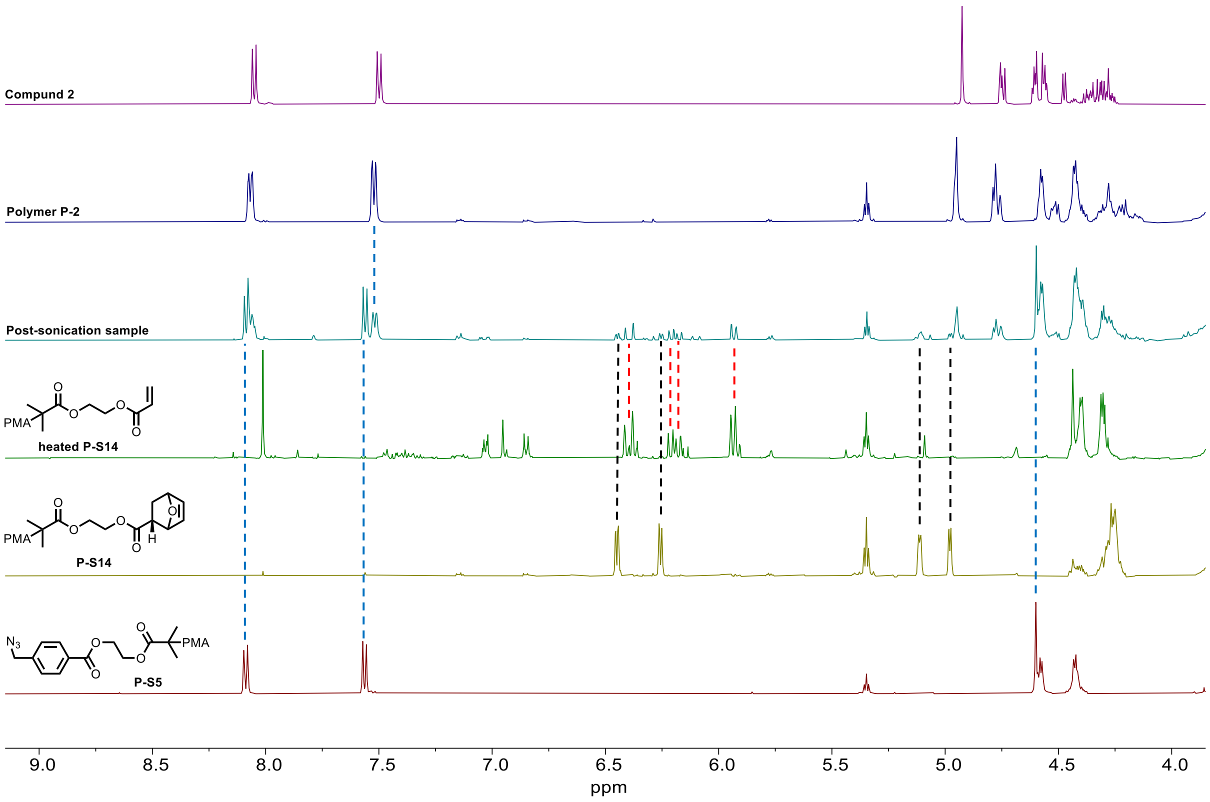

Figure S20. Partial ^{1}H NMR stack (500 MHz, acetone-d_6, 1024 scans) of **2**, **P-2**, post-sonication sample of **P-2**, heated **P-S14**, **P-S14** and **P-S5** (top to bottom, respectively).

Polymer **P-2** undergoes partial transformation where approximately 50% of the mechanophores are activated (determined from the azide signals, see manuscript) by: retro-[4+2][3+2] (30%), retro-[3+2] (14%), unidentified pathway (remaining 6%).

6.6 Sonication Experiment of Polymer P-3

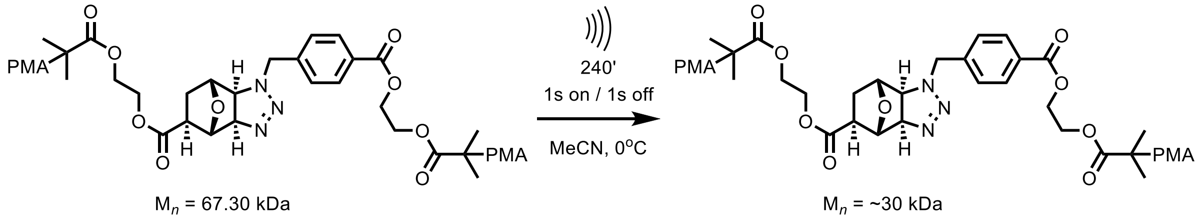

Scheme S7. Sonication of polymer **P-3** (*exo-exo-trans*).

Complete scission of polymer **P-3** was confirmed by GPC. However, NMR analysis of the post-sonication solid, it was clear that the footprint of the mechanophore within polymer **P-3** was not affected by the course of the sonication (Scheme S7). Application of the force led to random polymer backbone scission within PMA chains attached to the mechanophore, as observed by the GPC of the post-sonication mixture. The results indicate that *exo-exo-trans* geometry is not capable of force transduction leading to the retro-cycloaddition products (Figure S21).

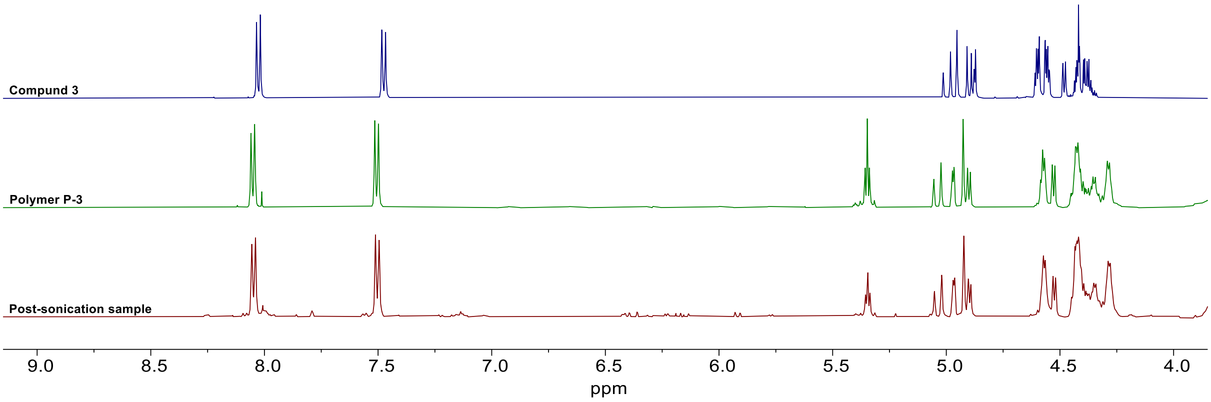

Figure S21. Partial ^{1}H NMR stack (500 MHz, acetone-d$_6$, 1024 scans) of **3**, **P-3** and post-sonication sample of **P-3** (top to bottom, respectively).

6.7 Sonication Experiment of Polymer P-4

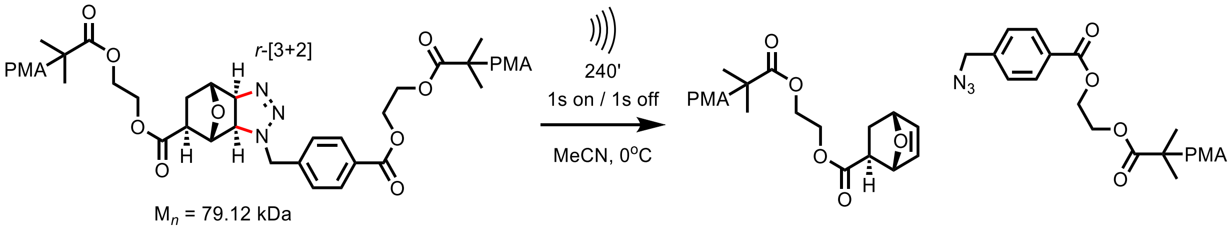

Scheme S8. Sonication of polymer **P-4** (*exo-exo-cis*).

Complete scission of polymer **P-4** was confirmed by GPC. From the NMR analysis of the post-sonication solid, it was clear that the mechanophore within polymer *exo-exo-cis* had undergone a chemical change. The geometry of the adduct (*endo-exo-cis*) was found in our study to be mechanically susceptible to undergo a mechanochemical process to yield retro-[3+2] products, to the species as in reference compound **P-S15** and azido-benzyl derivative (Scheme S8). Indicative of the generation of products of the retro-cycloaddition of the

triazoline moiety (retro-[3+2]) was the appearance of the sharp singlet at 4.59 ppm which corresponds to the methylene adjacent to the azido group in the reference polymer **P-S5**, as well as shift of the aromatic peaks (blue dotted lines). There have been new signals appearing at 6.4 ppm in the post-sonication sample, which was attributed to the **P-S15** present in the mixture (black dotted lines).

Polymer **P-4** undergoes partial transformation in the mechanochemical activation where approximately 50% of the mechanophore yields the azido-methylene containing aromatic groups as per reference **P-S5** in the mechanical activation. The integration of the aromatic signals matches well with signal at 6.4 ppm (Figure S22).

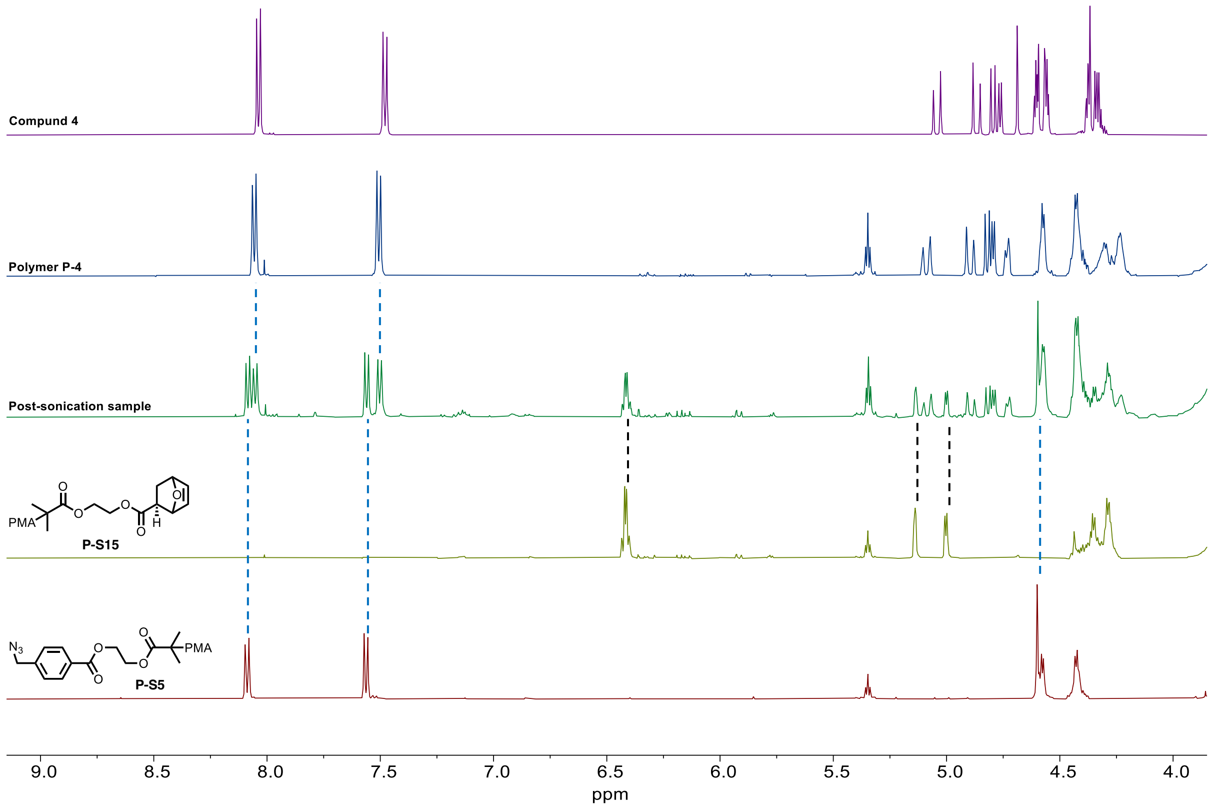

Figure S22. Partial ^{1}H NMR stack (500 MHz, acetone-d_6, 1024 scans) of **4**, **P-4**, post-sonication sample of **P-4**, **P-S15** and **P-S5** (top to bottom, respectively).

6.8 Sonication Experiment of Polymer P-S14

Complete scission of polymer **P-S14** was confirmed by GPC after 240 min of sonication. No changes were observed to the oxanorbornene unit as confirmed by NMR (Figure S27).

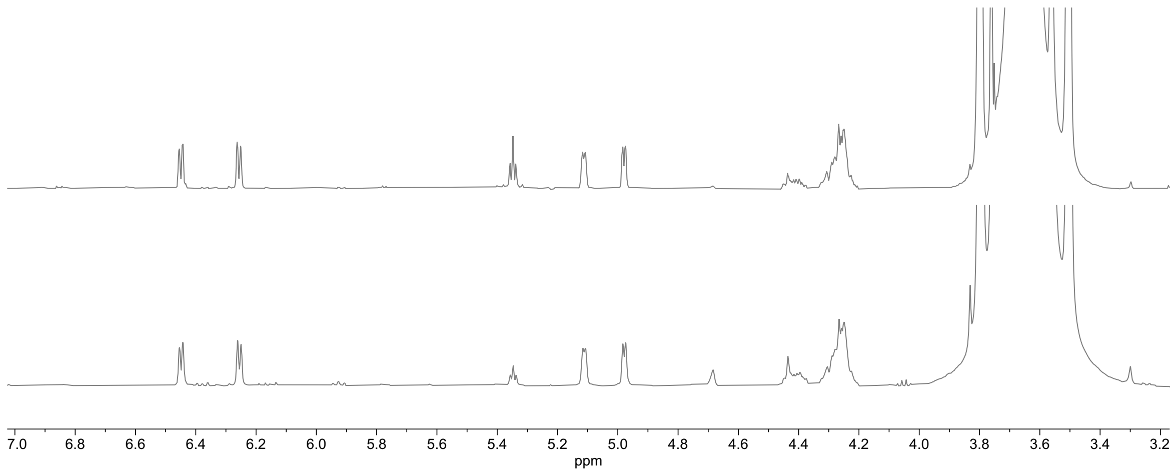

Figure S23. Partial ^{1}H NMR stack (500 MHz, acetone-d_6, 1024 scans) of **P-S14** pre- (top) and post- (bottom) sonication.

6.9 Sonication Experiment of Polymer P-S15

Complete scission of polymer **P-S15** was confirmed by GPC after 240 min of sonication. No changes were observed to the oxanorbornene unit as confirmed by NMR (Figure S24).

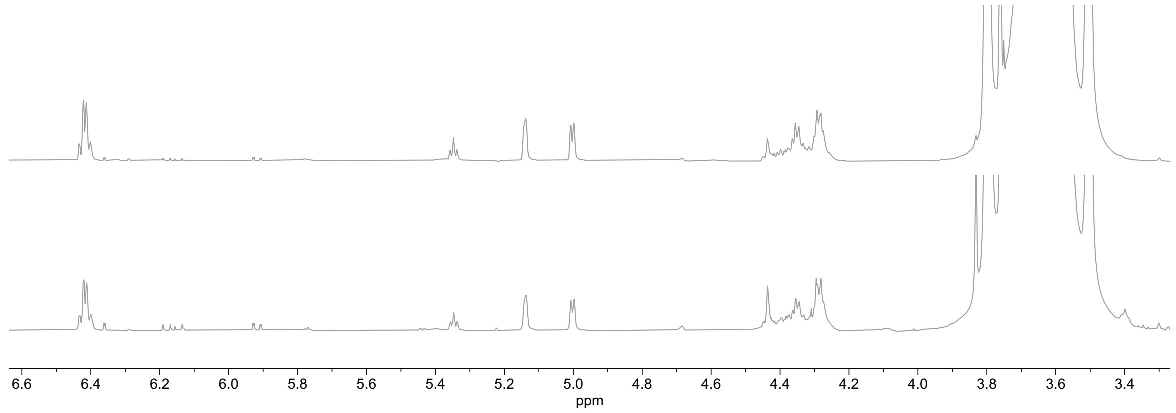

Figure S24. Partial ^{1}H NMR stack (500 MHz, acetone-d_6, 1024 scans) of **P-S15** pre- (top) and post- (bottom) sonication.

6.10 Sonication Experiment of Polymer P-2 for Furan release

Sonication was performed using a modified version of the standard procedure: **P2** (70 mg) was dissolved in acetonitrile-*d₃* (approximately 8 mL) and added to a modified Suslick cell and degassed with nitrogen for 10 minutes. No gas was purged through the solution as it was sonicated. The mixture was kept in ice bath for the whole duration of the process over the course of 450 minutes of total sonication time. After 450 min of sonication time, an aliquot of 0.6 mL was taken and filtered through Whatman Puradisc 10 mm syring filter with 0.2 µm PTFE membrane to remove metal impurities. The filtered solution was directly used for ^{1}H NMR analysis.

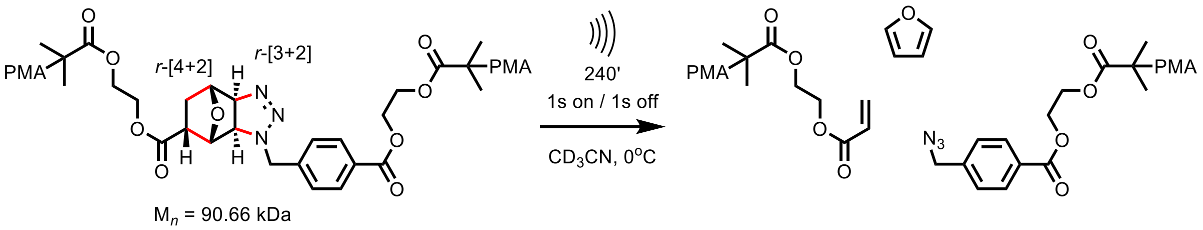

Scheme S9. Sonication of polymer **P-2** (*endo-exo-cis*). Scissile bonds were marked in red. Main products are shown.

From the NMR analysis of the post-sonication sample, it was clear that the mechanophore within polymer **P-2** had undergone a chemical change. The geometry of the adduct (*endo-exo-cis*) was found to be mechanically susceptible to undergo a mechanochemically triggered process to yield retro-[3+2] and retro-[4+2][3+2] products; to release free furan, along polymer attached moieties containing acrylate and azido-benzyl derivative (Scheme S9). Indicative of the generation of furan was the two triplets at 7.51 ppm and 6.44 ppm in the crude ^{1}H NMR of the post-sonication sample[a] (Figure S25, light blue dotted lines). There have been new signals appearing in the region between 5.8 to 6.8 ppm in the post-sonication sample, which was attributed to the methyl acrylate generated throughout the sonication (see section 6.11).

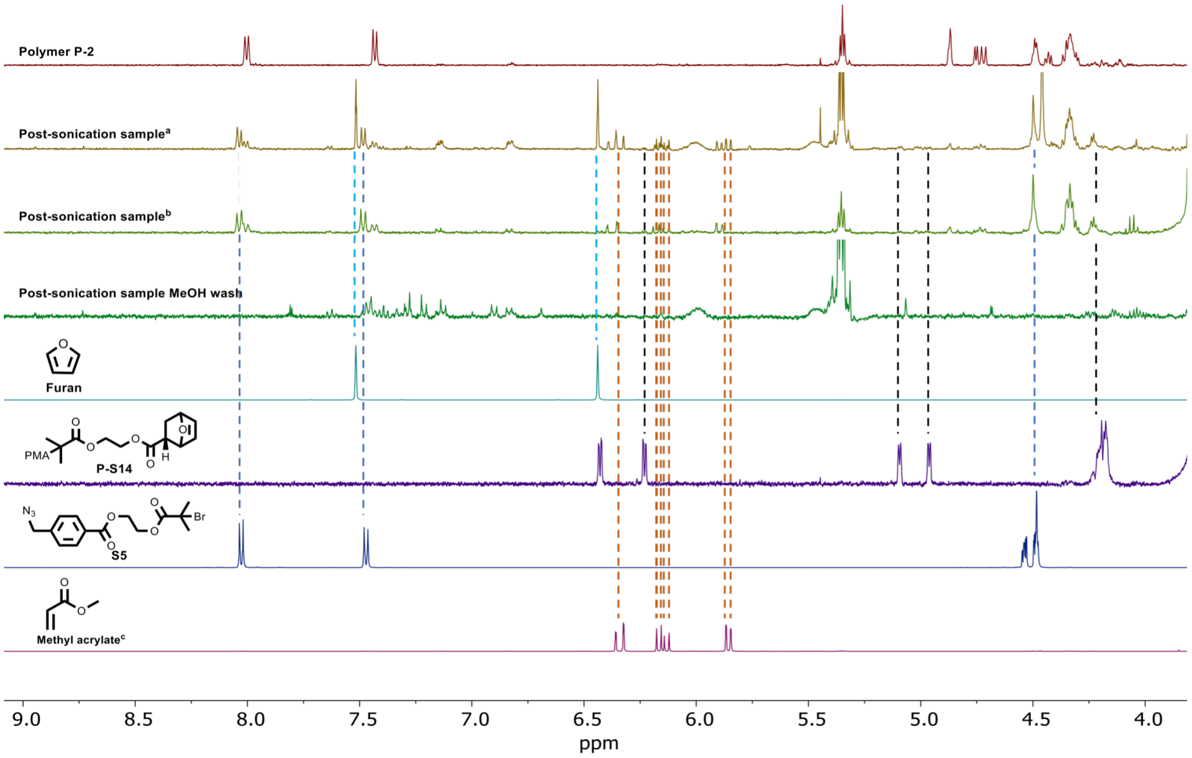

Figure S25. Partial ^{1}H NMR stack (500 MHz, acetonitrile-d_3, 256 scans) of **P-2**, post-sonication sample[a] of **P-2** without solvent removal, post-sonication sample[b] of **P-2** after methanol wash, **P-S14**, **P-S15**, methyl acrylate[c] spiked post-sonication sample[a] of **P-2** (top to bottom, respectively). [a] Post-sonication sample of **P-2** with NMR spectrum recorded after discarding metal particulates without solvent removal. [b] Post-sonication sample of **P-2** after methanol wash. [c]0.5µL methyl acrylate was spiked into 10 mg of post-sonication sample of **P2.**

6.11 Sonication Experiment of PMA

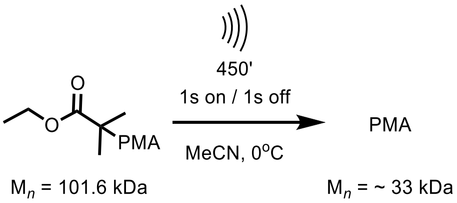

Scheme S10. Sonication of **PMA**.

Sonication was performed in acetonitrile-d_3 using the procedure described in section 6.10. Complete scission of PMA was confirmed by GPC. From the NMR analysis of the post-sonication samples, it was clear that methyl acrylate was generated upon scission of PMA chains. The disappearance of the methyl acrylate peaks of post-sonication samples after methanol wash indicates that the peaks at 5.7 to 6.4 are small molecules that are not attached to the polymer chain. The methyl acrylate generated in the previous sonication of **P2** (Figure S26) was attributed to the presence of PMA not the mechanophore.

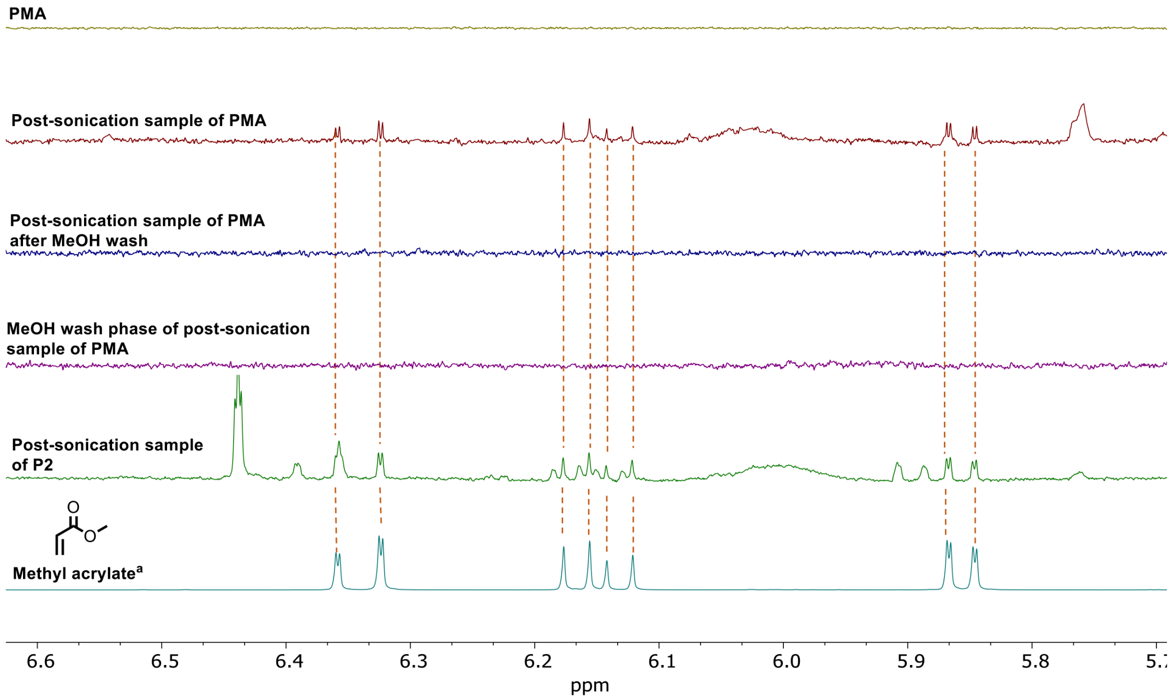

Figure S26. Partial ^{1}H NMR stack (500 MHz, acetonitrile-d$_3$, 256 scans) of **PMA**, post-sonication sample of **PMA** without solvent removal, post-sonication sample of **PMA** after methanol wash, post-sonication methanol wash phase, post-sonication sample of **P2**, methyl acrylate (top to bottom, respectively). [a] 0.5µL methyl acrylate was spiked into 10 mg of post-sonication sample of **P2.**

6.12 Post-Sonication NMR Spectra

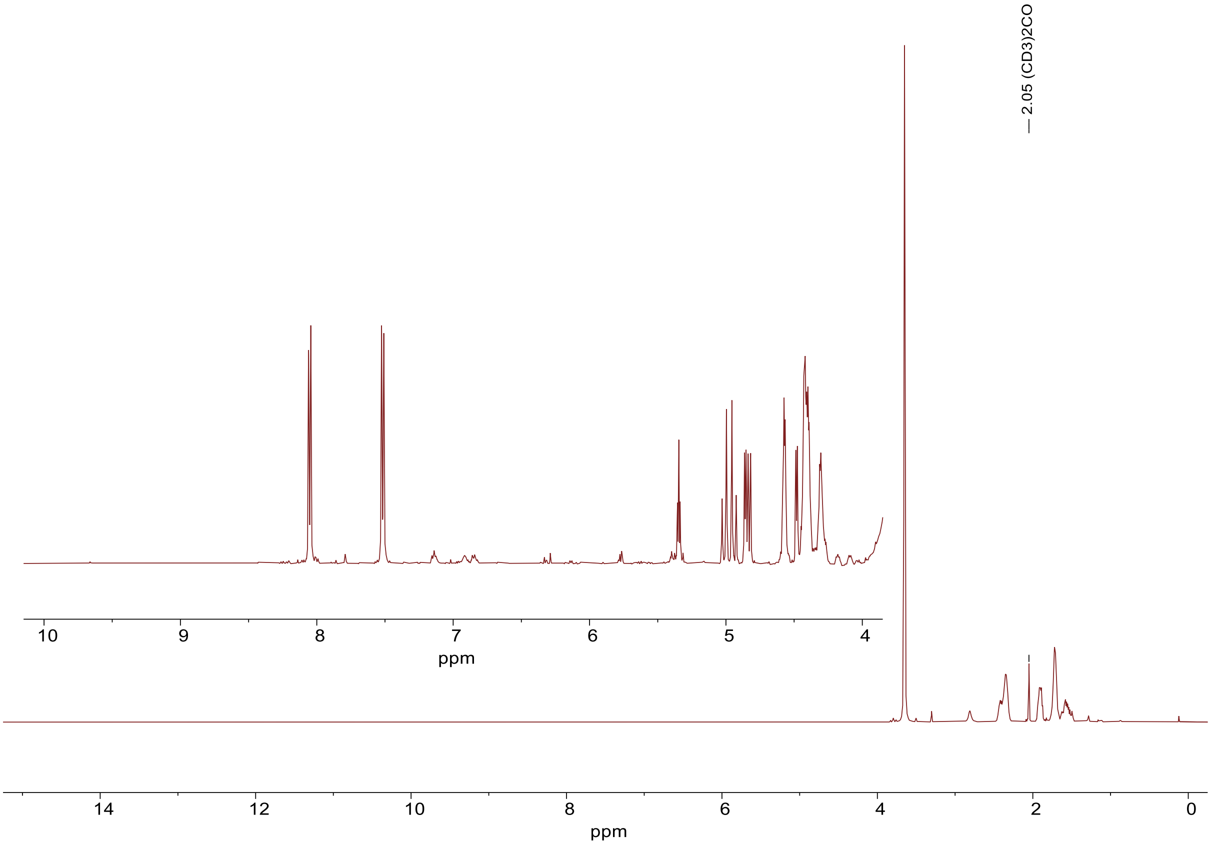

Spectrum S46. ^{1}H NMR (500 MHz, acetone-d6, 298 K, 1024 scans) of **P-1** after 240 min of sonication in acetonitrile.

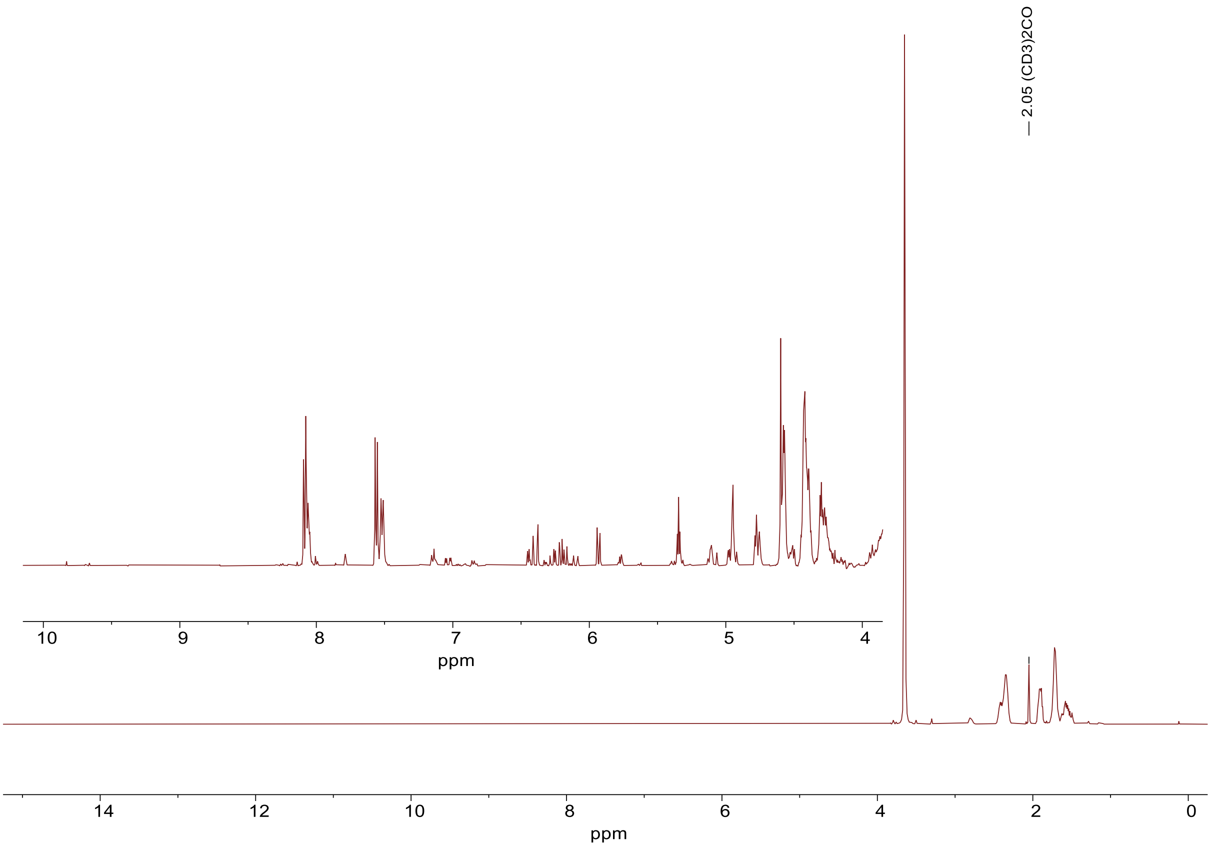

Spectrum S47. ^{1}H NMR (500 MHz, acetone-d6, 298 K, 1024 scans) of **P-2** after 240 min of sonication in acetonitrile.

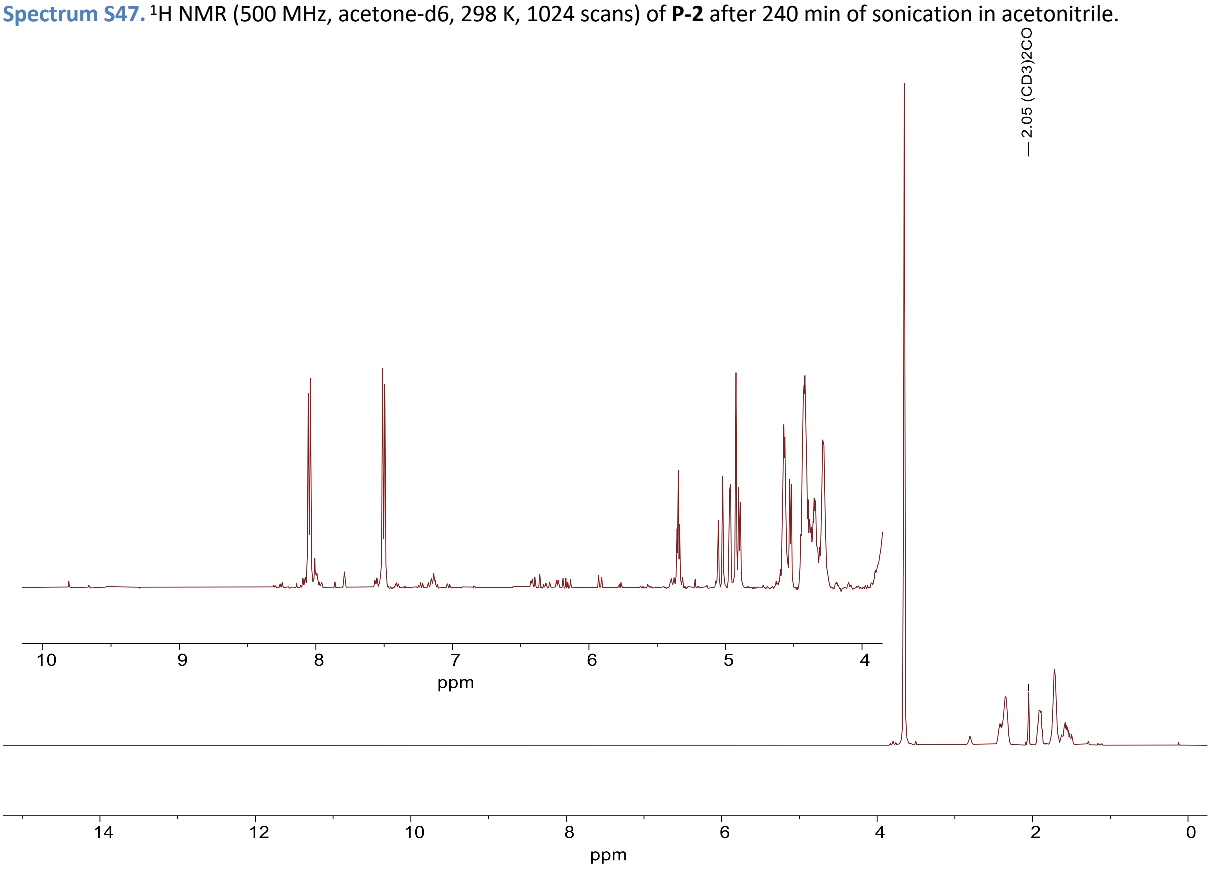

Spectrum S48. ^{1}H NMR (500 MHz, acetone-d6, 298 K, 1024 scans) of **P-3** after 240 min of sonication in acetonitrile.

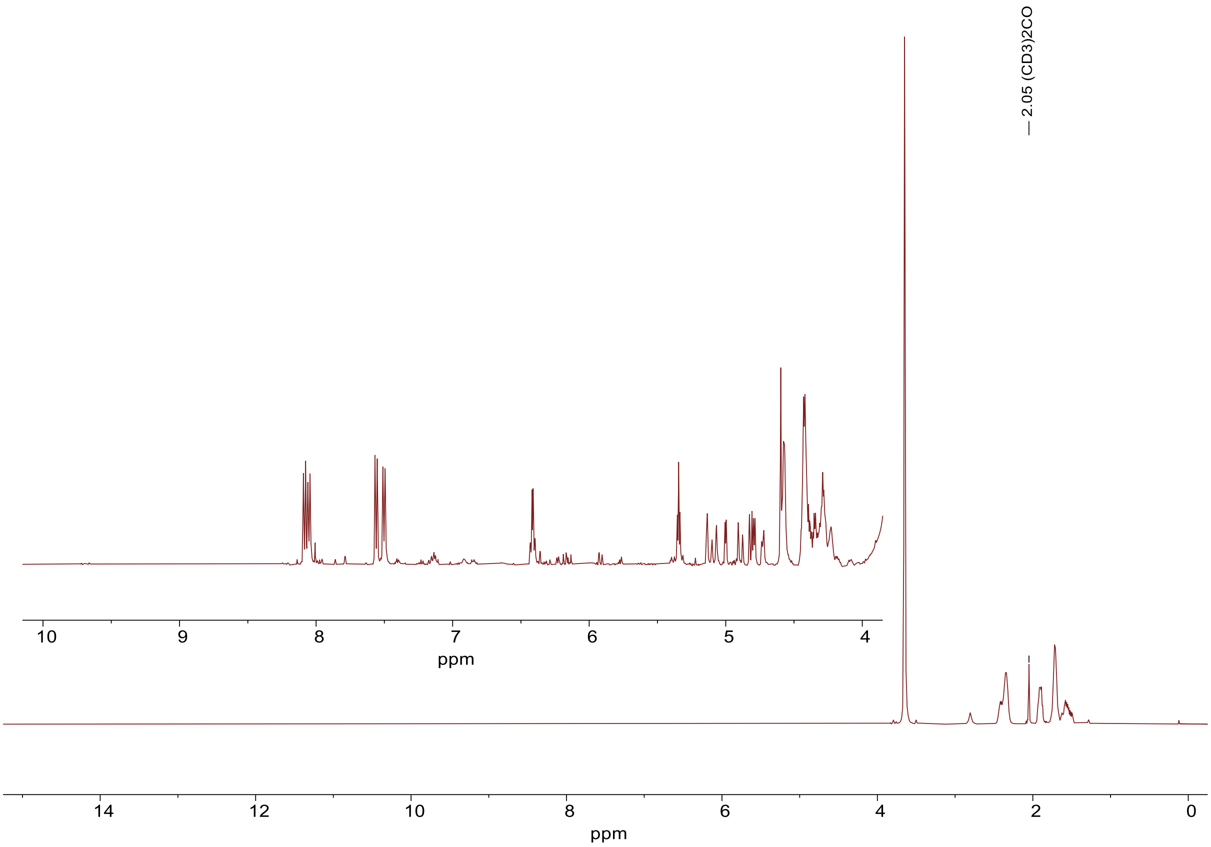

Spectrum S49. ^{1}H NMR (500 MHz, acetone-d6, 298 K, 1024 scans) of **P-4** after 240 min of sonication in acetonitrile.

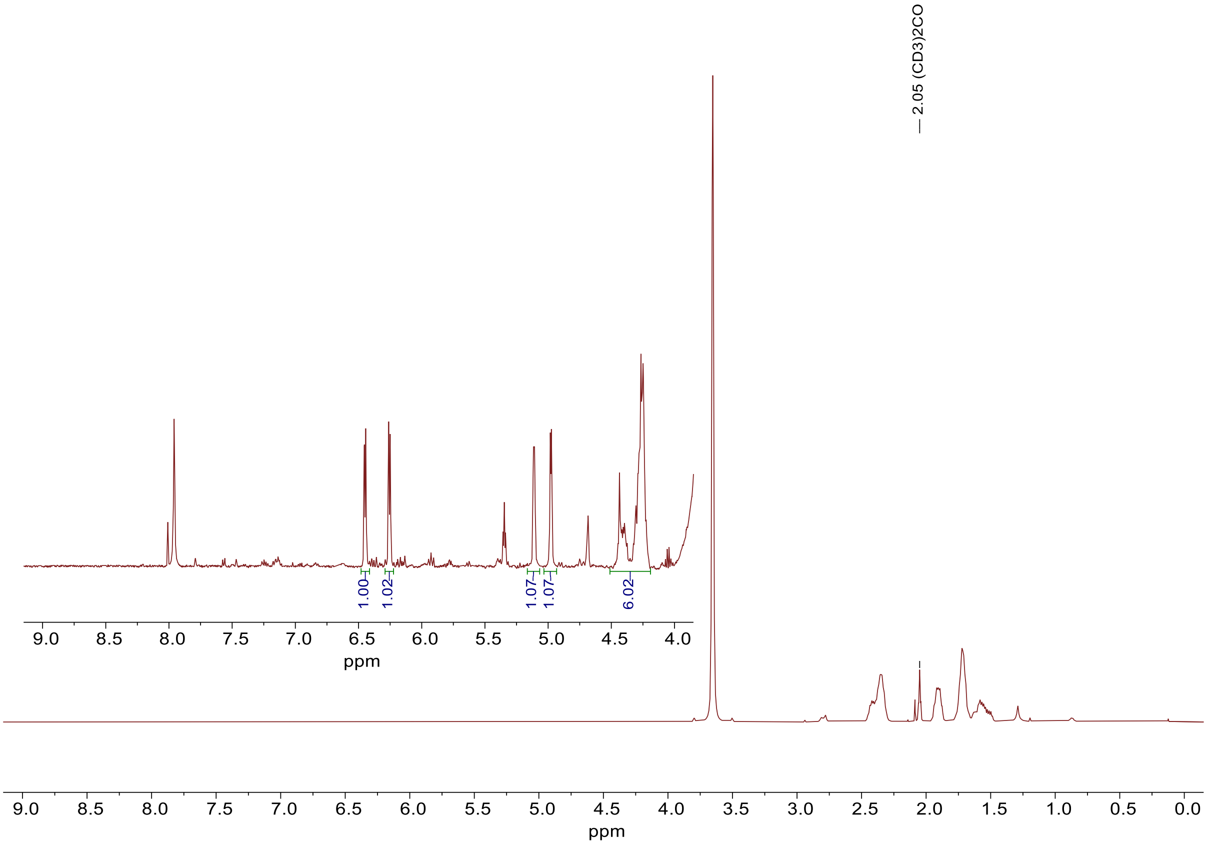

Spectrum S50. ^{1}H NMR (500 MHz, acetone-d6, 298 K, 1024 scans) of **P-S14** after 240 min of sonication in acetonitrile.

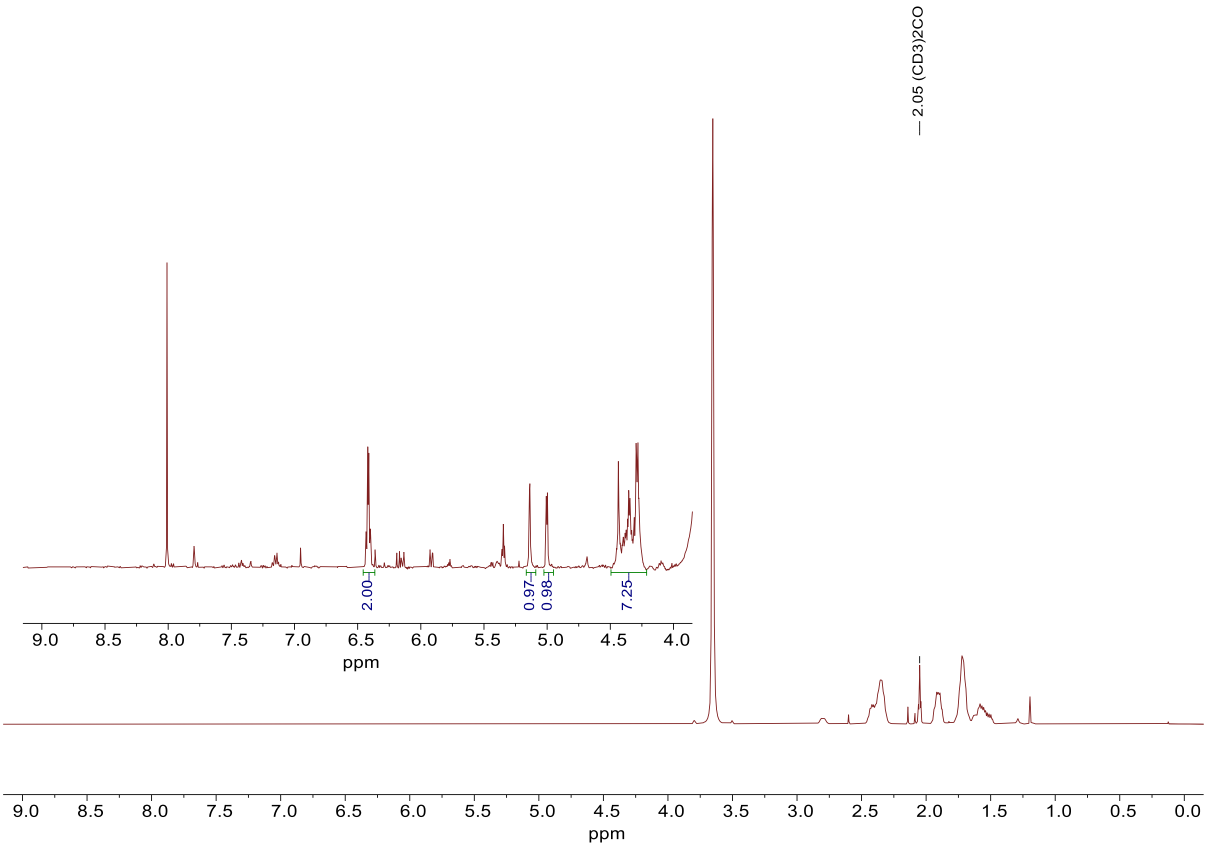

Spectrum S51. ^{1}H NMR (500 MHz, acetone-d6, 298 K, 1024 scans) of **P-S15** after 240 min of sonication in acetonitrile.

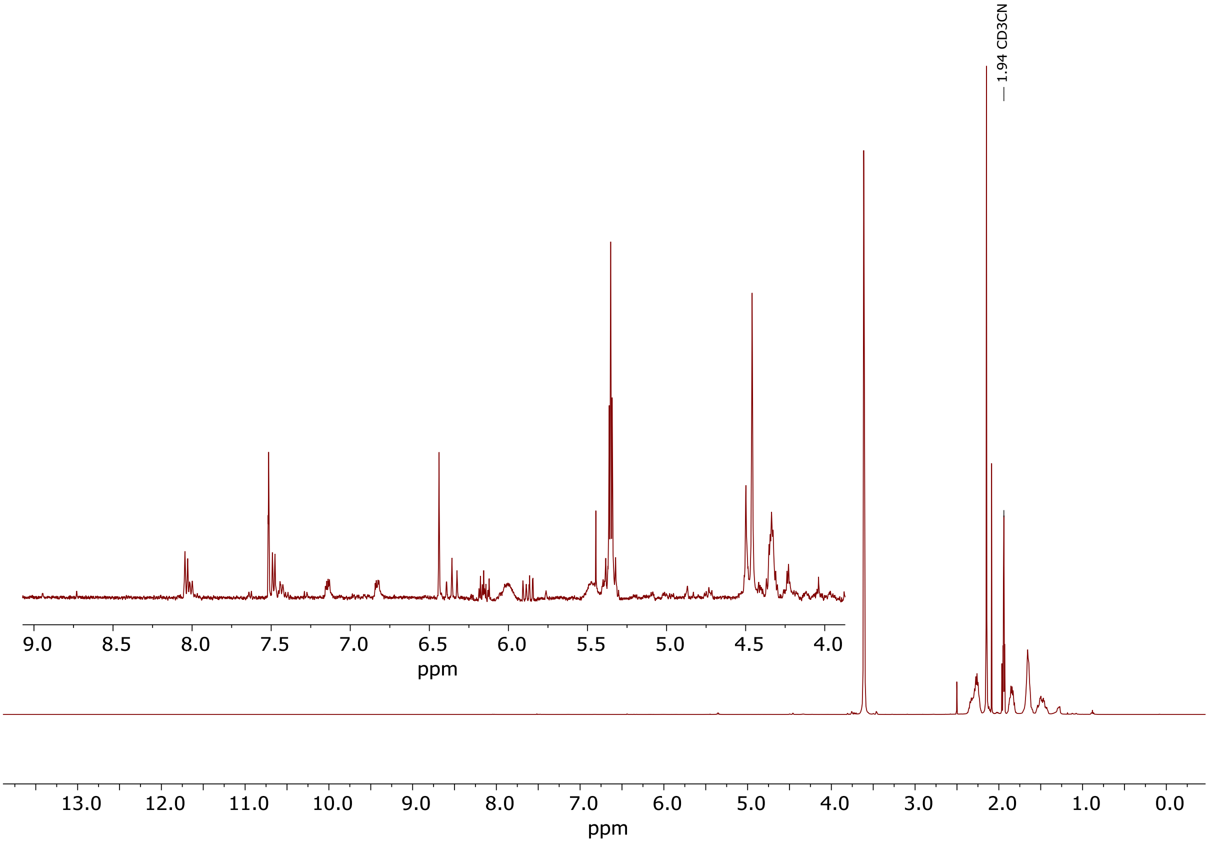

Spectrum S52. ^{1}H NMR (500 MHz, acetonitrile-d_3, 298 K, 256 scans) of **P-2** after 240 min of sonication in acetonitrile-d_3 prior to solvent removal.

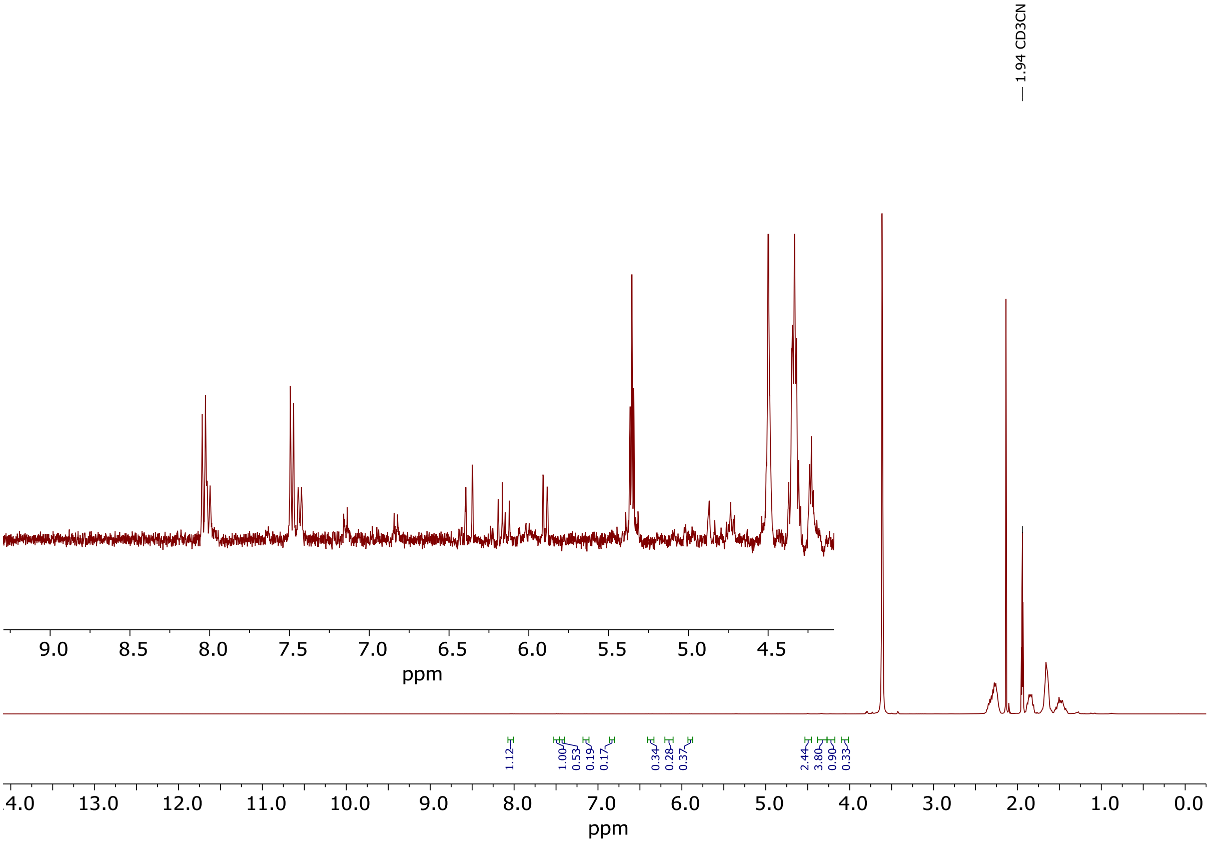

Spectrum S53. ^{1}H NMR (500 MHz, acetonitrile-d_3, 298 K, 256 scans) of **P-2** after 240 min of sonication in acetonitrile-d_3 sample after methanol wash.

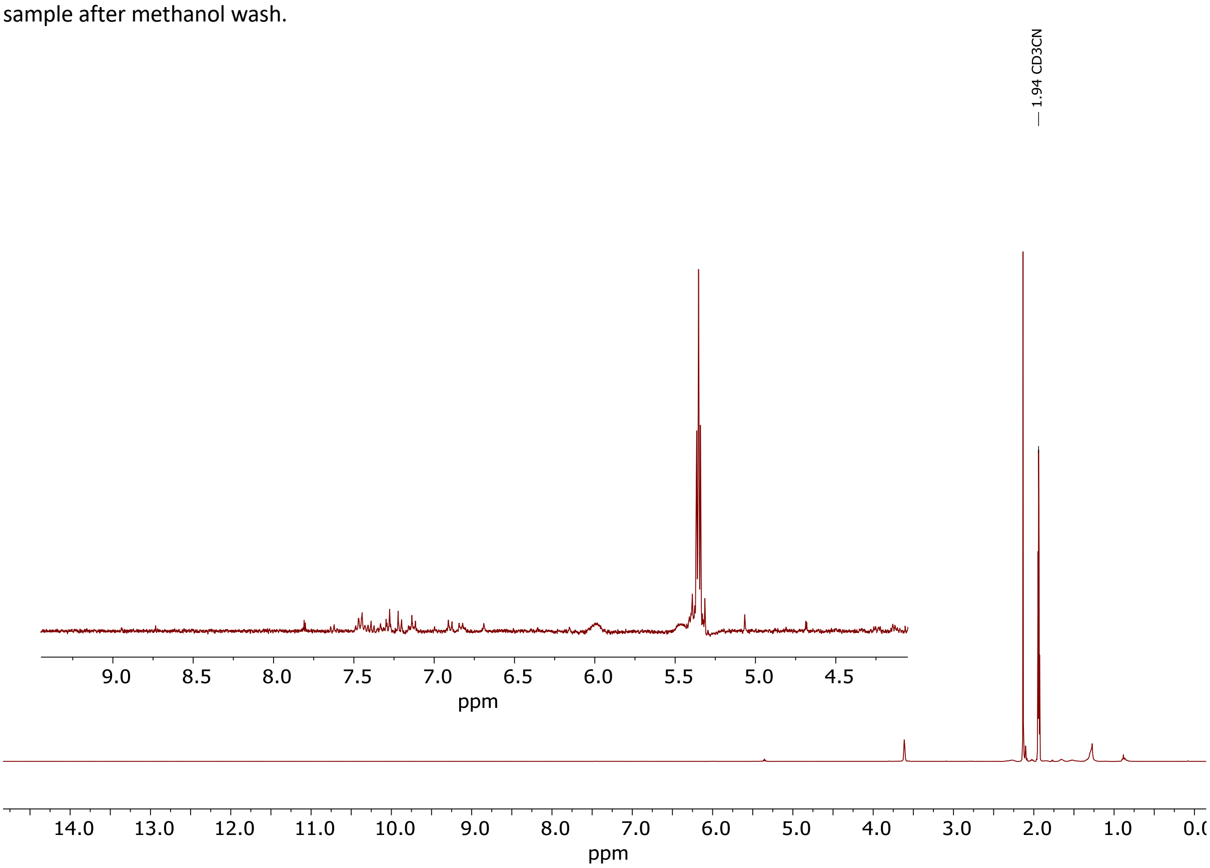

Spectrum S54. ^{1}H NMR (500 MHz, acetonitrile-d_3, 298 K, 256 scans) of methanol wash of **P-2** after 240 min of sonication in acetonitrile-d_3.

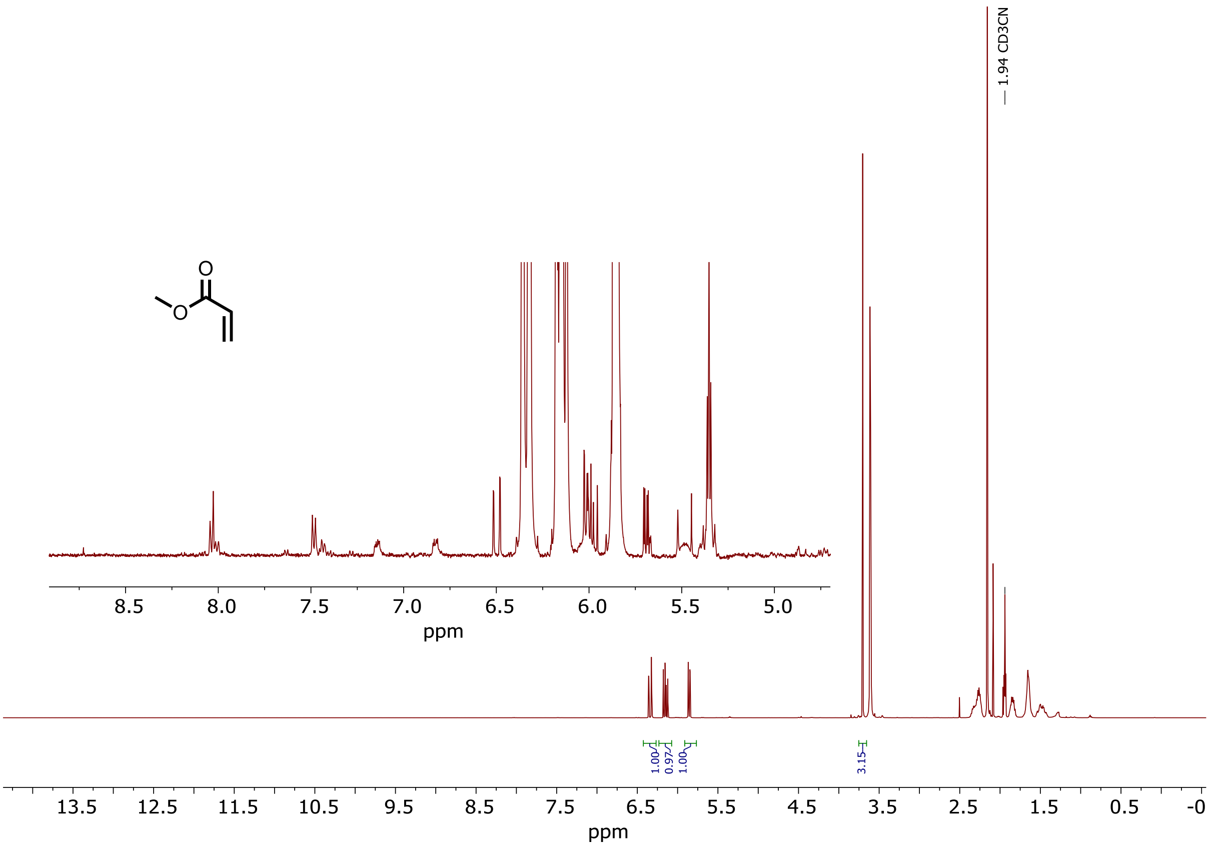

Spectrum S55. ^{1}H NMR (500 MHz, acetonitrile-d_3, 298 K, 256 scans) of methyl acrylate spiked into **P-2** after 240 min of sonication in acetonitrile-d_3.

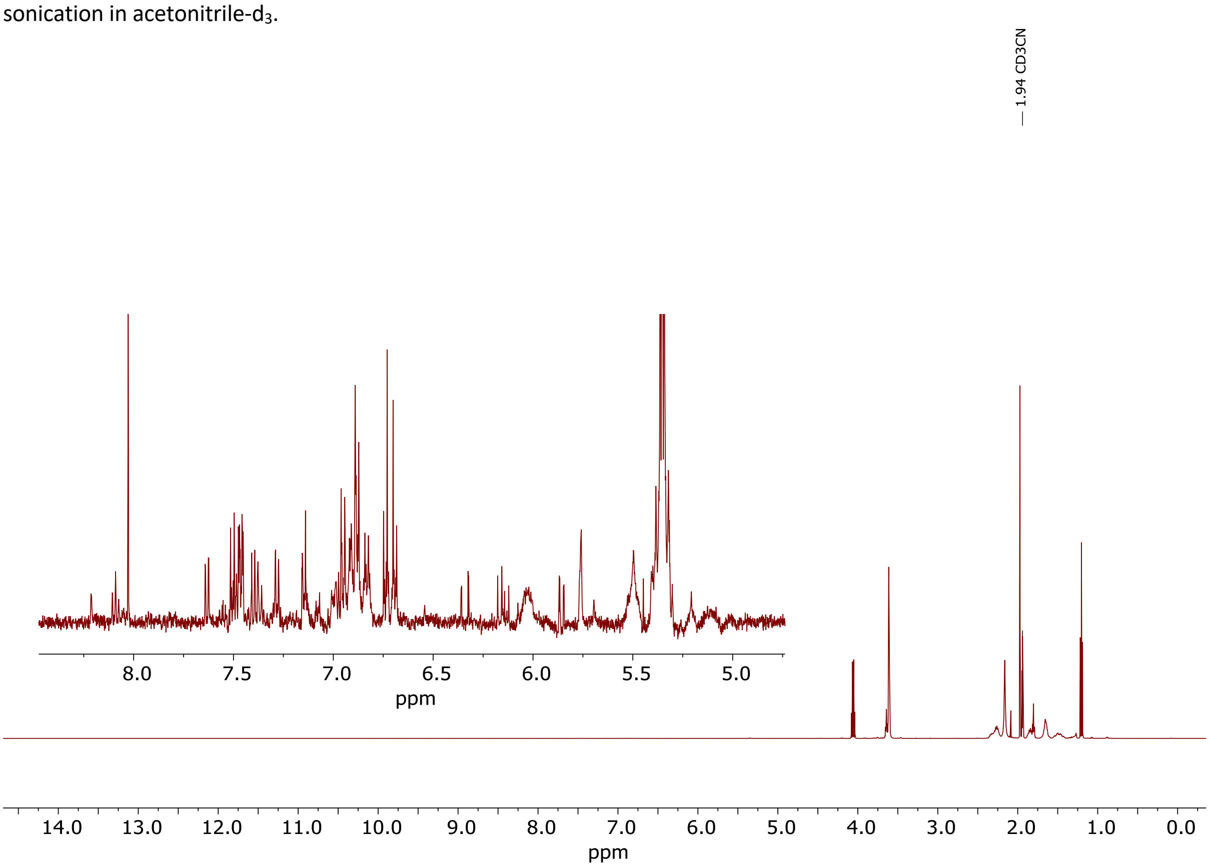

Spectrum S56. ^{1}H NMR (500 MHz, acetonitrile-d_3, 298 K, 256 scans) of **PMA** after 240 min of sonication in acetonitrile-d_3 prior to solvent removal.

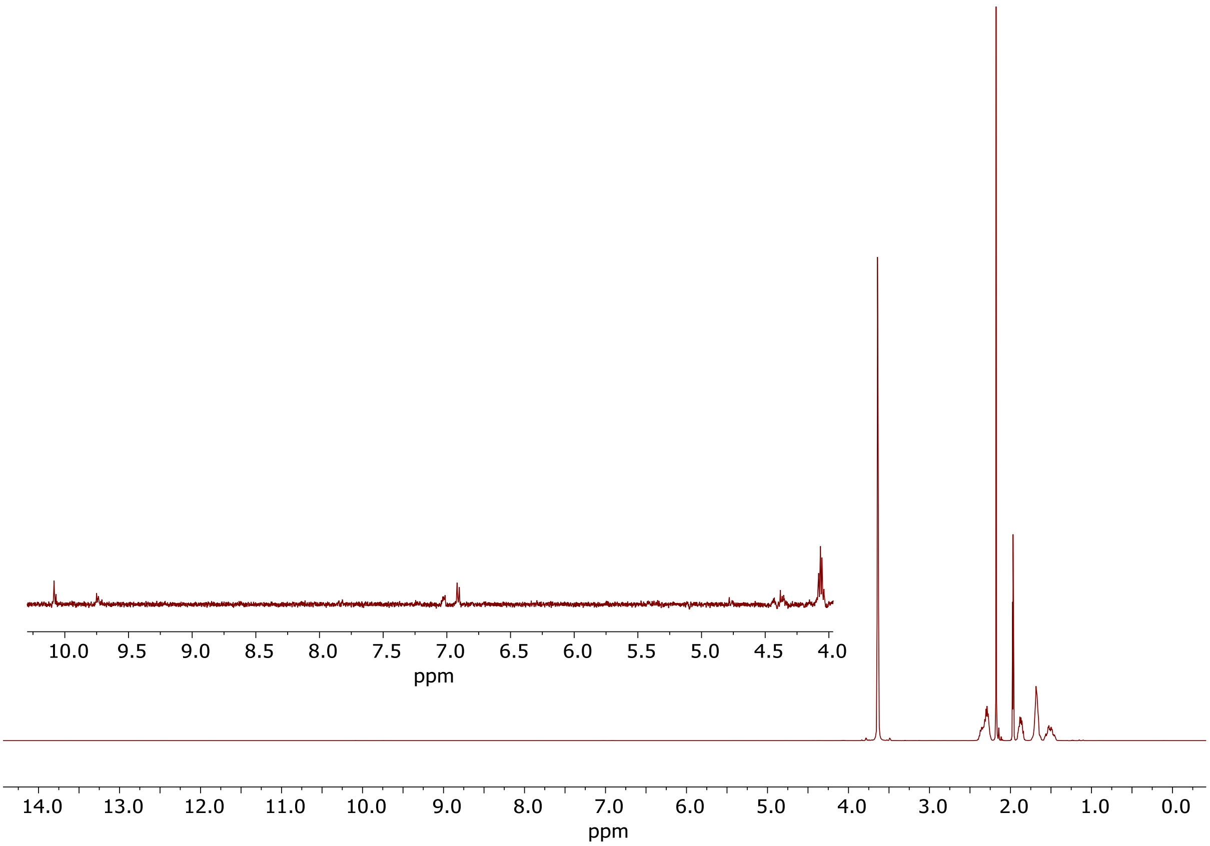

Spectrum S57. ^{1}H NMR (500 MHz, acetonitrile-d_3, 298 K, 256 scans) of **PMA** after 240 min of sonication in acetonitrile-d_3 after methanol wash.

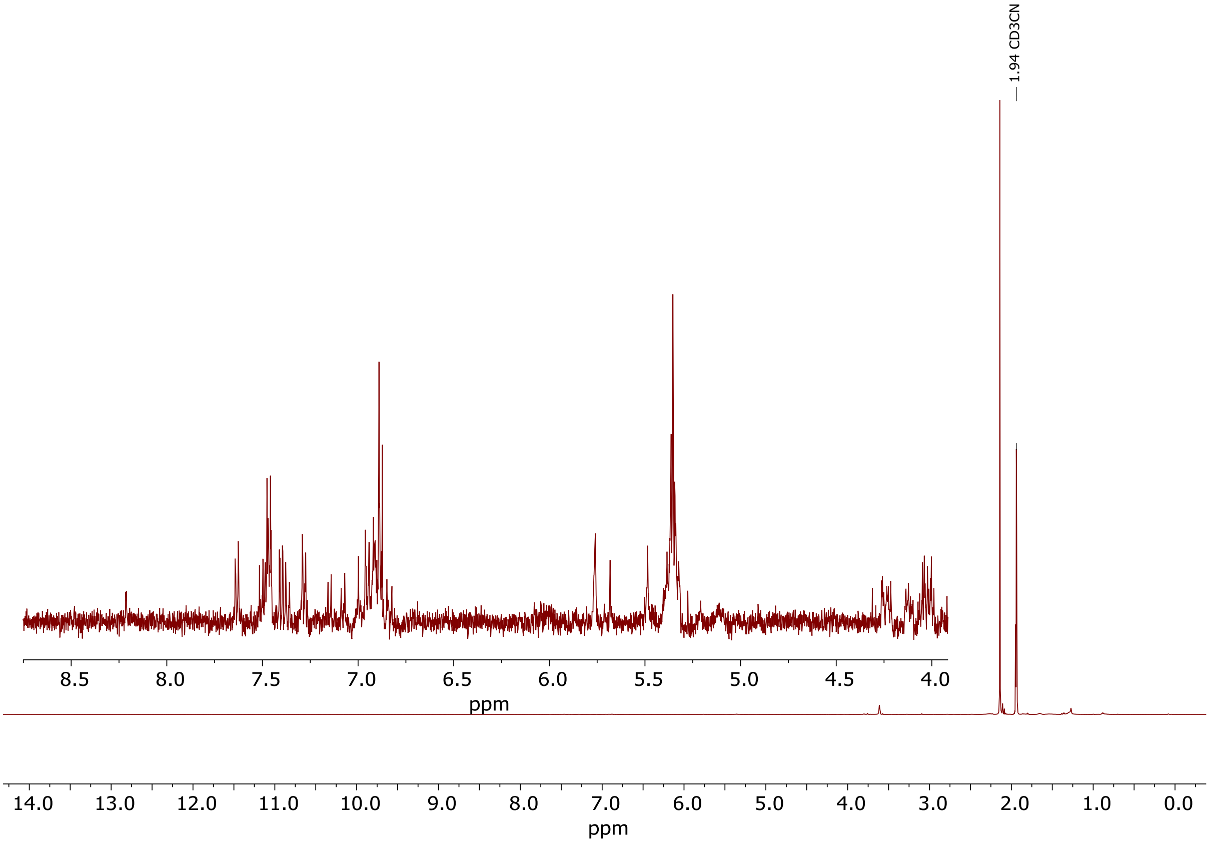

Spectrum S58. ^{1}H NMR (500 MHz, acetonitrile-d_3, 298 K, 128 scans) of methanol wash of **PMA** after 240 min of sonication in acetonitrile-d_3.

7 Thermal activation of polymers

Activation of polymer samples (15 mg) was caried out in toluene (2 mL) at 90°C for 18h under inert nitrogen atmosphere for samples of **P1** – **P4** to yield samples **P1b – P4b**. Upon completion, solutions were allowed to reach room temperature and solvent was removed under reduced pressure. Residual solid (thin film) was washed with methanol and solid polymer sample dried on high vacuum for 2 days before being analysed by NMR and GPC. Sample of **P-S14** was thermally activated to **P-S14b** as described but at temperature of 70°C and for 24h.

7.1 Thermal activation of P-S14

Thermal activation of sample of **P-S14** led to the full thermal retro-[4+2] cycloaddition to release furan and PMA-acrylate species in 24h at 70°C (Figure S27).

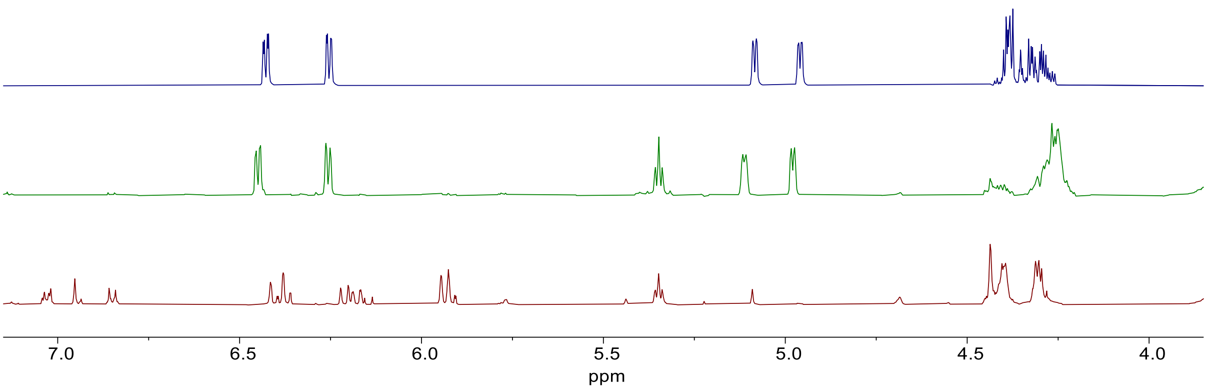

Figure S27. Partial ^{1}H NMR stack (500 MHz, acetone-d_6, 1024 scans) of **S14**, **P-S14** and **P-S14b** (top to bottom, respectively).

7.2 Thermal activation of P-1

Thermal activation of polymer **P-1** led to its slow degradation as is indicative of the formation of the acrylate PMA peaks, as in reference of heated sample of **P-S14b**. Despite high temperature, the mechanophore appears relatively thermally stable, as conversion is only approximately 21% (Figure S28).

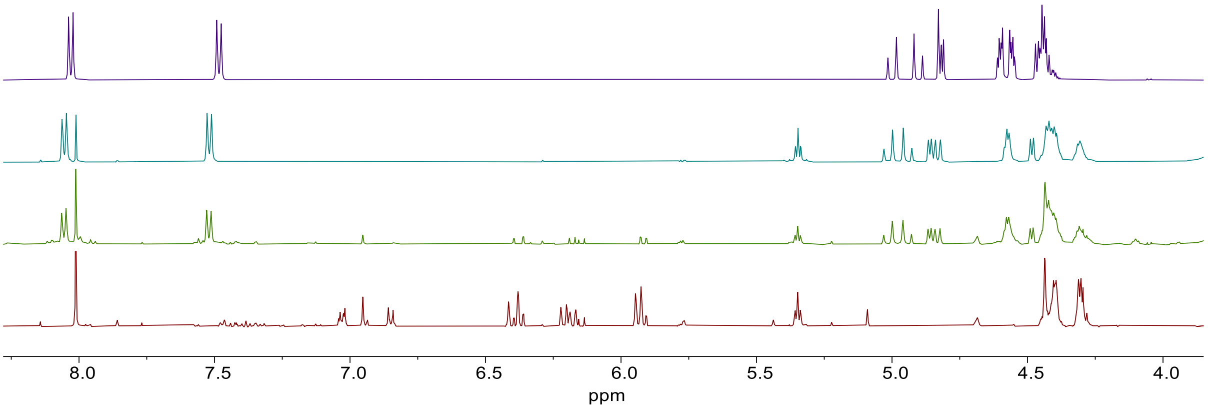

Figure S28. Partial ^{1}H NMR stack (500 MHz, acetone-d_6, 1024 scans) of **1**, **P-1**, **P-1b** and **P-S14b** (top to bottom, respectively).

7.3 Thermal activation of P-2

Thermal activation of polymer **P-2** led to its slow degradation as is indicative of the formation of the acrylate PMA peaks, as in reference of **P-S14b**. Conversion was found to be approximately 21% (Figure S29).

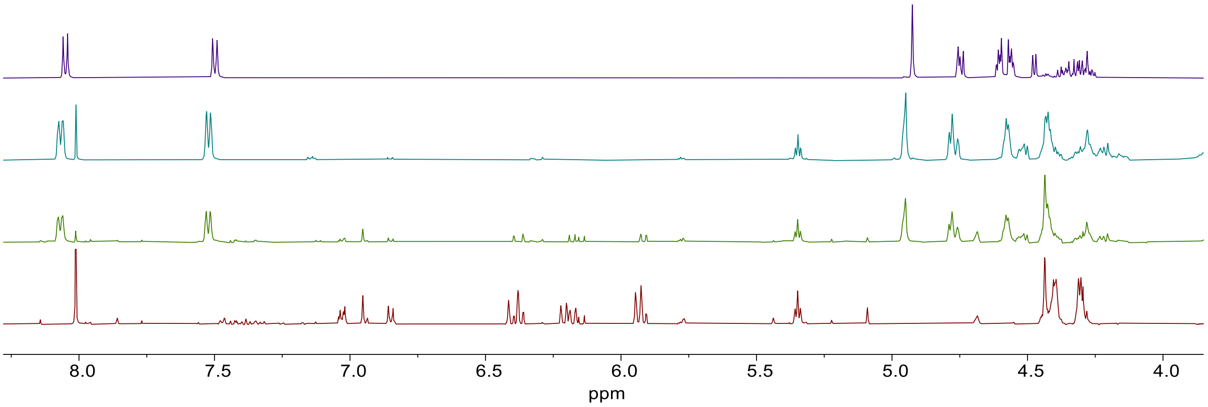

Figure S29. Partial ^1H NMR stack (500 MHz, acetone-d_6, 1024 scans) of **2**, **P-2**, **P-2b** and **P-S14b** (top to bottom, respectively).

7.4 Thermal activation of P-3

Thermal activation of polymer **P-3** led to its slow degradation as is indicative of the formation of the acrylate PMA peaks, as in reference of **P-S15b**. Conversion was found to be approximately 24% (Figure S30).

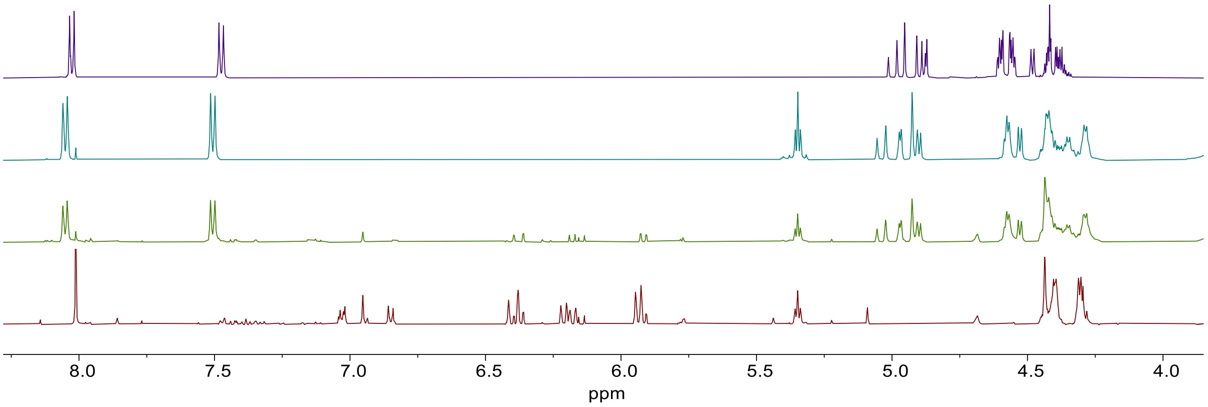

Figure S30. Partial ^1H NMR stack (500 MHz, acetone-d_6, 1024 scans) of **3**, **P-3**, **P-3b** and **P-S15b** (top to bottom, respectively).

7.5 Thermal activation of P-4

Thermal activation of polymer **P-3** led to its slow degradation as is indicative of the formation of the acrylate PMA peaks, as in reference of **P-S15b**. Conversion was found to be approximately 24% (Figure S31).

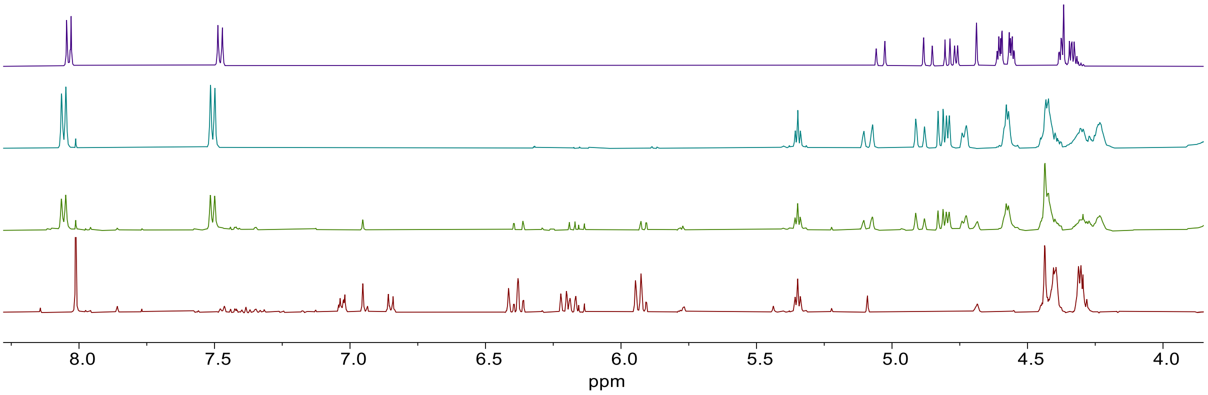

Figure S31. Partial ^1H NMR stack (500 MHz, acetone-d_6, 1024 scans) of **4**, **P-4**, **P-4b** and **P-S15b** (top to bottom, respectively).

8 Crystal structures

Single crystals of compounds **S8 – S11** were obtained by diffusion of hexanes into a concentrated solution of a given compound in ethyl acetate over 10 days.

8.1 Crystal structure of S8

Table S3. Summary of crystal data for compound **S8**.

CCDC deposition number	2256816
Identification code	**S8**
Empirical formula	$C_{17}H_{19}N_3O_5$
Formula weight	345.35
Temperature/K	100
Crystal system	triclinic
Space group	P -1
a/Å	6.0521 (2)
b/Å	8.5922 (4)
c/Å	15.9355 (5)
α/°	98.929 (3)
β/°	92.896 (3)
γ/°	99.433 (3)
Volume/Å^3	805.09 (5)
Z	2
ρ_{calc} g/cm^3	1.425
μ/mm^{-1}	0.889
F(000)	364.0
Radiation	CuKα (λ = 1.54184)
2Θ range for data collection/°	75.972
Goodness-of-fit	1.064
R (reflections)	0.0346 (2958)
wR2 (reflections)	0.0907 (3135)

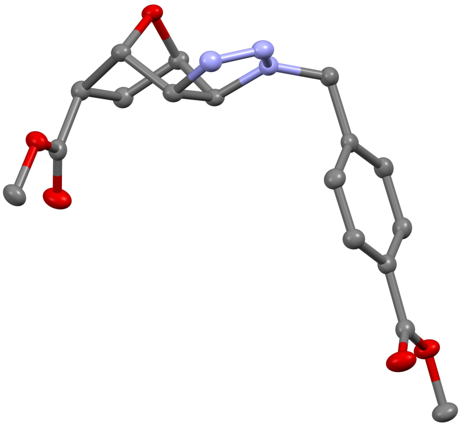

Crystal structure of **S8** showing 50% thermal ellipsoid probability.

8.2 Crystal structure of S9

Table S4. Summary of crystal data for compound **S9**.

CCDC deposition number	2256817
Identification code	**S9**
Empirical formula	$C_{17}H_{19}N_3O_5$
Formula weight	345.36
Temperature/K	100
Crystal system	monoclinic
Space group	I 2/a
a/Å	16.5754 (4)
b/Å	6.11189 (15)
c/Å	31.8518 (6)
α/°	90
β/°	91.277 (2)
γ/°	90
Volume/Å^3	3226.01 (13)
Z	8
ρ_{calc} g/cm^3	1.422
μ/mm^{-1}	0.888
F(000)	1456.0
Radiation	CuKα (λ = 1.54184)
2Θ range for data collection/°	76.482
Goodness-of-fit	1.085
R (reflections)	0.0355 (2857)
wR2 (reflections)	0.0885 (3193)

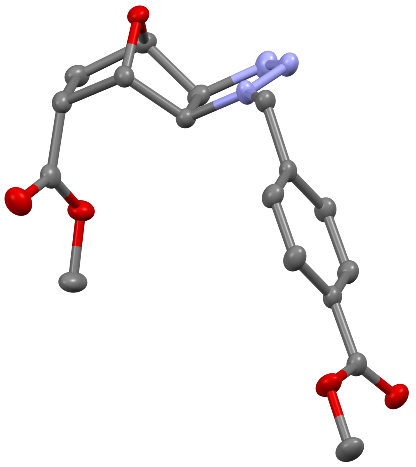

Crystal structure of **S9** showing 50% thermal ellipsoid probability.

8.3 Crystal structure of S10

Table S5. Summary of crystal data for compound **S10**.

CCDC deposition number	2256818
Identification code	**S10**
Empirical formula	$C_{17}H_{19}N_3O_5$
Formula weight	345.36
Temperature/K	100
Crystal system	triclinic
Space group	P -1
a/Å	5.1852 (1)
b/Å	5.8790 (2)
c/Å	27.6981 (6)
α/°	90.105 (2)
β/°	92.393 (2)
γ/°	107.615 (2)
Volume/Å^3	803.96 (4)
Z	2
ρ_{calc} g/cm^3	1.427
μ/mm^{-1}	0.891
F(000)	364.0
Radiation	CuKα (λ = 1.54184)
2Θ range for data collection/°	76.174
Goodness-of-fit	1.063
R (reflections)	0.332 (3112)
wR2 (reflections)	0.0836 (3188)

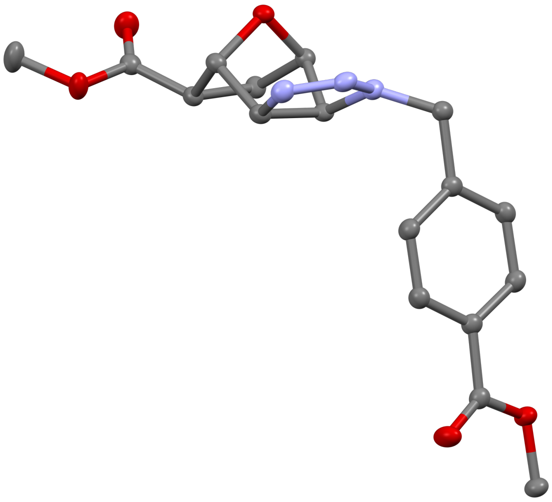

Crystal structure of **S10** showing 50% thermal ellipsoid probability.

8.4 Crystal structure of S11

Table S6. Summary of crystal data for compound **S11**.

CCDC deposition number	2256815
Identification code	**S11**
Empirical formula	$C_{17}H_{19}N_3O_5$
Formula weight	345.36
Temperature/K	100
Crystal system	monoclinic
Space group	C c
a/Å	27.7155 (5)
b/Å	5.0942 (1)
c/Å	11.2248 (2)
α/°	90
β/°	94.387 (2)
γ/°	90
Volume/Å^3	1580.17 (5)
Z	4
ρ_{calc} g/cm^3	1.452
μ/mm^{-1}	0.906
F(000)	728.0
Radiation	CuKα (λ = 1.54184)
2Θ range for data collection/°	76.426
Goodness-of-fit	1.095
R (reflections)	0.0276 (3195)
wR2 (reflections)	0.0737 (3206)

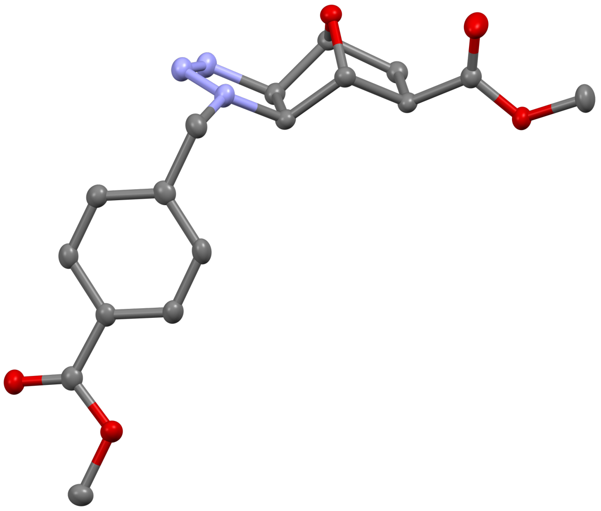

Crystal structure of **S11** showing 50% thermal ellipsoid probability.

9 Calculations

CoGEF calculations were performed on Spartan'20 following Beyer's method.[S4] The structure of the mechanophore was built in Spartan'20 and minimized using molecular mechanics (MMFF). The distance between the terminal methylene groups was constrained and increased by increments of 0.1 Å, and the energy was minimized by molecular mechanics (MMFF) at each step using the *Energy Profile* function implemented in Spartan'20. The geometry of the obtained structures was then minimized by DFT (ωB97X-D/6-31G*, gas) using the *Equilibrium Geometry* function implemented in Spartan'20. The relative energy of each intermediate was determined by setting the energy of the initial state at 0 kJ/mol. F_{max} values were determined from the slope of the final 40% of the energy/elongation curve (i.e. from 0.6 E_{max} to E_{max}).

9.1 CoGEF of model 1' (*endo-exo-trans*)

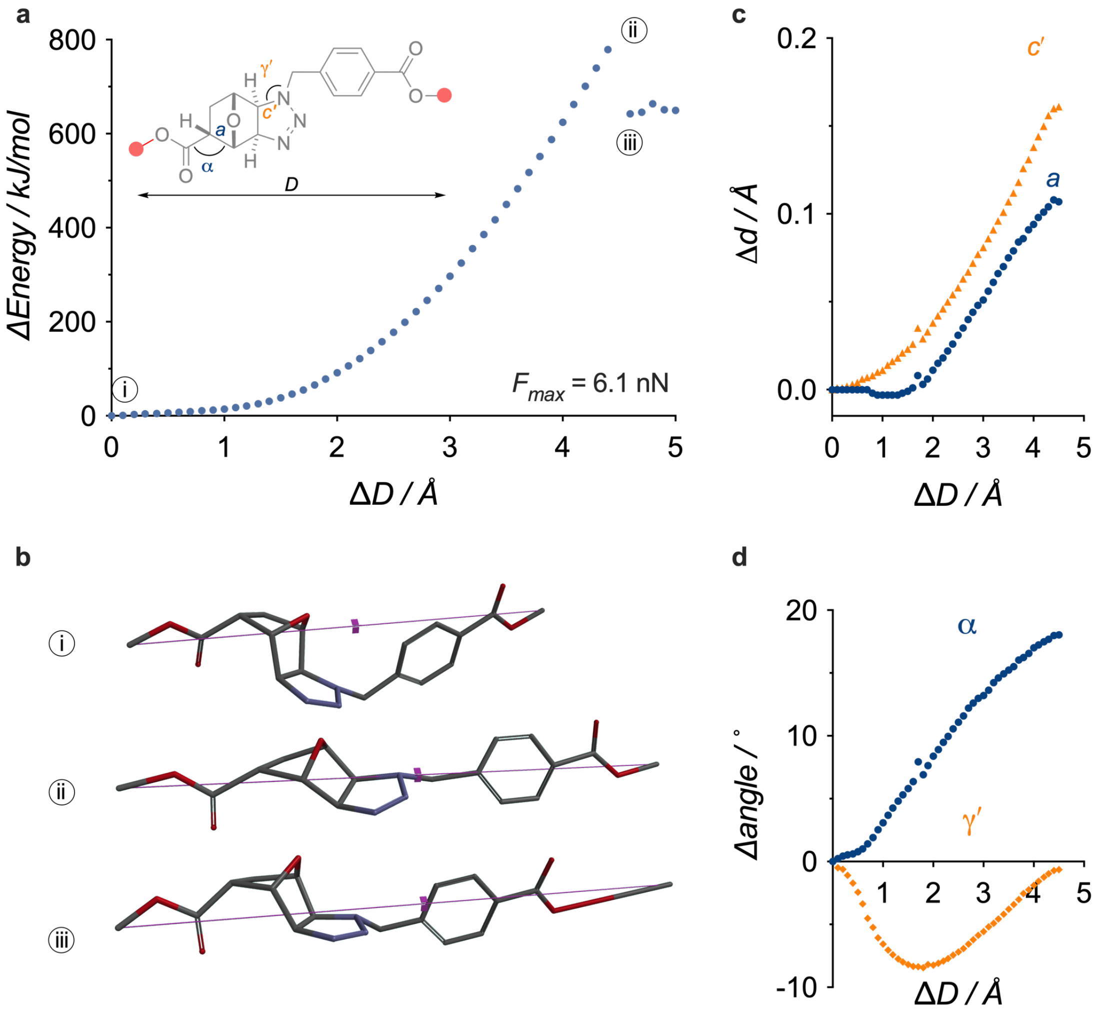

Figure S32. Computational investigation of dissociation of the *endo-exo-trans* model **1'**. (a) Evolution of energy upon simulated elongation (CoGEF, DFT ωB97X-D/6-31G*) of a model **1'**. (b) Equilibrium geometries at E_0 (i), E_{max} (ii), and after bond scission (iii). (c) Elongation of bonds *a* and *c'* and (d) evolution of angles α and γ' upon simulated elongation of the same model. Scissile bonds are marked in red. Pink dots indicate atoms to which constraints have been applied. Hydrogens have been omitted for clarity.

9.2 CoGEF of model 2' and 2'$_{long}$ (*endo-exo-cis*)

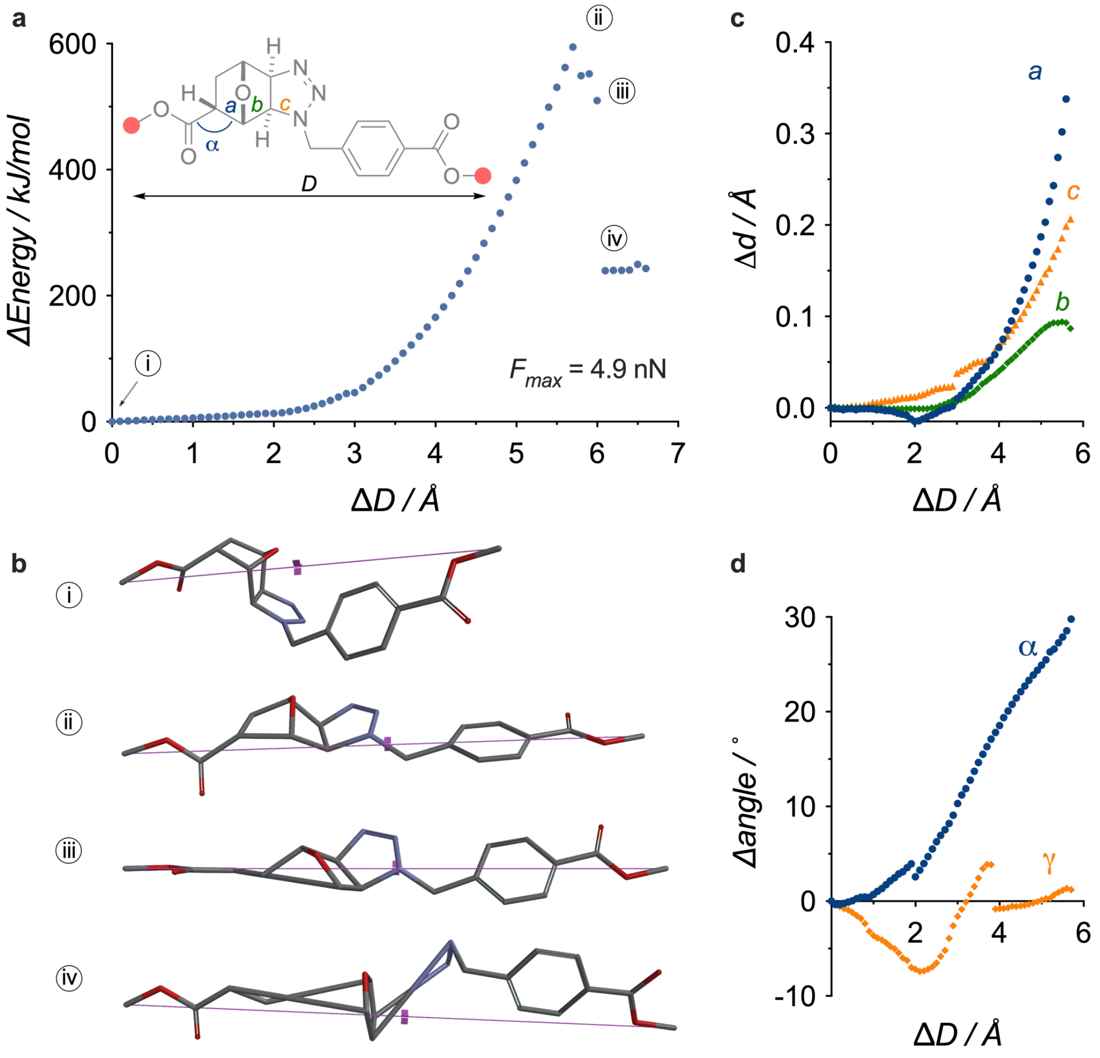

Figure S33. Computational investigation of dissociation of the *endo-exo-cis* model **2'**. (a) Evolution of energy upon simulated elongation (CoGEF, DFT ωB97X-D/6-31G*) of a model **2'**. (b) Equilibrium geometries at E_0 (i), E_{max} (ii), after first bond scission (iii), and after furan release (iv). (c) Elongation of bonds *a, b, c* and (d) evolution of angles α and γ upon simulated elongation of the same model. Scissile bonds are marked in red. Pink dots indicate atoms to which constraints have been applied. Hydrogens have been omitted for clarity.

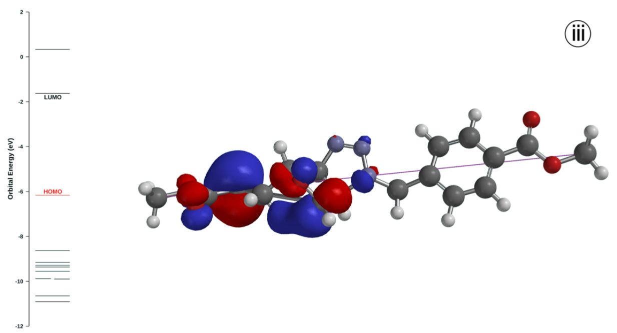

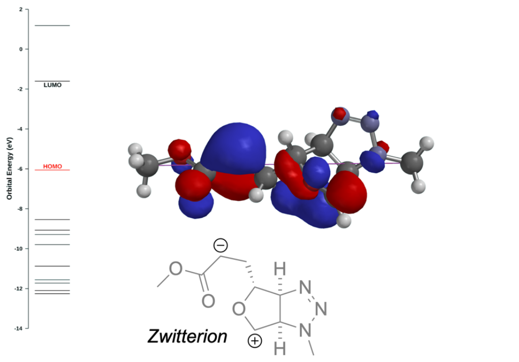

Species	Natural charge at C_1	O_2	C_3
(iii)	-0.401	-0.427	0.188
Zwitterion	-0.407	-0.426	0.192
Diradical	-0.116	-0.537	0.097

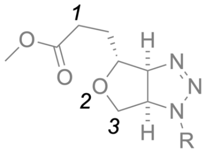

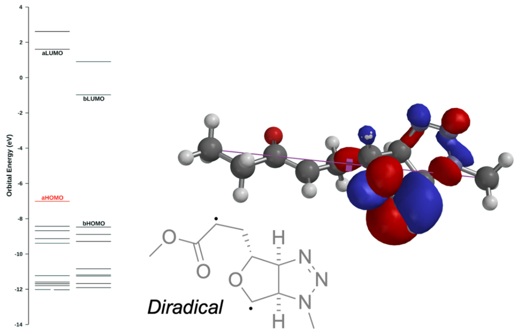

Figure S34. Natural charges at C_1, O_2, C_3 and HOMO orbitals of the first broken intermediates (iii, Figure S33), along with zwitterionic and diradical models, suggest a heterolytic scission (DFT ωB97X-D /6-31G*, gas).

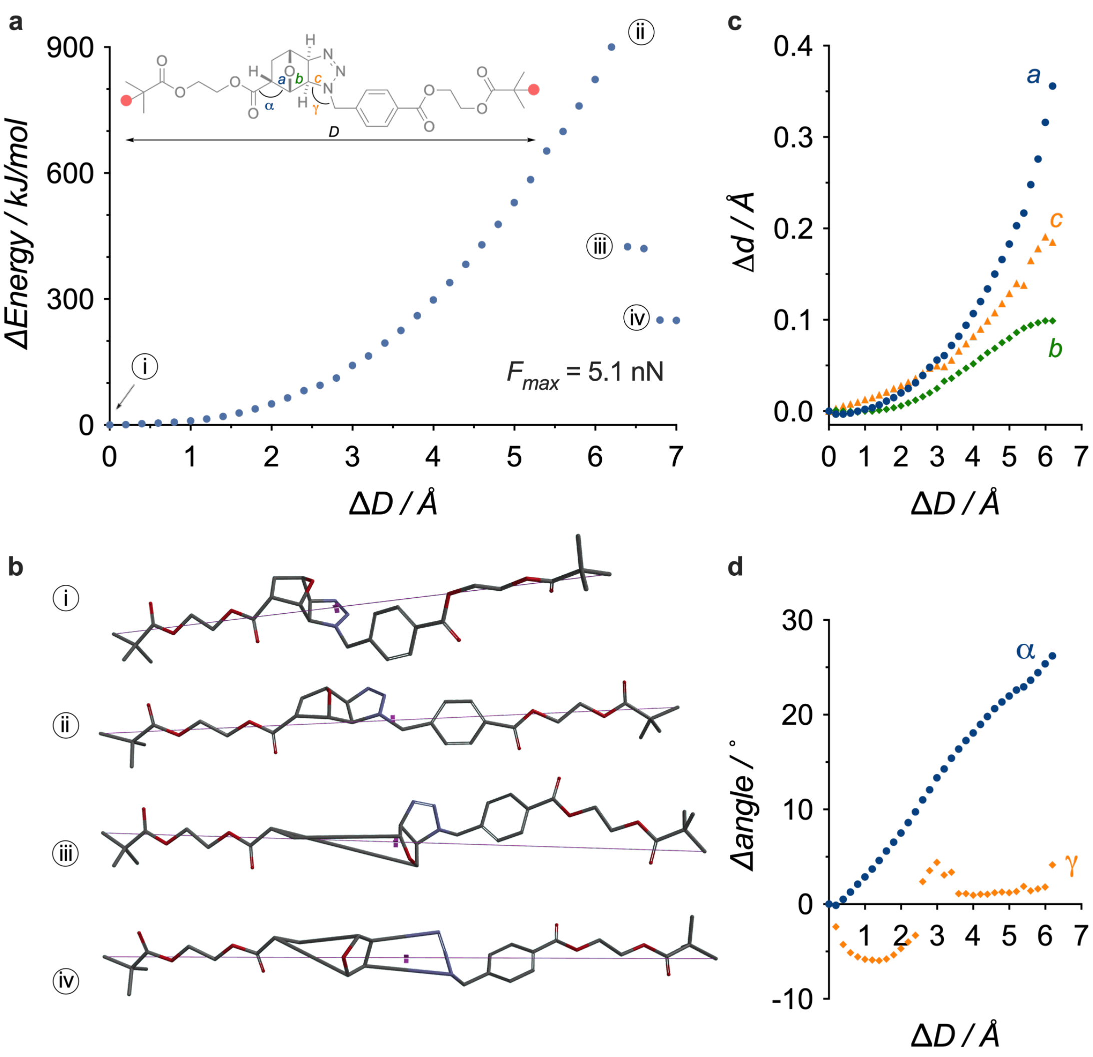

Figure S35. Computational investigation of dissociation of the *endo-exo-cis* longer model **2'**$_{\mathbf{long}}$. (a) Evolution of energy upon simulated elongation (CoGEF, DFT ωB97X-D/6-31G*) of a model **2'**$_{\mathbf{long}}$. (b) Equilibrium geometries at E_0 (i), E_{max} (ii), after first bond scission (iii), and after furan release (iv). (c) Elongation of bonds *a, b, c* and (d) evolution of angles α and γ upon simulated elongation of the same model. Scissile bonds are marked in red. Pink dots indicate atoms to which constraints have been applied. Hydrogens have been omitted for clarity.

9.3 CoGEF of model 3’ (*exo-exo-trans*)

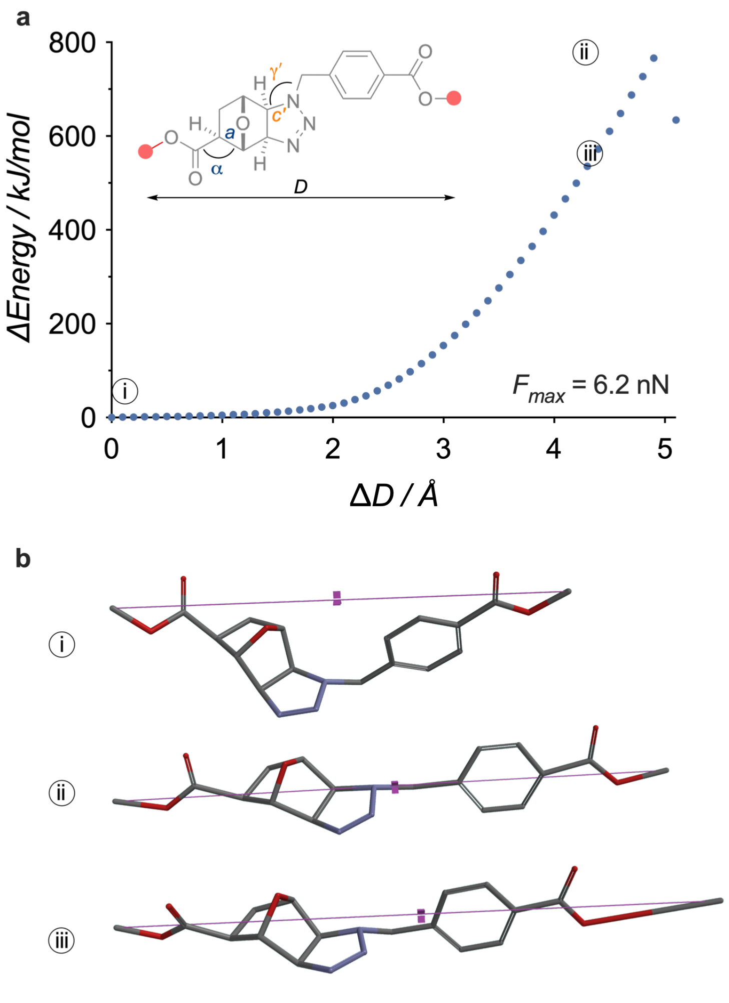

Figure S36. Computational investigation of dissociation of the *exo-exo-trans* model **3’**. (a) Evolution of energy upon simulated elongation (CoGEF, DFT ωB97X-D/6-31G*) of a model **3’**. (b) Equilibrium geometries at E_0 (i), E_{max} (ii), and after bond scission (iii). (c) Elongation of bonds *a* and *c*′ and (d) evolution of angles α and γ′ upon simulated elongation of the same model. Scissile bonds are marked in red. Pink dots indicate atoms to which constraints have been applied. Hydrogens have been omitted for clarity.

9.4 CoGEF of model 4’ (*exo-exo-cis*)

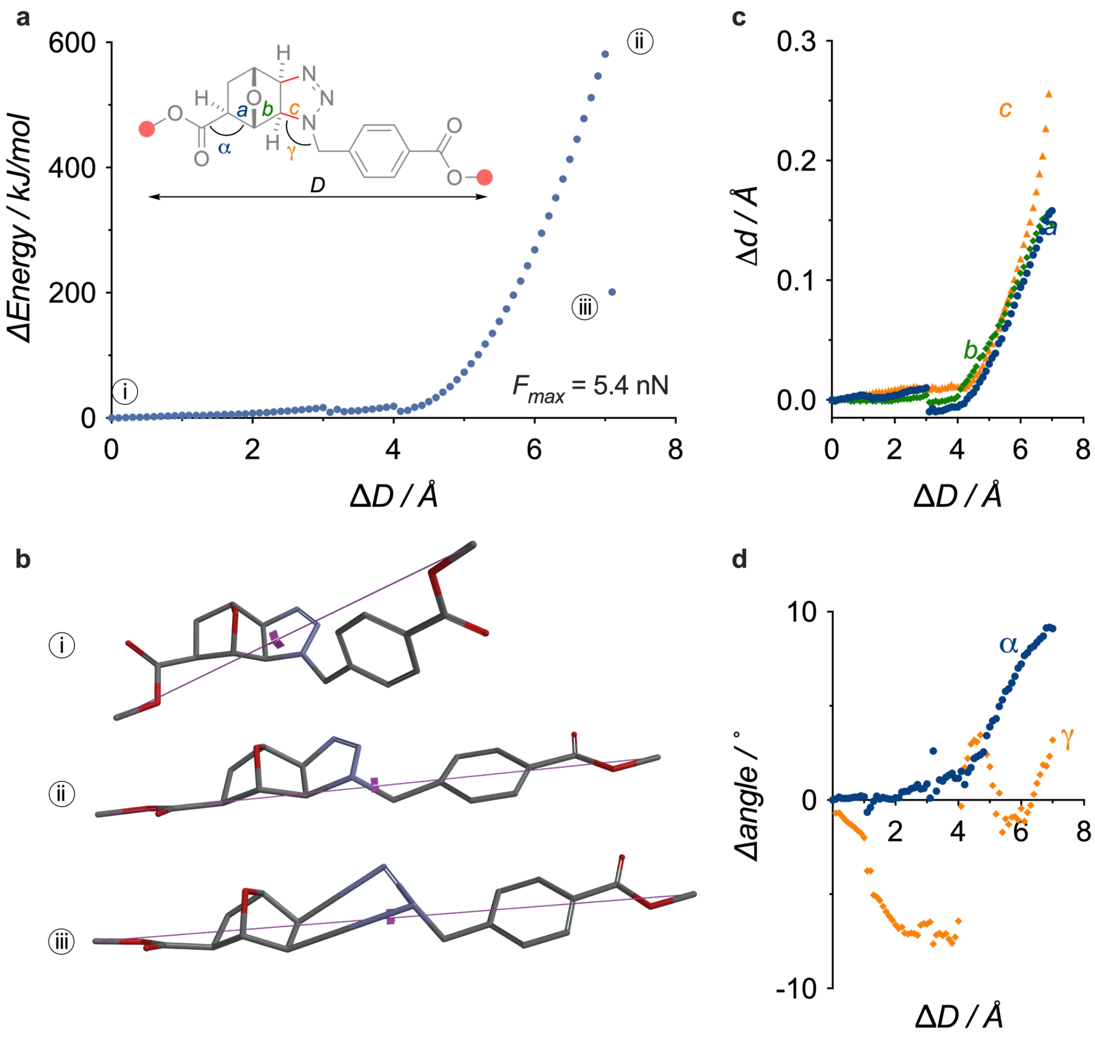

Figure S37. Computational investigation of dissociation of the *endo-exo-cis* model **4’**. (a) Evolution of energy upon simulated elongation (CoGEF, DFT ωB97X-D/6-31G*) of a model **4’**. (b) Equilibrium geometries at E_0 (i), E_{max} (ii), and after bond scission (iii). (c) Elongation of bonds *a, b, c* and (d) evolution of angles α and γ upon simulated elongation of the same model. Scissile bonds are marked in red. Pink dots indicate atoms to which constraints have been applied. Hydrogens have been omitted for clarity.

10 References

(S1) Wang, W.; Zhang, Y.; Sun, B.; Chen, L.-J.; Xu, X.-D.; Wang, M.; Li, X.; Yu,Y.; Jiang, W.; Yang, H.-B. *Chem. Sci.* **2014**, 5, 4554– 4560.

(S2) Hickenboth, C. R.; Moore, J. S.; White, S. R.; Sottos, N. R.; Baudry, J.; Wilson, S. R. *Nature* **2007**, *446*, 423--427.

(S3) Sato, T.; Nalepa, D. E. *J. Appl. Polym. Sci.* **1978**, *22*, 865–867.

(S4) Beyer, M. *J. Chem. Phys.* **2000**, *112*, 7307–7312.
